# Spatial multi-omics identifies early synaptic pruning and context-specific dopaminergic vulnerability in synucleinopathies

**DOI:** 10.1038/s41467-026-74961-6

**Published:** 2026-07-21

**Authors:** Svenja-Lotta Rumpf, Felix L. Strübing, Karsten Nalbach, Claudia Marina Vargiu, Giacomo Berg, Stefan F. Lichtenthaler, Piero Parchi, Pan Gao, Weilin Chen, Matthias Brendel, Johannes S. Gnörich, Alexander Bernhardt, Léa Dias Rodrigues, Günter U. Höglinger, Jochen Herms, Thomas Koeglsperger

**Affiliations:** 1 German Center for Neurodegenerative Diseases (DZNE), Munich, Germany; 2 Munich Cluster for Systems Neurology (SyNergy), Munich, Germany; 3 Department of Neurology, LMU University Hospital, Ludwig-Maximilians-Universität München, Munich, Germany; 4 Neuroproteomics, School of Medicine and Health, TUM University Hospital, Technical University of Munich, Munich, Germany; 5 Laboratorio di Neuropatologia, IRCCS Istituto delle Scienze Neurologiche, Ospedale Bellaria, Bologna, Italy; 6 Department of Biomedical and Neuromotor Sciences, University of Bologna, Bologna, Italy; 7 Department of Nuclear Medicine, LMU University Hospital, LMU Munich, Munich, Germany; 8 Aligning Science Across Parkinson’s (ASAP) Collaborative Research Network, MD 20815 Chevy Chase, USA; 9 Centre for Neuropathology and Prion Research, LMU Munich, Munich, Germany

**Keywords:** Microglia, Parkinson's disease

## Abstract

Parkinson’s disease (PD) is characterized by degeneration of dopaminergic neurons in the substantia nigra pars compacta, but the molecular events preceding neuronal loss remain unclear. Here, we combine spatial transcriptomics, spatial proteomics, and α-synuclein (αSyn) seed amplification assays to profile post-mortem midbrain tissue from controls, incidental Lewy body disease (iLBD), PD, Alzheimer’s disease (AD), and AD with Lewy body pathology (AD + LBP). We find that αSyn seeding activity correlates with dopaminergic neuron loss in PD-spectrum cases but not in AD-associated LBP, indicating disease-context dependent relationships between αSyn pathology and neurodegeneration. In iLBD, before overt substantia nigra Lewy pathology or detectable αSyn aggregation, we detect increased expression of the complement component C1QC together with loss of inhibitory synaptic markers. These findings support early complement-associated remodeling of inhibitory synapses as a potential pathogenic event preceding overt αSyn aggregation and neuronal degeneration in PD.

## Abstract (web summary)

Parkinson’s disease is associated with dopaminergic neuron loss, but the molecular events preceding degeneration remain unclear. Here, the authors show that early complement associated pruning of inhibitory synapses precedes overt α-synuclein aggregation and neuronal loss in prodromal synucleinopathy.

## Introduction

Parkinson’s disease (PD), the most common neurodegenerative movement disorder, is defined by the loss of dopaminergic (DA) neurons in the substantia nigra pars compacta (SNpc)^1^. A pathological hallmark of PD is Lewy body pathology (LBP)—neuronal inclusions composed primarily of misfolded alpha-synuclein (αSyn) along with vesicular and organelle debris^2–4^. Mutations and multiplications in *SNCA*, the gene encoding αSyn, cause familial PD^5–9^, and common variants at this locus are associated with sporadic disease, supporting a central role for αSyn in PD pathogenesis. LBP accumulates in a stereotyped pattern correlated with disease progression^10–13^. According to Braak staging, pathology originates in the olfactory bulb and autonomic nervous system, advancing to the SNpc where neuronal degeneration begins^12^^,^^14–16^.

While LBP is used as a marker of disease progression, it is not always associated with dopaminergic neuron loss or clinical symptoms^16^. Incidental Lewy body disease (iLBD), defined by brainstem-predominant LBP in the absence of clinically established PD, is considered a neuropathological precursor of PD^13,17–23^. It thus provides a unique model to investigate early molecular events prior to the appearance of SNpc LBP. αSyn seed amplification assays (SAAs) have emerged as tools to detect seeding-competent αSyn and are increasingly incorporated into diagnostic criteria^24–26^, informing therapeutic strategies aimed at halting αSyn aggregation^27–29^. However, the role of LBP in driving dopamine neuron degeneration remains uncertain. LBP is more prevalent than clinical PD^21,30–32^, and neuronal loss can occur without LBP^33,34^, while LBP-rich regions may be spared^35^. Moreover, genetic and sporadic cases often show discordance between overt LBP and neurodegeneration^36–39^, and LBP is frequently found in non-PD conditions such as Alzheimer’s disease (AD)^40–43^. These findings suggest that LBP may exert disease context-dependent effects, the mechanisms of which remain poorly understood^44^. Recent single-cell and spatial transcriptomic studies of the human SN have begun to define cell type–specific and regionally resolved molecular alterations in PD, highlighting changes in dopaminergic neurons, glia, and immune-related pathways^45–52^. In parallel, proteomic analyses of SN tissue, primarily based on bulk or laser-capture approaches, have identified disease-associated protein signatures but remain limited in their spatial resolution and integration with transcriptomic and pathological context^53–55^. Here, we combine spatial transcriptomics, spatial proteomics, and αSyn SAA to investigate molecular alterations in the SNpc across controls, iLBD, PD, AD, and AD with Lewy body pathology (AD + LBP). We show that αSyn seeding activity and aggregation correlates with dopaminergic neuron loss in PD-spectrum cases but not in AD-associated Lewy body pathology, indicating context-dependent relationships between αSyn pathology and neurodegeneration. We further identify increased C1QC expression and reduced inhibitory synaptic markers in iLBD prior to overt nigral Lewy pathology. Together, these findings support early complement-associated synaptic remodeling as a potential pathogenic event preceding overt αSyn aggregation and neuronal degeneration in PD.

## Results

**Fig. 1:**
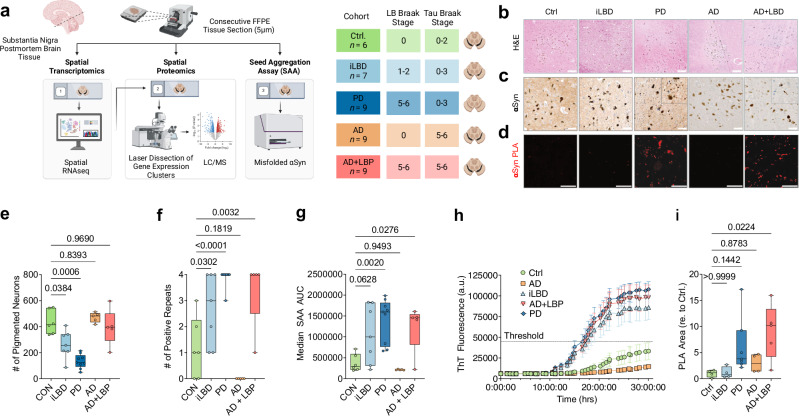
αSyn SAA from FFPE postmortem brain sections predicts LBP. **a** Schematic overview of the workflow and cohorts analyzed: control (Ctrl, *n* = 6), incidental Lewy body disease (iLBD, *n* = 7), Parkinson’s disease (PD, *n* = 9), Alzheimer’s disease (AD, *n* = 5), and Alzheimer’s disease with Lewy body pathology (AD + LBP, *n* = 5). **b** Representative hematoxylin and eosin (H&E) images from each cohort. Scale bar, 100 µm. **c** Representative human αSyn immunostaining from each cohort. Scale bar, 20 µm. **d** Representative αSyn proximity ligation assay (PLA) staining from each cohort. Scale bar, 50 µm. Images in (**b**–**d**) are representative of the respective cohorts and were not necessarily acquired from directly consecutive sections. **e** Quantification of pigmented neurons in the SNpc across cohorts. **f** Number of positive αSyn SAA technical replicates per donor sample. **g** Median area under the curve (AUC) values for the αSyn SAA amplification assay. AUC values were calculated using the trapezoidal method. **h** Mean ThT fluorescence curves over 30 h for each cohort in the αSyn SAA. Lines represent mean values and shaded areas indicate ±SEM from biologically independent donor samples. The dotted line indicates the fluorescence threshold of 50,000 arbitrary units (a.u.) used to define SAA positivity (Ctrl, *n* = 6; iLBD, *n* = 7; PD, *n* = 9; AD, *n* = 5; AD + LBP, *n* = 5). **i** Quantification of αSyn PLA signal across cohorts. For box plots in (**e**–**g**, **i**), center lines indicate medians, box limits indicate the first and third quartiles, whiskers indicate minimum and maximum values, and points represent biologically independent donor samples. Statistical significance in e–g and i was assessed using ordinary one-way ANOVA followed by Dunnett’s multiple-comparison test. Source data are provided as a Source data file. **a** was created in BioRender. Köglsperger, T. (2026) https://BioRender.com/2iuwmtu.

We investigated histological, transcriptomic, and proteomic alterations in consecutive post-mortem FFPE midbrain sections at the level of the SNpc across five distinct cohorts: Ctrl, iLBD, PD, AD, and AD + LBP (Fig. 1a). All tissue samples underwent independent neuropathological examination prior to analysis. The Ctrl cohort (*n* = 6) consisted of individuals without LBP (Braak stage 0), while the iLBD cohort (*n* = 7) presented with Braak stages 1–2 LBP (without SN LBP). The PD cohort (*n* = 9) had Braak stage 5 or higher LBP (incl. SN LBP) (Supplementary Fig. 1a and Table 1). The AD cohort (*n* = 5) included individuals with Tau Braak stages 5–6 and no LBP, whereas the AD + LBP cohort (*n* = 5) exhibited both Tau Braak stage 5–6 and LBP at LBP-Braak stage 5-6. All AD cases were heterozygous for the *APOE* ε3/ε4 genotype (Table 1). The cohorts were comparable in terms of mean age (mean ± SEM: Ctrl, 78 ± 6 years; iLBD, 84 ± 2 years; PD, 80 ± 5 years; AD, 84 ± 3 years; AD + LBP, 81 ± 6 years), with no significant differences between groups (Welch’s ANOVA, *p* = 0.820) (Supplementary Fig. 1b). Sex distribution did not differ significantly across cohorts (Fisher’s exact test, *p* = 0.741) (Supplementary Fig. 1c), whereas fixation times varied significantly between cohorts (Welch’s ANOVA, *p* = 0.038), with mean fixation times (±SEM) of 35 ± 9 days for Ctrl, 52 ± 7 days for iLBD, 42 ± 5 days for PD, and 28 ± 0 days for both AD and AD + LBP (Supplementary Fig. 1d). Post-mortem intervals (PMI) also differed significantly (Welch’s ANOVA, *p* = 0.0004) (Supplementary Fig. 1e), with mean PMIs (±SEM) of 22.17 ± 2.50 h for Ctrl, 27.71 ± 5.13 h for iLBD, 23.00 ± 2.37 h for PD, 4.40 ± 0.24 h for AD, and 5.80 ± 0.86 h for AD + LBP. The shorter PMIs and fixation times in the AD and AD + LBP cohorts were attributed to their sourcing from a different brain bank. Detailed cohort-specific metadata are provided in Table 1.

**Table 1:** Cohort characteristics. Summary of demographic and neuropathological characteristics of the study cohorts

Cohort	*n*	Age, mean ± SEM (years)	Sex (M/F)	PMI, mean ± SEM (h)	Fixation time, mean ± SEM (days)	LBP Braak stage	Aβ stage	Tau Braak stage	APOE genotype
Ctrl	6	78 ± 6	4/2	22.2 ± 2.5	35 ± 9	0	0	0	mixed
iLBD	7	84 ± 2	5/2	27.7 ± 5.1	52 ± 7	1–2	0	0	mixed
PD	9	80 ± 5	6/3	23.0 ± 2.4	42 ± 5	3–6	variable	variable	mixed
AD	5	84 ± 3	2/3	4.4 ± 0.2	28 ± 0	0	high	5–6	APOE ε3/ε4
AD + LBP	5	81 ± 6	3/2	5.8 ± 0.9	28 ± 0	5–6	high	5–6	APOE ε3/ε4

Data are presented as mean ± SEM where applicable. APOE genotype information is summarized at the cohort level to minimize the risk of participant re-identification.

*LBP* lewy body pathology, *PMI* post-mortem interval, *AD* Alzheimer’s disease, *iLBD* incidental Lewy body disease, *PD* Parkinson’s disease.

### Dopaminergic neuron loss and misfolded αSyn pathology across disease cohorts

We quantified pigmented neurons in H&E-stained sections of the SNpc across all cohorts (Fig. 1b). In line with previous reports^21^, cases with iLBD exhibited a significant reduction in pigmented SNpc neurons compared to controls (*p* = 0.038), with further depletion observed in the PD cohort (*p* = 0.0006). Conversely, neuron counts in the AD (*p* = 0.839) and AD + LBP (*p* = 0.969) cohorts did not differ significantly from controls (Fig. 1e).

To evaluate αSyn pathology, we performed immunohistochemical staining against human αSyn. As expected, prominent LBP was observed in PD and AD + LBP cases, while Ctrl, iLBD, and AD cases exhibited only background neuropil staining (Fig. 1c). In addition to traditional immunohistochemical methods, the development of the αSyn SAA enables detection and quantification of misfolded αSyn in human tissue samples^56,57^. Quantification using SAA across all cohorts revealed that all PD cases and all but one AD + LBP case tested positive in all four replicates (4/4) (Fig. 1f). In contrast, iLBD cases showed considerable variability, with individual samples testing positive in only 1 to 4 replicates. Notably, all AD cases tested negative for misfolded αSyn.

When we analyzed the SAA median area under the curve (AUC), substantial variability was observed within the iLBD group, including both SAA-negative and SAA-positive cases, resulting in the loss of significance compared to controls (Fig. 1g). On average, control and AD cases remained below the ThT fluorescence threshold of 50.000 arbitrary units (a.u.), while all other groups tested positive at the group level (Fig. 1h). Given prior evidence suggesting that oligomeric, rather than total, αSyn species may be the principal neurotoxic drivers in PD^58^, we used a proximity ligation assay (PLA)^59^ to detect and quantify oligomeric αSyn in brain tissue sections. In alignment with our SAA findings, robust PLA signals were observed in both PD and AD + LBP cases, indicating a prominent presence of oligomeric αSyn. In contrast, minimal PLA reactivity was detected in other diagnostic groups (Fig. 1d, i and Supplementary Fig. 2).

We next assessed the relationship between αSyn seeding activity, pathological staging, and neurodegeneration by analyzing the correlation between Braak stage, SAA-AUC, and the number of pigmented neurons (Supplementary Fig. 3). Across all cohorts, we observed a strong and significant positive correlation between SAA-AUC and Braak stage (Supplementary Fig. 3a), and a negative correlation between the number of pigmented neurons and both SAA-AUC and Braak stage (Supplementary Fig. 3b, c), indicating that SAA-derived metrics from a single tissue section serve as robust quantitative measures of disease stage in PD. The PLA signal also correlated with SAA-AUC, though to a lesser extent (Supplementary Fig. 3d). When analyses were restricted to PD, iLBD, and Ctrl cases, correlations between pigmented neuron counts and both SAA-AUC and Braak stage were even stronger (Supplementary Fig. 3e, g). In contrast, these associations were attenuated or lost in AD and AD + LBP cases (Supplementary Fig. 3f, h). These findings suggest that the relationship between αSyn and neuropathological staging is context-dependent and more tightly linked to disease progression in the PD spectrum than in the AD setting, setting the stage for a disease-context specific role of αSyn pathology.

To assess whether AD co-pathology contributes to the attenuation of αSyn–neurodegeneration relationships in AD-spectrum cases, we performed multivariable linear regression with pigmented neuron counts as the outcome and SAA median AUC, Braak tau stage, Aβ stage, age, and PMI as predictors (*n* = 27). SAA median AUC remained strongly associated with pigmented neuron loss (*β* = −1.0 × 10⁻⁴, *p* = 0.005), whereas Braak tau stage was not (*p* = 0.88). In contrast, higher Aβ stages were associated with reduced pigmented neuron counts (*p* = 0.007–0.03 across stages). However, the variance in Aβ burden among AD cases was low, as all individuals exhibited high levels of Aβ pathology. The model showed good fit (adjusted *R*² = 0.73). An SAA × Braak tau interaction was not statistically significant (*p* = 0.18), although its directionality was consistent with attenuation at higher tau burden.

### Spatial transcriptomic profiling reveals regional gene expression changes and cellular composition in Parkinson’s disease progression

**Fig. 2:**
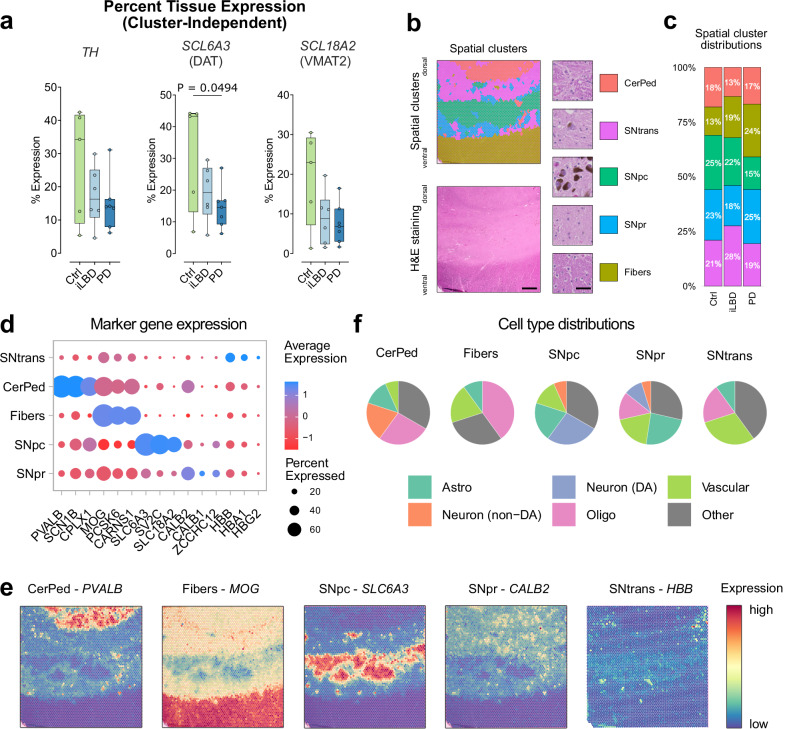
Spatial transcriptomics characterization of LBP-positive and -negative cohorts. **a** Fraction of midbrain tissue expressing the dopaminergic marker genes TH (tyrosine hydroxylase), SLC6A3 (DAT), and SLC18A2 (VMAT2). Data are presented as box plots with center lines indicating means, box limits indicating the first and third quartiles, whiskers indicating minimum and maximum values, and points representing biologically independent donor samples (Ctrl, *n* = 6; iLBD, *n* = 7; PD, *n* = 9; AD, *n* = 5; AD + LBP, *n* = 5). **b** Representative gene clusters delineated across an axial midbrain section. CerPed cerebral peduncle, SNtrans transition zone, SNpc substantia nigra pars compacta, SNpr substantia nigra pars reticulata, Fiber pyramidal tract fiber zone. Scale bar, 1 mm (Insert panel: 20 μm). **c** Percentual distribution of spatial gene clusters across the five different cohorts. **d** Annotation of the relative expression levels of marker genes distinguishing the five spatial clusters. **e** Representative feature plots for the most prominent marker in each cluster. **f** Distribution of deconvoluted cell types per cluster. Statistical significance was assessed using ordinary one-way ANOVA followed by Tukey’s multiple-comparison test in (**a**). Source data are provided as a Source data file.

To investigate the cellular and molecular alterations underlying neuronal loss across the disease trajectory, we performed spatially resolved RNA sequencing on midbrain tissue sections. Quality control (QC) metrics, including sequencing depth, read mapping, and gene detection per spot, were assessed for all samples and are summarized in Supplementary Fig. 4. Multidimensional scaling indicated that global variation in the dataset was associated with PMI and tissue source (brain bank), consistent with the confounding between PMI and Visium kit batch (Supplementary Fig. 5). As anticipated^21^, typical dopaminergic marker genes demonstrated a progressive reduction in expression along the PD trajectory (Fig. 2a and Supplementary Fig. 6). This decrease was significant for *SLC6A3* (encoding the dopamine transporter, DAT) when comparing controls to PD, with a consistent trend observed across the other cohorts.

Following rigorous quality control, which resulted in the exclusion of four samples (1 control, 1 iLBD, 2 PD) due to a low fraction of captured genes, a total of 22 cases (6 controls, 7 iLBD, 9 PD) were subjected to spatial consensus clustering using a Bayesian framework designed specifically for multi-sample analysis^60^. Five spatial clusters were identified and annotated based on their correlation with H&E-stained sections, reflecting the neuroanatomy of the midbrain (Fig. 2b). These clusters included the cerebellar peduncles (CerPed), composed predominantly of axons projecting from the cerebellum to the midbrain and portions of the red nucleus; fibers of the pyramidal and frontopontine tracts (Fibers); the SNpc; the substantia nigra pars reticulata (SNpr); and the transitional zones between these regions (SNtrans).

Dopaminergic neuron loss was evident in the SNpc cluster, with the fraction of tissue occupied by this cluster decreasing from 25% in controls to 22% in iLBD and 15% in PD cases (Fig. 2c). To further investigate the gene expression profiles underlying these spatial clusters, we identified the three most highly and specifically expressed genes per cluster (Fig. 2d, e). *PVALB*, encoding parvalbumin (a calcium-binding protein in GABAergic interneurons), was overrepresented in the cerebellar peduncles, while Myelin Oligodendrocyte Glycoprotein (*MOG*) was prominently expressed in the descending axons of the fiber tracts. The dopamine transporter *SLC6A3* showed the strongest expression in the SNpc, while Calretinin (*CALB2*), a marker of inhibitory neurons^61^, was most specific to the SNpr. The SN transition zone was enriched for hemoglobin-associated genes (*HBB, HBA1*), suggesting a high vascular density supporting SN neurons.

Since spatial transcriptomics typically captures RNA from multiple cells per spot (usually 2–10 cells), we applied a reference-based deconvolution algorithm to estimate the cell-type composition of each spot (Fig. 2f). Using human midbrain-specific single-cell gene expression data from Kamath et al.^45^, which included 68 sub-clustered cell types, we identified the top 5 cell types per cluster and merged redundant sub-clusters (e.g., Olig_ENPP6_EMILIN2 and Olig_PLXDC2_SFRP1 were combined into “Oligo”; see Source data file for a complete list of all 68 cell types). The top 5 cell types represented more than 60% of the cell types in each cluster, with all other cell types grouped as “Other.” Oligodendrocytes were predominant in the CerPed and Fiber clusters, while dopaminergic neurons were enriched in the SNpc. The SNpr primarily contained astrocytes, with smaller contributions from vascular cells, oligodendrocytes, and a mix of neurons. Finally, the SN transition zone was enriched for vascular/endothelial cells, consistent with the presence of hemoglobin-associated markers.

### Impact of lewy body pathology on regional gene expression

**Fig. 3:**
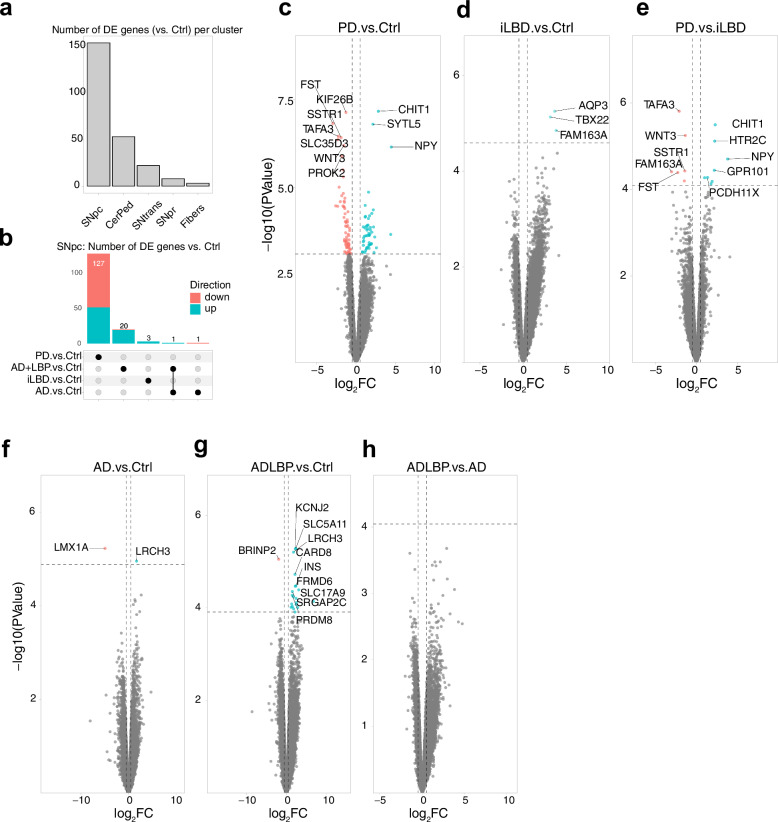
Differential expression analysis of spatial transcriptomic data. **a** Bar graph illustrating the number of DEGs across all clusters in each group compared to Ctrl. **b** Bar graph illustrating the number of SNpc-specific DEGs in different cohorts vs. Ctrl. **c**–**h** Volcano plots depicting DEGs within the SNpc cluster across cohorts compared with Ctrl; key genes with the highest effect sizes (low FDR, high log_2_FC) are highlighted. Source data are provided as a Source data file.

Next, we conducted pseudo-bulk differential gene expression analysis, comparing the log-fold changes between each disease group and Ctrl (Fig. 3a). The most substantial molecular alterations were observed in the SNpc cluster (*n* = 153 differentially expressed genes; DEGs), with fewer DEGs present in the CerPed, SNtrans, SNpr and Fiber clusters (Supplementary Fig. 7). We thus focused on the SNpc and found that most DEGs were present in the PD vs. Ctrl. comparison (*n* = 127), with approximately two-thirds of these genes downregulated in PD (Fig. 3b, c). 20 DEGs were found in the AD + LBP vs. Ctrl. comparison (Fig. 3g), whereas only 3 DEGs could be identified in iLBD vs. control cases (Fig. 3d) and 2 in AD vs. controls (Fig. 3f). Furthermore, certain genes displayed concordant directional changes in both PD vs. Ctrl and PD vs. iLBD comparisons (e.g., *FST, WNT3, CHIT1*, and *NP*Y; Fig. 3e), indicating a common disease trajectory. By contrast, no DEGs were found in the comparison of AD cases with and without LBP, suggesting a disease-context specific effect of LBP on the transcriptomic landscape (Fig. 3h).

### Proteomic profiling reveals distinct molecular signatures in PD and AD + LBP midbrain fiber tracts

**Fig. 4:**
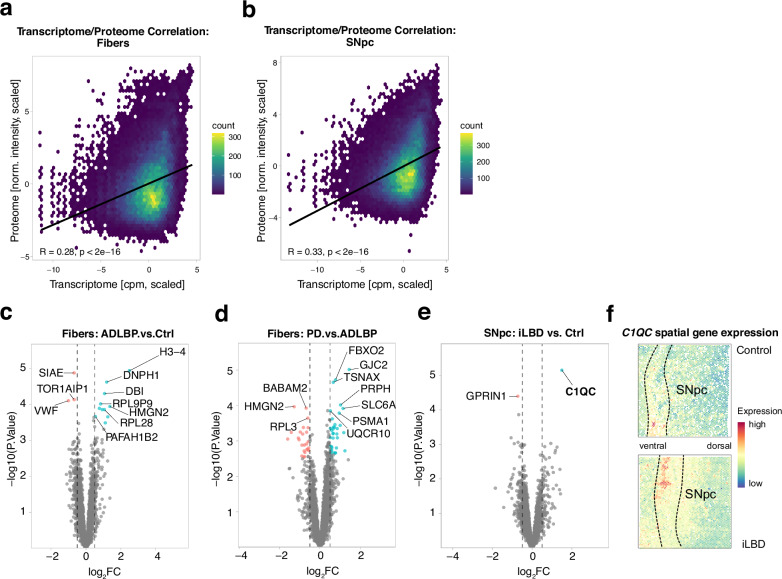
Proteomic characterization of LBP-positive and LBP-negative cohorts. **a**, **b** Correlation between proteome and transcriptome for both clusters that were subjected to LC/MS (Fiber; SNpc). Correlation was assessed using two-sided Pearson correlation analysis. **c**–**e** Volcano Plots of all significantly differentially expressed proteins (Fibers: AD + LBP vs. Ctrl; PD vs. AD + LBP; SNpc: iLBD vs Ctrl). Proteomic data were transformed/processed as described in the methods section. **f** Spatial feature enrichment plot illustrating an increased expression of C1QC in iLBD vs. control sections. Positive log₂ fold-change values indicate proteins enriched in the first group listed in the comparison title, whereas negative values indicate proteins reduced in that group relative to the comparator. Source data are provided as a Source data file.

To capture the protein changes underlying neurodegeneration, we next performed LC/MS-based proteomic profiling of two spatially defined midbrain clusters —SNpc and the ventral fiber tracts—isolated via laser capture microdissection from H&E-stained slides adjacent to the slices used for spatial transcriptomics and SAA. On average, >4000 proteins were identified per sample (Supplementary Fig. 8a; Source data file), aligning with previously reported datasets from post-mortem human tissue^62^. Proteomic quality control analyses showed no clustering by brain bank, fixation time, or post-mortem interval, but instead by tissue type (SNpc vs. fibers) (Supplementary Fig. 9). In agreement with prior findings^63,64^, we observed a moderate but significant positive correlation between transcript and protein abundance, with Pearson’s R values of 0.28 in fiber tracts and 0.33 in SNpc (Fig. 4a, b). While most proteins were shared between clusters, we detected 256 proteins uniquely expressed in the fiber tracts and 605 exclusively in SNpc (Supplementary Fig. 8b).

Differential expression analysis of the fiber tracts^65^ identified significantly altered protein levels (FDR < 0.1, absolute logFC > 0.5) in two contrasts: 14 proteins were differentially expressed in AD + LBP vs. Ctrl. (Fig. 4c), whereas 62 proteins were altered in PD vs. AD + LBP (Fig. 4d). Notably, no other group comparisons pertaining to the SNpc cluster yielded significant proteomic changes (Supplementary Fig. 10). However, the high number of differentially expressed proteins between PD and AD + LBP—two conditions with similar LBP burden (LBP Braak stage 5–6)—was unexpected, prompting further investigation.

### Early synaptic dysfunction in iLBD: C1QC-associated tagging of inhibitory synapses in the SNpc

We next investigated DE proteins in the SNpc. Among all group comparisons, only the iLBD vs. Ctrl contrast yielded significantly altered proteins with strict quality controls, with one upregulated and one downregulated protein (Fig. 4e). The downregulated protein in iLBD was GPRIN1 (G-protein-regulated inducer of neurite outgrowth), a growth cone-associated protein involved in cytoskeletal remodeling and axonal pathfinding^66^. In contrast, Complement C1Q C-Chain (C1QC) was upregulated nearly 2.5-fold in iLBD. C1QA, along with C1QB and C1QC, forms C1Q—the initiating component of the classical complement pathway. C1Q is often repurposed in the brain for glia-mediated synaptic pruning^67^. Its known increase with age is likely due to accumulation of C1Q protein released by microglia onto perisynaptic sites^68^. While C1Q transcripts were not significantly elevated in this comparison after correcting for multiple comparisons, iLBD samples showed a broader and more dispersed gene expression on spatial feature plots with a ventral SNpc predominance (Fig. 4f). C1Q-mediated synaptic tagging is an early event in neurodegeneration^69^, and the increased C1QC abundance suggests that iLBD may be characterized by enhanced synaptic pruning in the SNpc, setting the stage for subsequent neuronal loss. This phenomenon occurred even in the absence of overt LBP in the SNpc (Fig. 1c, d), reinforcing the concept that synaptic dysfunction and neuronal attrition precede LBP^70,71^.

**Fig. 5:**
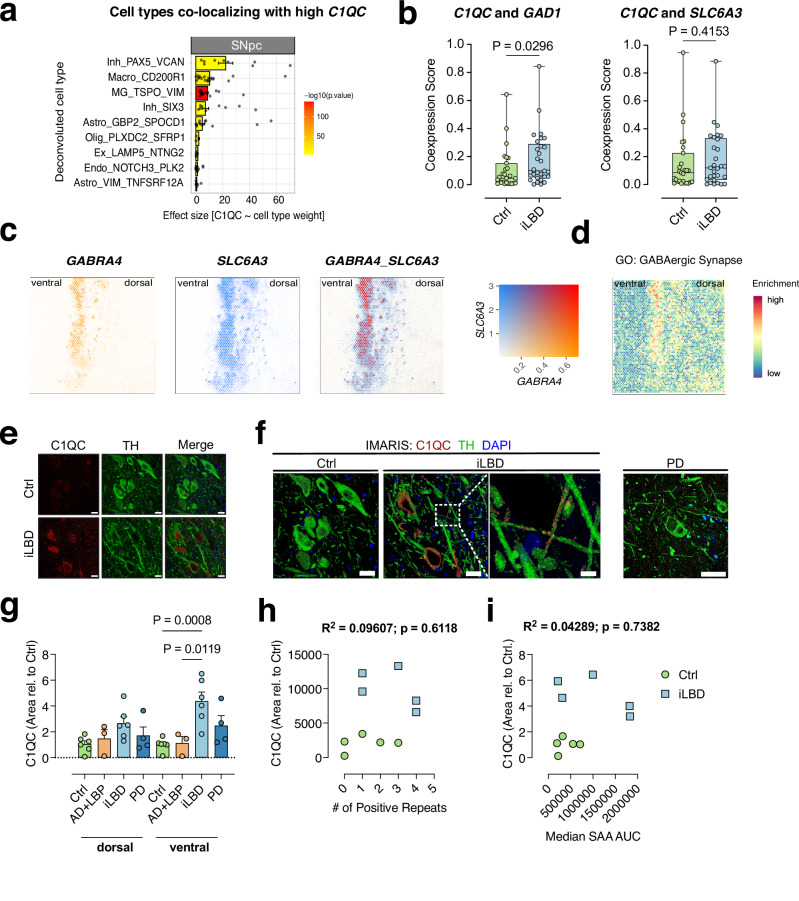
C1QC and co-expression of markers for inhibitory neurons. **a** Results from linear models delineating the cell-type contribution to high C1QC expression after RCTD-based deconvolution. Cell types are ordered according to their statistical contribution (“Estimate”) to elevated C1QC expression. Bars represent regression estimates ± standard error of the coefficient (SE_ß_), and individual points represent biologically independent donor samples with at least one count of C1QC in the deconvoluted cell type (Visium spot). Statistical significance was assessed using two-sided linear regression models. **b** Box-and-whisker plots demonstrating higher co-expression of C1QC with the inhibitory neuron marker *GAD1* in iLBD compared to Ctrl (*P* = 0.0296). No significant difference in co-expression was observed between the two groups for *C1QC* and *SLC6A3* (*P* = 0.4153). Individual data points represent normalized, additive gene weights for Visium spots with at least one count for both genes (Ctrl, *n* = 27; iLBD, *n* = 29). Center lines indicate medians, box limits indicate the first and third quartiles, whiskers indicate minimum and maximum values, and points represent individual tissue spots. **c** Spatial blended feature plots for *GABRA4* and *SLC6A3* demonstrate an enrichment of *GABRA4* in the ventral SNpc tier. **d** Spatial blended feature plots demonstrating the highest enrichment for genes related to the GABAergic synapse in the ventral SNpc. **e** Representative photomicrographs illustrating C1QC in conjunction with TH in SNpc tissue sections from iLBD and Ctrl cases. Scale bar = 20 µm. **f** IMARIS reconstructed z-stacks from immunofluorescence images of iLBD and Ctrl cases. Scale bar = 10 µm; inset (right) shows a high-resolution view with a scale bar of 4 µm. Colocalization of TH and C1QC reveals dense C1QC puncta on TH-positive cell bodies and axons. For comparison, a representative IHC image from a PD case is shown on the right. **g** Bar graph demonstrating increased C1QC expression in iLBD compared to Ctrl, AD + LBP, and PD in the ventral-tier SNpc. Data are presented as mean ± SEM, and individual points represent biologically independent donor samples (Ctrl, *n* = 6; iLBD, *n* = 6; PD, *n* = 4; AD + LBP, *n* = 3). **h**, **i** Graphs illustrating the correlation between the number of positive replicates or the median SAA AUC and the abundancy of C1QC in the SNpc of Ctrl and iLBD cases as determined by IHC quantification (area rel. to Ctrl). Coefficient of determination (*R*^2^) was used for statistical analysis. Statistical significance was assessed using two-sided Mann–Whitney tests in (**b**). Statistical significance was assessed using ordinary one-way ANOVA followed by Tukey’s multiple-comparison test in (**g**). Source data are provided as a Source data file.

Synaptic elimination following C1Q tagging is thought to be microglia-driven. To explore this, we leveraged deconvoluted spatial transcriptomic data and modeled the relationship between C1QC expression and surrounding cell types. As expected, microglia—particularly the *MG_TSPO_VIM* subtype—were significantly enriched in spots with high *C1QC* expression (*p* = 3.9 × 10⁻¹³⁸) (Fig. 5a). However, the strongest effect size was observed for inhibitory neurons, specifically the *Inh_PAX5_VCAN* subtype (*p* = 2.78 × 10⁻⁵), an association exclusive to the SNpc and absent in other midbrain regions (Fig. 5a and Supplementary Fig. 11). Supporting this, co-expression analysis revealed a significant increase in co-expression of *C1QC* and the inhibitory neuron marker *GAD1* (GAD67) in iLBD compared to controls, whereas no such effect was observed for *C1QC* and the dopaminergic marker *SLC*6A3 (Fig. 5b). Inhibitory neurons are sparse in the SNpc, yet approximately 70% of dopaminergic afferents are GABAergic^72^. Given that axons and synaptic terminals contain cell type-specific mRNAs^73^, we hypothesized that *C1QC* enrichment in inhibitory neurons may reflect selective pruning of inhibitory terminals. The ventral tier of the SNpc is particularly vulnerable to degeneration in PD^74^. To assess whether inhibitory synapses within this region are preferentially affected, we examined the spatial co-expression of the GABAergic marker *GABRA4* and dopaminergic marker *SLC6A3*. Spatial feature plots confirmed that *GABRA4-SLC6A3* co-expression was most pronounced in the ventral SNpc (Fig. 5c). We thus hypothesized that increased *C1QC* in iLBD may reflect preferential tagging of inhibitory synaptic terminals on dopaminergic neurons, particularly within anatomical regions that undergo selective degeneration in PD. Accordingly, a spatial transcriptome co-regulation analysis in iLBD subjects revealed a strong, significant enrichment for the gene ontology term “GABAergic synapse” (adj. *P* = 0.006) in the SNpc, suggesting that the molecular machinery for inhibitory synaptic function was preferentially located within this cluster (Fig. 5d).

### Region-specific upregulation of C1QC in iLBD suggests early inflammatory signaling

To further explore the spatial dynamics of *C1QC* expression within the SNpc in iLBD, we analyzed postmortem midbrain sections from Ctrl, iLBD, and PD cases using confocal microscopy (Fig. 5e, f). Guided by spatial transcriptomic findings that indicated ventral-tier enrichment of *C1QC*, we performed stratified quantification across dorsal and ventral regions of the SNpc. In the dorsal tier, C1QC exhibited a trend toward elevation in iLBD relative to control cases, though the difference did not reach statistical significance (Fig. 5g). In contrast, expression was significantly increased in the ventral tier of iLBD cases (Fig. 5g), implicating region-specific complement activation early in disease progression. Notably, C1QC expression remained unchanged in AD + LBP cases, underscoring the specificity of this molecular change to the iLBD-PD trajectory and not primarily driven by LBP (Fig. 5g). To determine whether C1QC upregulation in the ventral SNpc tier was associated with misfolded αSyn, we examined correlations between C1QC and αSyn SAA metrics, including the number of positive replicates and median AUC values (Fig. 5h, i). No significant correlations were detected.

To corroborate our observations derived from postmortem human brain tissue in an animal model, we stereotactically injected wild-type C57Bl/6J mice with αSyn preformed fibrils (PFFs) into the dorsolateral striatum (DLS)—an established animal model to replicate αSyn pathology and propagation in vivo^75–77^. Three months post-injection, brain sections were assessed by immunohistochemistry for phosphorylated αSyn at serine 129 (pS129-αSyn) and C1QC, demonstrating dispersed abundance of pS129-αSyn in various brain regions. Notably, the cellular localization and the punctate pattern of C1QC closely resembled that observed in human samples (Supplementary Fig. 12a). However, C1QC levels did not differ significantly between PFF- and PBS-injected animals in the striatum (Supplementary Fig. 12b) or the SNpc (Supplementary Fig. 12c). To further evaluate this finding using a distinct model and human-derived material, we exposed acute brain slices from wild-type mice for 6 hrs to amplified αSyn aggregates derived from human LBD postmortem brain tissue (Supplementary Fig. 12d). Consistent with results from the mouse PFF model, C1QC abundance remained unchanged (Supplementary Fig. 12e). To validate these findings in an independent model, we examined C1QC in transgenic mice overexpressing human αSyn under the platelet-derived growth factor B (PDGFB) promoter, a well-established PD model^78^. As in the PFF model, we observed no significant change in C1QC levels in TH-positive SNpc neurons (Supplementary Fig. 13a, b). Collectively, these data support a model in which early, region-specific C1QC upregulation reflects an inflammatory response that precedes overt Lewy body formation and detectable aggregated αSyn by conventional histopathological methods.

### Complement tagging of inhibitory synapses promotes microglial pruning in iLBD

**Fig. 6:**
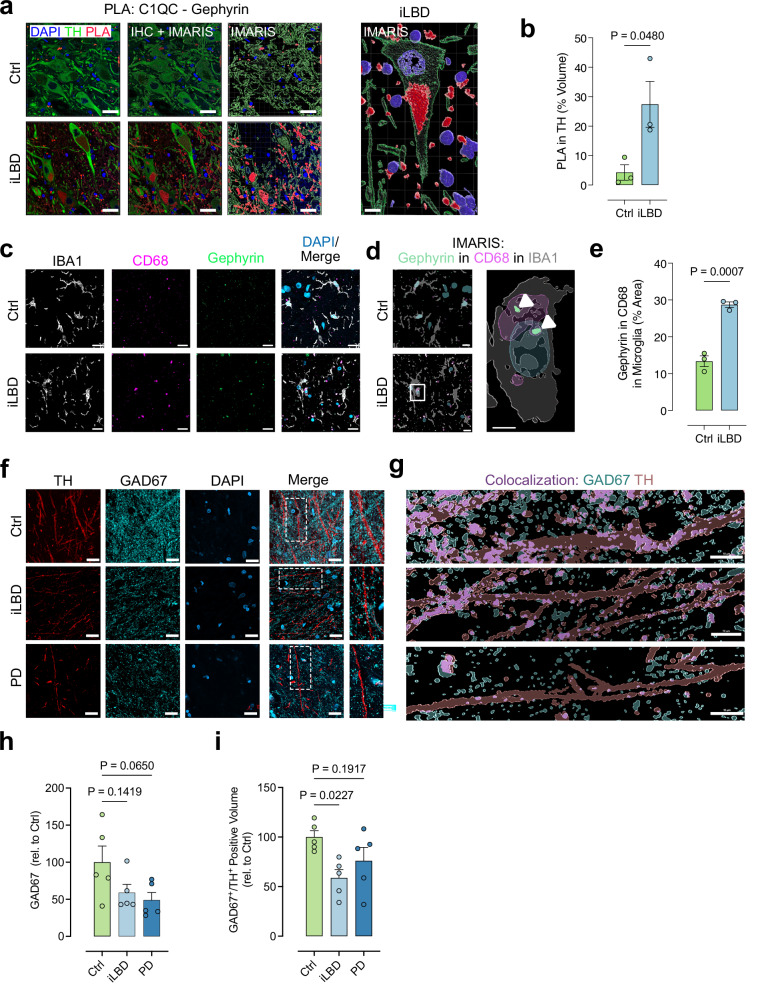
Microglial–synaptic interactions and C1QC–gephyrin colocalization in SNpc of control and iLBD cases. **a** Representative confocal images (3 µm z-stack, five images per stack, three images per case) from Ctrl and iLBD cases illustrating the PLA signal (C1QC–gephyrin interaction, red), TH (green), and DAPI (blue). The left panel shows IHC staining, the middle panel overlays IHC with IMARIS reconstruction, and the right panel displays the IMARIS reconstruction alone (Scale bar = 30 µm). A high-resolution IMARIS reconstruction is shown on the far right. (Scale bar = 10 µm). **b** Quantification of the percentage of PLA signal volume localized within TH-positive neurons in Ctrl and iLBD cases. Data are presented as mean ± SEM, and individual points represent biologically independent donor samples (*n* = 3). **c** Representative photomicrographs illustrating the presence of CD68, IBA1, and gephyrin in SNpc tissue sections from Ctrl and iLBD cases. Scale bar = 15 µm. **d** Merged IHC images (left; Scale bar = 10 µm) and IMARIS reconstructions (right) illustrating the colocalization of CD68 and gephyrin in IBA1-positive microglia. A 3 µm z-stack was acquired with 5 images per z-stack. Scale bar = 3 µm. **e** Quantification of gephyrin within CD68-positive organelles in SNpc microglia from Ctrl and iLBD cases. Data are presented as mean ± SEM, and individual points represent biologically independent donor samples (*n* = 3 per group). **f** Representative photomicrographs illustrating TH and GAD67 immunoreactivity in SNpc tissue sections from Ctrl, iLBD, and PD cases. Scale bar, 20 µm. **g** IMARIS-reconstructed images illustrating TH/GAD67-positive structures in the SN (Scale bar, 10 µm). Bar graphs showing the absolute abundance of GAD67-positive fibers (**h**) and the abundance of GAD67/TH double-positive structures (**i**). Scale bar, 10 µm. Data are presented as mean ± SEM, and individual points represent biologically independent donor samples. Statistical significance was assessed using an unpaired two-sided t-test in (**b**, **e**) and ordinary one-way ANOVA followed by Dunnett’s multiple-comparison test in (**h**, **i**). Source data are provided as a Source data file.

Spatial transcriptomic deconvolution revealed that *C1QC* transcripts were predominantly enriched in microglial clusters, with spatial co-localization near GABAergic transcripts (Fig. 5a, b). To further assess a potential molecular interaction between C1QC and gephyrin (a postsynaptic scaffold protein critical for glycine and GABA A receptor clustering), we performed PLA (Fig. 6a). Confocal imaging (63×, 3-μm z-stack) and IMARIS-based reconstruction revealed a significant increase in PLA signal volume (C1QC-gephyrin interaction) on TH-positive neurons in iLBD tissue compared to controls (Welch’s t-test, *P* = 0.0480; Fig. 6b). Since C1QC-gephyrin clustering points towards an association with GABAergic post-synapses, we next investigated whether microglia actively engage in the phagocytosis of these structures. Immunohistochemistry for IBA1 (a microglial marker), CD68 (a lysosomal marker), and gephyrin revealed gephyrin-postive puncta within CD68-positive compartments in IBA1-positive microglia (Fig. 6c, d). Using ImageJ-based masking, we quantified the gephyrin signal within microglial lysosomes and found a significant increase in the proportion of CD68 compartments containing gephyrin in iLBD cases compared to controls (Welch’s t-test, *P* = 0.0007; Fig. 6e). In contrast to C1QC, CD18—the β2 integrin subunit that forms complement receptors (e.g., CR3 with CD11b) and mediates recognition and phagocytosis of complement-opsonized targets—remained unchanged (Supplementary Fig. 14). These findings suggest that, in the context of iLBD, microglial phagocytosis of GABAergic postsynapses is enhanced, leading to a loss of inhibitory input onto SNpc dopaminergic neurons and, potentially, to their disinhibition. To directly assess this consequence, we stained midbrain sections from Ctrl, iLBD, and PD cases for the GABAergic terminal marker GAD67 and TH to label dopaminergic neurons and their afferents. Consistent with a functional consequence of C1QC-mediated synapse stripping, we observed a reduction in GAD67-positive fibers in iLBD and PD compared with Ctrl cases (Fig. 6f, h, i). IMARIS-based 3D reconstruction further suggested a decrease in TH/GAD67 double-positive structures (Fig. 6g). Collectively, these results indicate that C1QC associates with gephyrin-organized inhibitory postsynapses in iLBD, supporting a model in which complement- and microglia-mediated synaptic remodeling contributes to early dopaminergic circuit dysfunction. Notably, this process appeared to be independent of αSyn aggregation or apparent microglial activation, as no increase in 18-kDa translocator protein (TSPO) signal was observed by autoradiography (Supplementary Fig. 15). These data implicate complement-mediated remodeling of GABAergic synapses as an early and selective mechanism in prodromal dopaminergic circuit vulnerability.

## Discussion

PD is a progressive neurodegenerative disorder associated with a loss of dopaminergic neurons. Despite available symptomatic treatments, no therapy currently stops neuronal loss, and trials of anti-aggregative therapies targeting αSyn remain controversial^27,28^. A major hurdle for developing and testing curative therapies results from the fact that most dopaminergic neurons are already lost at the time of the clinical diagnosis, rendering them inaccessible to therapy. Understanding the early pathological changes that occur before LBP and cell loss in PD would help identify new diagnostic and therapeutic strategies^21^. However, a clear roadmap of these early events and the role of LBP in driving them is currently lacking. In this study, we used a discovery-driven approach, combining spatial transcriptomics, spatial proteomics, and αSyn SAA to examine molecular changes in the midbrain across different stages of αSyn-related disease. By comparing control, PD, and iLBD cases—a potential early stage of PD—we aimed to identify key pathological alterations along the progression of PD and clarify the role of LBP in driving these changes.

The αSyn SAA has recently emerged as a powerful tool for detecting misfolded, pathogenic αSyn species. As a result, the αSyn SAA has gained traction as a novel diagnostic tool for PD, offering high sensitivity and specificity^26^, and, consequently, has been integrated into new biological disease definitions^24,25^. By correlating dopaminergic neuron loss with SAA metrics in consecutive tissue sections, we found that Braak stage and neuronal death correlate strongly with SAA positivity suggesting that SAA provides a molecular readout from postmortem tissue samples that aligns with LBP in PD and iLBD (Fig. 1e–i). This correlation further supports the potential of tissue-based SAAs as a biomarker for neuropathological progression and staging in postmortem settings. While CSF-based SAA is more commonly applied in clinical practice and typically shows higher sensitivity in the presence of neocortical LBP, the present findings highlight the utility of postmortem tissue SAA for assessing regional pathology and disease stage at the neuropathological level^79,80^.

Low-level SAA signal occasionally observed in control samples is consistent with sporadic replicate positivity known to occur due to stochastic self-aggregation of the recombinant substrate^81–83^ and did not meet the predefined replicate-based criteria for positivity; accordingly, these samples were classified as negative, underscoring the importance of replicate concordance rather than reliance on AUC alone when interpreting SAA results (Fig. 1f). Consistent with previous reports, soluble αSyn protein abundance did not differ significantly between PD and control samples, suggesting that pathological changes are driven primarily by aggregation state rather than total protein levels^84^.

In contrast to the prevailing view that LBP co-pathology in AD is largely confined to limbic regions^85,86^, we observed substantial LBP involvement in our AD + LBP cases (Fig. 1d–i). This provides a unique model for distinguishing changes driven by LBP from those arising independently^40–43^. When we investigated AD cases with midbrain αSyn co-pathology, we found no correlation between SAA metrics and dopaminergic neuronal death in AD + LBP cases, despite comparable levels of misfolded αSyn (Fig. 1d–i and Supplementary Fig. 3f, h). Similarly, although AD + LBP cases exhibited a pathological burden comparable to PD (Fig. 1d–i), they showed far fewer molecular changes, with only one gene (*DIRAS2*) exhibiting a similar expression alteration across both groups. Moreover, we identified distinct proteomic signatures in PD compared to AD + LBP, despite similar levels of LBP (Fig. 4c, d). In summary, these findings suggest that the molecular and cellular mechanisms driving disease progression in PD and iLBD are fundamentally distinct from those in AD + LBP, and that the transcriptional impact of LBP in the SNpc is strongly disease-context dependent. Our data indicate that the presence or absence of LBP or misfolded αSyn alone does not predict dopaminergic molecular alterations or cell death, but may reflect disease-specific αSyn variants, including differences in αSyn strains or post-translational modifications^87^. Our data thus provide a deeper understanding of the role of SAA in diagnosing and differentiating αSyn-associated pathologies. However, the lack of significant associations in AD and AD + LBP should be interpreted cautiously given the smaller sample sizes in these groups. These results underscore the complexity of neurodegenerative diseases with overlapping pathologies, support the need for tailoring disease-modifying treatments to the specific pathological context and, raise questions about the theoretical rationale behind non-specific anti-aggregative therapies in PD.

LBP is recognized as the neuropathological hallmark of various neurodegenerative diseases collectively known as synucleinopathies. However, a substantial body of research has raised doubts about the significance of LBP for cellular dysfunction and ultimately, cell death. For instance, Gibb et al. observed an increase in LBP prevalence from 3.8 to 12.8% between the sixth and ninth decade, surpassing the prevalence of clinical PD by three to six times^30,31^. Similarly, a significant proportion of confirmed LBD cases never exhibited clinical symptoms, though undetected or subclinical features cannot be fully excluded^32^. Conversely, some PD cases with specific genetic mutations exhibit marked dopamine neuronal loss despite sparse Lewy bodies (LBs) on conventional histopathology, although recent work has revealed PLA-detectable αSyn inclusions in a subset of such cases^88–91^. Moreover, neuropathological studies have shown that LBs do not correlate well with dopamine neuronal dysfunction and death^33–35,39,92–96^. Taken together, these findings raise doubts about the significance of LBP as the sole cause of cell death in PD and point towards additional disease processes independent of LBP (reviewed in ref. ^21^).

A limited number of conceptual approaches have attempted to dissect LBP-dependent from LBP-independent changes. Although these studies, including our own previous work^97,98^ clearly suggested LBP-independent mechanisms, they relied on hypothesis-driven approaches that focused on individual molecules or pathways^21^.

While studies employing single-cell transcriptomic (hypothesis-free) approaches in PD have recently emerged^45–50^, no prior spatial multi-omic study has examined the SNpc across PD, prodromal iLBD, and AD-associated Lewy pathology within the same analytical framework. More recently, spatial transcriptomic studies using the 10x Visium platform have been applied to the SNpc in PD and control tissue^51,52^. Our findings are consistent with this emerging literature but extend it by incorporating multiple neuropathologically defined comparator groups, including iLBD and AD with and without LBP, thereby revealing disease-context–dependent molecular alterations and enabling the identification of early, prodromal mechanisms preceding overt neurodegeneration.

Prior proteomic studies of the SNpc in PD have largely relied on bulk tissue analyses, limiting spatial resolution and disease stratification^53,54^. More recently, Griesser et al.^55^ demonstrated the feasibility of proteomic profiling from laser-capture microdissected SN tissue, providing a reference of the general nigral proteome. In contrast, our study integrates spatially resolved proteomics with spatial transcriptomics and αSyn seed amplification assays across multiple neuropathologically defined disease contexts. This approach enables transcriptomics-guided subcluster selection and localization of proteomic changes to specific SN compartments, allowing us to distinguish PD-specific molecular signatures from those associated with iLBD or AD with LB co-pathology and thereby extending prior PD–control comparisons.

Different from previous studies, we used an unbiased strategy to identify molecular targets that are impacted before LBs appear. These early-stage changes in iLBD are critical because they suggest that neurodegeneration may begin well before the appearance of overt LBP, offering an opportunity for early intervention and the development of therapeutic targets that may support disease-modification. At the molecular level, we confirmed significant alterations in the expression of key dopaminergic genes, including *SLC6A3* and *SLC18A2*, in PD cases (Fig. 2a), mirroring the loss of dopaminergic neurons. In contrast, while the expression of these marker genes was also altered in iLBD, it was less pronounced, further reinforcing the idea that iLBD represents an earlier stage of neurodegeneration.

A key finding in our study was the involvement of microglia-associated molecules in iLBD, with a lesser degree observed in PD. Our proteomic (Fig. 4e, f) and immunohistochemistry data (Fig. 5e–g) demonstrated a previously unknown upregulation of C1QC, a component of the complement system, within the SNpc, especially in the ventral tier of the SNpc, where dopaminergic neurons are particularly vulnerable in PD. In line with an active immune-associated disease process along the PD trajectory previous studies have suggested that inflammatory changes occur early in iLBD, before the onset of PD symptoms, with increased TLR-2-positive microglia in the SNpc of iLBD cases compared to PD^99,100^. Additionally, CD8-positive T-lymphocytes were found to increase in the SNpc prior to the appearance of LBP^101^. Research by Walker et al. and others demonstrated distinct inflammatory and growth factor patterns in iLBD and PD, likewise supporting early-stage neuroinflammation^102,103^. Microglial activity was also found to be heightened in iLBD in some studies, with increased IBA1-positive microglia and changes in microglial markers such as PAR2 and tyrosin-1^104^.

Previous positron emission tomography (PET) studies in individuals with isolated REM sleep behavior disorder (RBD)—a prodromal stage of PD—and in PD have yielded mixed results, with some reporting increased microglial activation and others finding no such changes^99,105–109^. Consistent with the latter, we found no evidence of microglial activation yet in midbrain tissue using in situ TSPO autoradiography across different disease stages (Supplementary Fig. 15). Nevertheless, our data suggest a microglia-dependent mechanism of synaptic remodeling that may occur independently of classical microglial activation, highlighting the importance of investigating early immune-related changes in disease progression. These findings support the potential for early-stage, immune-modulatory interventions to modify disease trajectory^100,110–112^. Although neuroinflammation has been previously explored as a therapeutic target—with variable outcomes^113^ —ongoing clinical trials (NCT05904717) continue to test this approach. Importantly, our data decouple immune-associated synaptic changes from hallmark neuropathological features: elevated C1QC levels were not associated with LBP or misfolded αSyn detected by conventional histopathological methods (Fig. 5h, i) although our results do not exclude a contributory role of earlier or distal αSyn-related processes. Together, these results suggest an early, αSyn–independent immune process and provide further rationale for targeted immunomodulation in prodromal PD. Interestingly, C1QC upregulation was not observed in PD cases, suggesting that complement activation may represent an early pathological event associated with iLBD that becomes less prominent at later disease stages. This temporal pattern may indicate a potential window during which complement-mediated mechanisms could be therapeutically targeted.

Mounting evidence suggests that SNpc dopaminergic neuron degeneration in PD begins with synaptic pathology, with the loss of synaptic connectivity preceding nerve cell loss^114^. Early studies, such as those by Sherman et al., demonstrated that PD symptoms appear when striatal denervation exceeds a critical threshold of about 50%, highlighting the importance of synaptic terminal degeneration^115,116^. These findings cumulated in the “dying back” hypothesis, where synaptic dysfunction, including presynaptic degeneration, occurs before neuronal death, with αSyn aggregates impairing synaptic function^71,117,118^. Our own results from spatial deconvolution analysis showed that high *C1QC* expression in the SNpc was not only found in proximity to microglia, but also inhibitory neurons (Fig. 5a). In accord, our confocal microscopy examination demonstrated a high abundance of the inhibitory post-synaptic protein gephyrin inside lysosomal structures of SNpc microglia (Fig. 6a, b) and additional PLA demonstrated a proximity between C1QC and gephyrin (Fig. 6d, e). Gephyrin is a scaffolding protein essential for the proper function and organization of inhibitory synapses that plays a key role in anchoring and clustering GABA A receptors and glycine receptors at the postsynaptic membrane, thereby regulating inhibitory neurotransmission^119^. Although C1QC immunoreactivity appeared to be associated with neuronal cell bodies, this likely reflects complement deposition or microglial–neuronal interactions rather than neuronal expression of C1q (Fig. 6a, b). Consistent with this mechanism, we observed a reduction in GAD67-positive fibers in iLBD and PD cases (Fig. 6f–h). In conclusion, our spatial multi-omic analysis identifies early complement activation and C1QC enrichment in the substantia nigra during the prodromal iLBD stage. Spatial transcriptomic and immunohistochemical analyses link this signal to microglia and inhibitory synaptic structures, and we observe a reduction of GABAergic terminals in both iLBD and PD. Together, these findings support a model in which early complement-mediated remodeling of inhibitory synapses precedes αSyn aggregation and dopaminergic neuron degeneration in PD. Such early synaptic alterations may represent an upstream pathogenic event and a potential therapeutic target. Our results provide a potential explanation for the previously suggested presynaptic alterations, where a disintegration of inhibitory inputs to SNpc dopaminergic neurons may lead to hyperexcitability and eventually contribute to dopaminergic neuronal death^120,121^. This interpretation aligns with current models describing alterations in GABAergic striosomes in PD^122,123^. Accordingly, recent experimental work demonstrates that chronic hyperactivation of midbrain dopaminergic neurons can itself induce selective dopaminergic neurodegeneration in mice^124^, providing additional mechanistic support for the potential vulnerability of these pathways in Parkinsonian processes. Notably, elevated C1QC levels are associated with amyloid-β plaque buildup and early synaptic loss in the cortex of AD models^68,69,125,126^ where an upregulation of C1QC has been demonstrated before plaque formation^69^. However, our study provides the evidence linking microglial activity to early synaptic dysfunction and dopaminergic degeneration in the context of PD.

Several limitations of this study warrant consideration. First, the overall cohort size is modest, reflecting the limited availability of well-characterized post-mortem human SN tissue with short post-mortem intervals, sufficient RNA quality, and detailed neuropathological staging—particularly for prodromal iLBD cases and AD cases with defined midbrain LB co-pathology. As a result, some analyses, especially those involving stratification by pathological subgroups, are statistically underpowered, and null findings should be interpreted with appropriate caution. In addition, our analyses are confined to the SNpc and therefore may not capture molecular processes occurring in cortical or limbic regions. An additional limitation is the difference in PMI and fixation time between cohorts, particularly between AD and non-AD samples due to different tissue sources. While PMI contributed to global variation in transcriptomic data (Supplementary Fig. 5), its inclusion as a covariate in regression analyses did not materially affect key biological associations. Moreover, the principal findings of this study are based on comparisons within the PD spectrum, where PMI differences were less pronounced. Nevertheless, residual confounding cannot be fully excluded.

A further limitation is the absence of a dedicated DLB cohort. Although DLB is also associated with dopamine neuron loss, it is characterized by prominent cortical LBP and clinical dementia^127^, introducing additional variables beyond the midbrain-focused disease contexts examined here. Accordingly, our conclusions regarding the relationship between synucleinopathy, C1QC activation, and nigral neurodegeneration are restricted to PD, iLBD, and AD cases with or without LB co-pathology. Extending this spatial multi-omics framework to DLB including cortical and limbic areas represents an important direction for future investigation.

Finally, although the multimodal design integrates spatial transcriptomics, spatial proteomics, histology, and biochemical assays, the cross-sectional nature of post-mortem material precludes direct inference about temporal dynamics. Consequently, the present findings should be regarded as hypothesis-generating and mechanistically informative rather than definitive, and they motivate future studies in larger, independent cohorts and across additional brain regions.

## Methods

### Neuropathological brain tissue characterization

Formalin-fixed, paraffin-embedded (FFPE) tissue sections (5 µm) of the SN were obtained from the Netherlands Brain Bank and the Parkinson’s Disease and Multiple Sclerosis Brain Bank at Imperial College London. Written informed consent for research use of samples and clinical data was obtained from all participants or their legal representatives in accordance with local institutional guidelines and the Declaration of Helsinki. Cases were selected based on a post-mortem interval of ≤52 h and a fixation time of ≤72 days, with age and sex matched across groups (Table 1). AD cases were restricted to individuals with the APOE ε4/ε3 genotype. The study included five cohorts: controls (Ctrl; LBP Braak stage 0, *n* = 6), iLBD (LBP Braak stage 1–2, *n* = 7), PD (LBP Braak stage 3–6, *n* = 9), AD (LBP Braak stage 0, Tau Braak stage 6, *n* = 5), and AD with LBP (AD + LBP; LBP Braak stage 6, Tau Braak stage 6, *n* = 5). Consecutive sections from each case were used for three experimental approaches: αSyn seed amplification assay, spatial transcriptomics, and spatial proteomics. Ethical approval for the study was obtained from the local authorities and the ethics board at LMU Munich (#21-0547). To protect participant privacy and minimize the risk of re-identification, demographic and clinical information is reported in aggregate form where appropriate.

### Quantification of pigmented neurons

Pigmented neurons were quantified from hematoxylin and eosin (H&E)-stained sections of the SN. High-resolution images were analyzed using the “Count Cells” function in ImageJ (NIH), with manual selection of neuromelanin-containing neurons based on pigmentation. Total counts were recorded per case and compared across cohorts.

### RNA quality control

Total RNA was extracted from FFPE tissue sections using the RNeasy FFPE Kit (Qiagen) according to the manufacturer’s protocol. RNA quality was assessed using the Agilent Bioanalyzer with the Pico RNA Kit, and fragment distribution was evaluated using the DV200 metric. Samples with a DV200 ≥ 30% were deemed suitable as recommended by 10X Genomics and included in the spatial transcriptomics workflow.

### Spatial transcriptomics

Spatial transcriptomics was performed using the Visium Spatial Gene Expression for FFPE CytAssist Kit (10X Genomics) according to the manufacturer’s protocol. FFPE tissue sections were deparaffinized, stained with haematoxylin and eosin (H&E), and imaged at ×10 magnification for alignment. Sections were positioned within the cassette to center the SNpc, then decrosslinked and hybridized overnight with the human probe set. After washing, probes were ligated to RNA in the tissue and digested using the CytAssist instrument. Ligated probes were transferred to spatially barcoded Visium slides, where spatial barcodes were added to preserve spatial identity. Libraries were generated via sample index PCR using the TS Index Kit A (10X Genomics) and purified with SPRIselect beads (Beckman Coulter). Quality control and fragment size analysis were conducted using the Bioanalyzer High Sensitivity DNA Kit (Agilent).

### Sequencing

Sequencing libraries were pooled at equimolar concentrations and subjected to high-throughput sequencing on the Illumina NovaSeq X+ platform using 1.5B flow cells, targeting a depth of 120 million reads per sample.

### Laser capture microdissection and protein mass spectrometry

Spatial transcriptomic data guided the anatomical localization of SNpc and fiber clusters for targeted isolation by laser capture microdissection (LCM; Leica LMD 7000). FFPE sections were mounted on PEN membrane slides (Thermo Fisher Scientific), deparaffinized, and stained with hematoxylin and eosin (H&E) to aid regional annotation. Per cluster, 300,000 µm² were microdissected and stored at −80 °C until protein extraction. Samples were lysed in 18 µL of 100 mM ammonium bicarbonate (ABC) containing 0.2% dodecyl-β-D-maltoside DDM at 95 °C for 60 min, followed by denaturation with 3 µL of 80% acetonitrile (ACN) at 75 °C for 30 min. Proteins were digested overnight at 37 °C with LysC (150 ng) and trypsin (300 ng) in 50 mM ABC. Digestion was quenched using 2 µL of 8% formic acid, and peptides were dried via SpeedVac (Thermo Fisher Scientific). Desalting was performed on C18 StageTips preconditioned with methanol and 80% ACN/0.1% FA, with final elution in 80% ACN/0.1% FA. Eluted peptides were dried and resuspended in 10 µL of 0.1% FA. For LC-MS/MS analysis, 6 µL of peptide solution was separated on a self-packed C18 analytical column (15 cm × 75 µm, 1.9 µm, Dr. Maisch GmbH) using a nanoElute HPLC coupled to a TimsTOF Pro mass spectrometer (Bruker) with a CaptiveSpray source. Peptides were resolved by a binary gradient (2–24% B over 62 min; 10 min to 35% B; 5 min to 85% B; solvent B: 80% ACN/0.1% FA) at 50 °C. Data were acquired in positive ion mode using data-independent acquisition parallel accumulation–serial fragmentation (DIA-PASEF) with a TIMS ramp time of 100 ms. Each scan cycle included one full TIMS MS scan and 26 DIA windows (27 m/z width) covering 350–1002 m/z, with a 1.4 s cycle time. Peptide identification was performed using DIA-NN (v1.8.2) with a 0.01 false discovery rate (FDR), using a library-free search against an in silico spectral library generated from the mouse reference proteome. Search parameters included up to two missed cleavages, peptide lengths of 7–30 residues, precursor charges of 1–4, and variable modifications (UniMod:35, UniMod:1). Match-between-runs and retention time normalization were enabled.

### αSyn recombinant substrate preparation

αSyn was expressed and purified as previously described^128^. Briefly, DNA sequences coding for human wild-type αSyn sequence (Accession No. NM_000345.3) amino acid residues 1–140 were amplified and ligated into the pET24 vector with an N-terminal His-tag (EMD Biosciences) and the sequences were confirmed. The plasmids were transformed into BL21(DE3) *E. coli* (EMD Biosciences), the bacteria streaked onto kanamycin-selective agar (50 µg/mL) and incubated overnight at 37 °C. A single colony was cultured in 5 mL LB medium containing kanamycin with shaking (250 rpm, 37 °C, 4–5 h), followed by expansion into 1 L LB supplemented with kanamycin and the Overnight Express Auto-Induction System (Merck-Millipore). Cultures were incubated at 37 °C, 200 rpm overnight, and aliquoted into 250 mL fractions. Cells were pelleted (3200 × *g*, 10 min, 4 °C) and resuspended in 25 mL osmotic shock buffer (40% sucrose, 2 mM EDTA, 30 mM Tris, pH 7.2). Following 10 min incubation at room temperature with gentle agitation, cells were pelleted (9000 × *g*, 20 min, 20 °C), resuspended in 10 mL ice-cold Milli-Q water, and treated with 20 µL saturated MgCl₂. Lysates were incubated on ice with gentle rocking and centrifuged (9000 × *g*, 30 min, 4 °C). The supernatant was acidified to pH 3.5 using 1 M HCl, stirred for 10 min at room temperature, and centrifuged again (9000 × *g*, 30 min, 4 °C). The resulting supernatant was neutralized to pH 7.5 with 1 M NaOH, filtered (0.22 µm), and subjected to Ni-NTA affinity chromatography (Cytiva) on an NGC chromatography system (Bio-Rad). Columns were equilibrated with 20 mM Tris (pH 7.5), washed with 50 mM imidazole, and eluted with a linear imidazole gradient (150–375 mM). Eluates were further purified by anion exchange chromatography (Q-HP, Cytiva), using a linear NaCl gradient (100–350 mM) in 20 mM Tris (pH 7.5). Pooled fractions were filtered (0.22 µm), dialyzed against Milli-Q water at 4 °C overnight in a 3.5-kDa MWCO membrane (Thermo-Scientific), followed by a second dialysis step in fresh Milli-Q water for 4 h. Protein concentration was determined by UV spectrophotometry (*ε* = 0.36 mg⁻¹ mL cm⁻¹ at 280 nm), lyophilized (6 h), and reconstituted in 500 µL of 40 mM phosphate buffer (pH 8.0, Sigma). Aliquots were stored at −80 °C.

### Processing of FFPE tissue for real-time quaking-induced conversion (RT-QuIC) analysis

FFPE midbrain sections were processed for RT-QuIC analysis following a modified version of the protocol by Hepker et al.^129^. Tissue was scraped from slides using a sterile razor blade and wetted with 3–4 drops of 100% ethanol. Samples were transferred to 1.5 mL tubes, resuspended in 950 µL of xylene, and vortexed for 5 min. The suspension was incubated at 25 °C with agitation (1500 rpm, ThermoMixer) for 5 min, followed by paraffin melting at 62 °C for 10 min. Samples were centrifuged (13,000 × *g*, 5 min, 25 °C), the supernatant discarded, and the pellet washed twice with 950 µL of 100% ethanol. Each wash included vortexing, incubation (25 °C, 5 min, 1,500 rpm), and centrifugation (13,000 × *g*, 5 min, 25 °C). Residual ethanol was evaporated by leaving tubes open for 15 min under a laminar flow hood at room temperature. Dry pellets were homogenized with 950 µL of TSB buffer (500 mM Tris, 0.02% SDS, pH 8.0) using a motorized pestle (Kimble Pellet Pestle). Homogenates underwent three freeze-thaw cycles (5 min in dry ice, 5 min at 40 °C), followed by incubation at 25 °C (10 min, 1500 rpm) and centrifugation (13,000 × *g*, 5 min, 25 °C). Supernatants (900 µL) were discarded, replaced with fresh TSB buffer (900 µL), mixed again, and centrifuged (13,000 × *g*, 10 min, 25 °C). Residual supernatant was removed, leaving ~30 µL per sample. Tubes were incubated at 90 °C (20 min, 1500 rpm), followed by 60 °C for 90 min. After brief centrifugation, samples were flash-frozen on dry ice, then sonicated at 50% amplitude for 1 min, alternating with freezing steps, for a total of four freeze-sonicate cycles. Final centrifugation (2000 × *g*, 10 min, 4 °C) was performed to collect the clarified supernatant for RT-QuIC seeding assays.

### αSyn RT-QuIC analysis

RT-QuIC analysis was performed in a blinded manner, according to the protocol for brain homogenates outlined by Bentivenga et al.^79^. Six 0.8-mm silica beads (OPS Diagnostics) were pre-loaded into each well of black 96-well plates with clear bottoms (Nalgene Nunc International). Prior to assay setup, samples were thawed and vortexed for 10 s. To each well, 2 µL of the sample was added to 98 µL of a reaction mixture containing 40 mM phosphate buffer (pH 8.0), 170 mM NaCl, 10 µM thioflavin-T (Sigma), and 0.1 g/L filtered recombinant αSyn (prepared using 100-kDa Amicon centrifugal filters, Merck Millipore). The plates were sealed with Nalgene Nunc International plate sealer film and incubated at 42 °C in a FLUOstar Omega plate reader (BMG Labtech), with cycles consisting of 1 min of double orbital shaking at 400 rpm, followed by 1 min of rest. Fluorescence was measured every 45 min using excitation/emission filters of 450/480 nm over 30 h. Each sample and control was analyzed in quadruplicate. A positive result was defined as three or more out of four replicates exceeding a fluorescence threshold set at 30% of the median Imax value of positive controls. To minimize false positives, samples with seeding activity in only one or two replicates during the initial run were reanalyzed in triplicate runs. A sample was considered positive if at least four of the 12 total replicates exceeded the threshold. For quantification, the area under the amplification curve was calculated, and the median value from the four runs per sample was used for comparisons. Quality control of recombinant αSyn was performed before use, and positive and negative controls were included on each plate. Positive controls were brain samples from patients with probable or confirmed dementia with Lewy bodies (DLB) or PD, which consistently produced four positive replicates during preliminary screening. A valid experiment required at least three positive replicates per plate for the positive controls.

### Data processing and analysis

Spatial transcriptomic data in FASTQ format were aligned to the GRCh38 genome with Spaceranger (10X Genomics, version 3.1.1). Of the 54,580 probes included in the FFPE Visium v2 assay, 8652 were removed as recommended by the 10X QC department (personal communication), resulting in coverage data for 18,561 genes, while removing 861 from the whole probe-based panel. Robust cell type deconvolution (RCTD) from the Spacexr package^130^ was run on the raw probe-barcode matrix in full mode, giving different weights for known midbrain cell types per Visium spot. We used the data from Kamath et al.^45^ as a reference object, as it provided a comprehensive atlas of the human SN and resulted in good quality control metrics. The Bayesian Analytics for Spatial Segmentation (BASS) package was used for assigning consensus spatial clusters across samples by modeling the tissue regional scale under a Bayesian hierarchical modeling framework^60^. Cluster assignments were visually inspected and annotated based on the known midbrain histology. The Markov Affinity-based Graph Imputation of Cells (MAGIC) framework was then used on the spot data only for visual enhancement of spatial feature plots^60^, while raw (non-imputed) counts were used for analysis. Using the information from above, a Seurat object was created for further downstream analyses (version 5.1.0), and gene expression was log-normalized and scaled^131^. For differential expression analyses, we first checked for batch effects using multidimensional scaling plots (Supplementary Fig. 5). Post-mortem interval (PMI) was not included as a covariate because it was strongly correlated with kit batch (*p* = 1 × 10⁻⁴), reflecting the study design in which samples with different PMI ranges were processed in distinct batches. Including both variables introduced collinearity without improving model performance, as variance inflation diagnostics indicated shared explanatory variance and effect size estimates were unchanged when PMI was added. Accordingly, we removed the effect of sex and Visium kit lot number while testing for differentially expressed genes with the edgeR framework, which uses negative binomial models and Quasi-Likelihood F-Tests under the hood. All DE analyses were conducted in pseudobulk fashion by grouping spot gene expression from BASS-assigned clusters. To test for differential protein expression, the DEqMS framework was employed^65^, using the limma package while simultaneously correcting *p*-values for the number of peptides used for protein quantification, a frequent confounder in proteomic studies. All analyses were carried out in R (version 4.4.1) on a workstation running Arch Linux, making heavy use of the tidyverse and ggplot2 packages^132^.

### Immunohistochemistry

Paraffin-embedded tissue sections (5 µm) were mounted on uncoated glass slides and processed for immunohistochemical analysis. Slides were deparaffinized in xylene and rehydrated through a graded ethanol series, followed by a brief rinse in phosphate-buffered saline (PBS). Heat-induced epitope retrieval was performed in 10 mM sodium citrate buffer (pH 6.0) at 95 °C for 1 h. After cooling to room temperature and additional PBS washes, sections were blocked for 1 h in a solution containing 5% donkey and goat serum, 1% bovine serum albumin (BSA), and 0.3% Triton X-100 in PBS. Primary antibodies were applied in blocking solution, and slides were incubated at 4 °C for 48 h. Following extensive PBS washes, sections were incubated with fluorophore-conjugated secondary antibodies for 1 h at room temperature, protected from light. After final PBS washes, slides were air-dried and coverslipped with DAPI-containing fluorescence mounting medium.

### Primary antibodies

The following primary antibodies were used in this study: TH (sheep, 1:500, Invitrogen, Cat. No. PA1-4679), C1QC (rabbit, 1:100, Life Technologies, Cat. No. PA5-106648), gephyrin (mouse, 1:200, Synaptic Systems, Cat. No. 147011), GAD67 (mouse, 1:100, Sigma, Cat. No. MAB5406B), IBA1 (guinea pig, 1:500, Synaptic Systems, Cat. No. 234308), CD68 (rat, 1:500, BioRad, Cat. No. MCA1957), CD18 (rabbit, 1:100, Abcam, Cat. No. AB131044), and NPY (rabbit, 1:100, Invitrogen, Cat. No. PA5-85762).

### Secondary antibodies

The following secondary antibodies were used in this study: donkey anti-rabbit Alexa Fluor 647 (1:500, Invitrogen, Cat. No. A31573), donkey anti-rabbit Alexa Fluor 488 (1:500, Invitrogen, Cat. No. A21206), donkey anti-sheep Alexa Fluor 555 (1:500, Invitrogen, Cat. No. A21436), donkey anti-sheep Alexa Fluor 488 (1:500, Invitrogen, Cat. No. A11015), donkey anti-rat Alexa Fluor 647 (1:500, Invitrogen, Cat. No. A48272), donkey anti-mouse Alexa Fluor 594 (1:500, Invitrogen, Cat. No. A21203), goat anti-guinea pig Alexa Fluor 488 (1:500, Invitrogen, Cat. No. A11073), and streptavidin Alexa Fluor 647 (1:500, Invitrogen, Cat. No. S32357).

### Proximity ligation assay

For proximity ligation assay (PLA) detecting gephyrin-C1QC interaction, the Naveniflex (NR.MR.100 Red) kit was used according to the manufacturer’s instructions. Briefly, sections were deparaffinized and de-crosslinked as described above. Tissue sections were then blocked using the blocking solution provided in the kit and incubated at 37 °C in a humidity chamber. Primary antibodies were applied, and slides were incubated overnight at 4 °C in a humidity chamber. The next day, primary antibodies were removed, and PLA probes specific to the host species of the two primary antibodies used for colocalization analysis were added. Samples were then incubated for 60 min at 37 °C. Next, the two sequential enzyme reactions provided by the kit were performed, followed by applying the post-block and detection solution. To visualize the PLA signal within the TH signal, a secondary antibody specific to the TH primary antibody was applied at this stage and incubated for 1 h at room temperature, protected from light. Finally, the slides were washed, air-dried, and mounted with a DAPI-containing fluorescent mounting medium.

To detect oligomeric αSyn, a PLA was performed as previously described^59^, with modifications. PLA probes were generated by conjugating both the PLUS and MINUS oligonucleotides (DUO92009 and DUO92010, Sigma-Aldrich) to the same monoclonal antibody targeting monomeric αSyn (clone 211, mouse IgG, ab206675 abcam), using the Duolink® Probemaker kit (Sigma-Aldrich) according to the manufacturer’s instructions. Antibody-probe conjugation was carried out overnight at room temperature in conjugation buffer, followed by incubation with stop solution for 30 min at room temperature. Tissue sections were deparaffinized, antigen-retrieved, and subjected to PLA as follows: sections were blocked for 1 h at 37 °C with the blocking solution provided in the Duolink In Situ Fluorescence kit (DUO92008, Sigma-Aldrich), then incubated with the PLA probes and a sheep anti-tyrosine hydroxylase (TH) antibody for 1 h at 37 °C and subsequently overnight at 4 °C. Following washes, slides were incubated in ligation buffer containing ligase (1 h, 37 °C), washed again, and then incubated with the amplification mix containing polymerase (2 h, 37 °C). After washes, sections were counterstained using a secondary antibody (donkey anti-sheep), washed and mounted using a DAPI mounting medium.

### Confocal microscopy

Slides were imaged using the Leica Stellaris 5 confocal microscope. The region of interest was identified, and sections were imaged using a z-stack depth of 3 µm with five optical sections. Laser settings, z-stack depth, and acquisition parameters were consistent across all images for a given staining.

### Image analysis Image J

A maximum intensity projection of the z-stack was generated to flatten the image for subsequent analysis. To reduce background noise, images were thresholded uniformly across all samples. Signal quantification was performed using the image analysis tool, specifically measuring the area of the signal. To delineate lysosomes specific to microglia, a mask was applied to the IBA1-positive microglia signal, which was then used to isolate the CD68 signal. A secondary mask, generated from the colocalized IBA1 and CD68 signals, was subsequently applied to the Gephyrin channel to extract Gephyrin-specific signals within the CD68-positive lysosomal compartments. Quantification of the resulting signal was performed, and data were normalized to the area of the CD68 signal.

### Image analysis IMARIS

IMARIS software was used for 3D image reconstruction. Surface rendering was applied to each channel, ensuring that creation parameters (e.g., thresholding and signal filters) were consistent across all images. The colocalization feature was then used to identify and quantify channel overlap.

### Immunohistochemical staining of post-mortem tissue

Immunohistochemistry, including counterstaining with hematoxylin was performed on 5 µm thick paraffin sections using a Ventana BenchMark ULTRA (Roche) according to the manufacturer’s instructions. For automated αSyn staining, Abcam clone 42 (ab280382) was used. The pretreatment included incubation in 80% formic acid for 15 min and boiling in CC1 mild buffer (Roche). After incubation with the primary antibody for 32 min, DAB/peroxidase-based staining was done with the ultraView detection system (Roche). Immunohistochemical sections were then scanned with a Zeiss Axio Scan.Z1 using a 20× objective.

### Generation of α-Syn PFFs

Recombinant α-Syn proteins were purified, and in vitro fibrils were assembled according to established protocols^1,2^. Full-length wild-type mouse α-Syn at a concentration of 5 mg/ml was used for fibril assembly. The assembly reactions were performed under continuous agitation using an Eppendorf orbital mixer set to 1000 rpm at 37 °C. After a 7-day incubation period, the resulting PFFs were collected for subsequent experiments. After generating PFFs, they were aliquoted into single-use portions at room temperature. Prior to use in surgeries, two quality control assays—Thioflavin T (ThT) assay and sedimentation assay—were performed to confirm that α-Syn had aggregated into the desired conformation. For the ThT assay, a fresh 1 mM ThT stock solution was prepared in deionized water (dH₂O), filtered through a 0.2 μm syringe filter, and diluted in PBS (pH 7.4) to a final concentration of 25 μM in each well. α-Syn aliquots (monomer or aggregate, 5 mg/ml) were thawed to room temperature. Aggregates (10 μM) or monomers (100 μM) were added to the wells, and the plate was sealed and incubated at 37 °C with shaking at 600 rpm for 30 min. ThT fluorescence was measured using a microplate reader (CLARIOstar Plus (BMG Labtech); excitation: 450 nm, emission: 485 nm) at 37 °C. For sedimentation assay, PFFs (2 μL, 5 mg/mL) were diluted in 20 μL of PBS and subjected to ultracentrifugation (Beckman Optima MAX-XP) at 100,000 × g for 30 min at 25 °C. After centrifugation, the supernatant was removed and mixed with 4× Laemmli buffer. The pellet was resuspended in 20 μL of PBS, mixed with 4× Laemmli buffer, and boiled at 95 °C for 5 minutes. Equal volumes of the supernatant and pellet fractions were resolved on a 15% polyacrylamide gel, and protein bands were visualized using Coomassie Brilliant Blue staining.

### Animals

All animal experiments were performed in accordance with the guidelines of the animal committee at LMU Munich and were approved by the local authorities (Regierung von Oberbayern) under protocol number ROB-55.2-2532.Vet_02-20-30. Wild-type C57BL/6J mice were obtained from Jackson Labs, and αSyn transgenic mice overexpressing human αSyn under the PDGFB promoter were maintained on a C57BL/6J background. Male and female mice aged 2–3 months were used for experiments. Animals were housed under specific pathogen-free conditions with ad libitum access to food and water under a 12 h light/12 h dark cycle. Mice expressing αSyn under the human Platelet-Derived Growth Factor (PDGF-αSynTg) were purchased from Jackson Laboratory (B6;D2-Tg(PDGFB-SNCA)4Ema/RoriJ; Strain No.: #038775).

### Stereotaxic Injection of PFF into mouse brain

Harvested PFF aliquots were sonicated using a probe sonicator (Bandelin, Sonoplus Mini 20) prior to intracerebral administration. The sonication protocol consisted of four cycles at 30% amplitude for 15 s each (1-s pulse on, 2-s pulse off). During the experiment, sonicated PFFs were maintained at room temperature to prevent dissociation into monomers. Mice aged 2–3 months were anesthetized via intraperitoneal injection of an MMF solution (0.5 mg/kg Medetomidin, 5 mg/kg Midazolam, and 0.05 mg/kg Fentanyl). Stereotaxic injections of recombinant α-Syn fibrils (5 mg/mL) were performed bilaterally into the dorsal striatum using a Neurostar glass-capillary Nanoinjector. Control groups received sterile PBS injections. For the dorsal striatum, the glass pipette was positioned at the following coordinates relative to Bregma: anterior-posterior (AP) + 0.2 mm, mediolateral (ML) ±2.0 mm, and dorsoventral (DV) −3.8 mm beneath the dura mater. A total of 2 μl was injected per site at a flow rate of 200 nl/min. The pipette was held in place for 5 min after the injection to minimize reflux. After surgery, mice were given a subcutaneous injection of an AF solution (2.5 mg/kg Antipamezol and 0.5 mg/kg Flumazenil) to reverse anesthesia. Animals were monitored routinely during recovery. At 3 months post-injection (mpi), mice were sacrificed via ketamine/xylazine overdose for further analysis.

### Acute slice preparation

Coronal brain slices containing the substantia nigra pars compacta (SNc) were prepared from male C57BL/6J mice (postnatal day ~100, PN100). Mice were deeply anesthetized with isoflurane and transcardially perfused with 15 mL ice-cold, oxygenated glycerol-based artificial cerebrospinal fluid (GaCSF). The GaCSF was composed of (in mM): 250 glycerol, 2.5 KCl, 2 MgCl₂, 2 CaCl₂, 1.2 NaH₂PO₄, 10 HEPES, 26 NaHCO₃, and 5 glucose; pH was adjusted to 7.2 with 2.5 M NaOH, and osmolarity was ~310 mOsm. Following perfusion, the brain was rapidly removed by decapitation, and the frontal cortex and cerebellum were trimmed within 1 min to isolate a midbrain tissue block. Coronal sections (270 μm) were cut using a vibratome (Campden Instruments 7000smz) in ice-cold, carbogenated (95% O₂/5% CO₂) GaCSF. Slices were then transferred to normal aCSF for recovery. The composition of aCSF was (in mM): 125 NaCl, 2.5 KCl, 2 MgCl₂, 2 CaCl₂, 1.2 NaH₂PO₄, 10 HEPES, 26 NaHCO₃, and 5 glucose, adjusted to pH 7.2 with 2.5 M NaOH, with final osmolarity ~310 mOsm. Slices were incubated at 35 °C for 30 min in a water bath before further use. Human α-synuclein seeds were prepared from SAA-positive frontal cortex homogenate (10⁻² dilution) derived from a 74-year-old female patient clinically diagnosed with Lewy body dementia (LBD). For seed fragmentation, 100 μL of lysate was loaded into a 500 μL microcentrifuge tube and sonicated using a probe sonicator (Bandelin, Sonoplus mini-20), with 30% amplitude; 15 s × 4 repetitions; 1 s on, 2 s off, ensuring the probe tip remained suspended in the center of the liquid without contacting the tube wall. A minimum 30-second interval was maintained between sonication cycles to minimize thermal effects. Following sonication, 100 μL of fragmented α-synuclein seeds were added directly to the slice incubation chamber containing oxygenated aCSF, and slices were incubated for 4 h at 32 °C. Control slices were treated with the vehicle used during human tissue homogenization (0.5 M phosphate buffer, pH 8.0; 2 M NaCl; 0.05% SDS in 40 mM phosphate buffer). After incubation, slices were fixed in 4% paraformaldehyde (PFA) for 20 min at room temperature and stored in phosphate-buffered saline (PBS) at 4 °C until immunofluorescence staining.

### Immunohistochemistry

Mouse brains were collected following transcardial perfusion with PBS and 4% paraformaldehyde (PFA). Brains were post-fixed overnight in 4% PFA at 4 °C. After post-fixation, PFA was discarded, and the brains were stored in PBS at 4 °C. Tissues were sectioned using a vibratome (Leica VT1200S) into 50 μm-thick slices, which were stored in an antifreeze solution (30% ethylene glycol, 30% glycerol, and 40% PBS) at −20 °C until further processing. Free-floating brain sections (50 μm) were washed in PBS before being subjected to antigen retrieval. Antigen retrieval was performed by incubating sections in Tris-Citrate buffer (10 mM sodium citrate, 0.05% Tween-20, pH 6.0) at 95 °C in a water bath for 20 min. After cooling, sections were washed in PBS and permeabilized with 0.3% Triton X-100 in PBS for 4 h at room temperature on a shaker. Following permeabilization, sections were washed and incubated in blocking solution (2% bovine serum albumin, 5% donkey serum, and 0.3% Triton X-100 in PBS) for 1 h at room temperature. The blocking solution was replaced with primary antibody diluted in the same buffer, and sections were incubated overnight at 4 °C on a shaker. The next day, sections were washed three times in PBS (10 min per wash) and incubated with fluorophore-conjugated, species-specific secondary antibodies prepared in the diluted blocking solution. Secondary antibody incubation was conducted for 2 h at room temperature in the dark. Following incubation, sections were washed thoroughly in PBS, mounted onto frosted microscope slides, and cover-slipped using DAPI fluorescent mounting medium for confocal imaging. Confocal images were acquired using a Zeiss LSM 900 or Leica Stellaris 5 microscope with a z-stack thickness of 25 μm. Image processing and signal enhancement were performed using FIJI software (version 1.54f). To quantify signals of specific markers, such as IDE and pS129, FIJI software was used for data analysis following a standardized protocol. Z-stack images were saved in TIFF RGB format and converted to 8-bit grayscale. Images were processed via Z-projection at maximum intensity, and brightness curves were adjusted to ensure high-intensity regions did not obscure other areas of interest. Uniform trimming and filtering parameters were applied consistently across all images. Thresholding was performed using the default method to convert images into binary format, highlighting regions of interest. Using FIJI’s “Set Measurements” tool, the “area” and “area fraction” options were selected, and the “Measure” function was used to calculate the percentage area (% Area), representing the density of signal for the targeted marker.

### Autoradiography

[^18^F]DPA-714 autoradiography from formalin-fixed and paraffin-embedded tissue blocks from 30 cases with different neurodegenerative diseases and controls was processed. Deparaffinized sections were incubated for 60 min (135.1 µCi/ml after dilution to a volume of 60 ml with phosphate buffered saline solution, pH 7.4, molecular activity 3961 GBq/µmol at the end of synthesis). Washing was performed by PBS for 1 min, 70% ethanol/PBS for 2 min, 30% ethanol/PBS for 1 min and PBS for 1 min to remove unbound radiotracer. After drying at room temperature for 60 min, the sections were placed on Fujifilm BAS cassette2 2025 imaging plates. The plates were exposed for approximately 15 h and then scanned at 25.0 µm resolution with the Elysia-raytest equipment (CR-35 BIO, Dürr Medical, Bietigheim-Bissingen, Germany). Resulting images were analyzed with a dedicated software (AIDA image analysis, V4.50, Elysia-raytest, Straubenhardt, Germany). To investigate TSPO-related [^18^F]DPA-714 signal in the midbrain, a region of interest was drawn in the SNpc on each sample. A TSPO-negative region in the white matter fiber tract was determined as a reference region. Ratios between target regions and the reference region were calculated and correlated with the AUC extracted from SAA data.

### Reporting summary

Further information on research design is available in the Nature Portfolio Reporting Summary linked to this article.

## Supplementary information

Supplementary Information

Reporting Summary

Transparent Peer Review file

## Source data

Source Data

## Data Availability

Spatial transcriptomics data generated in this study have been deposited in the NCBI Sequence Read Archive (SRA) under accession code PRJNA1357890. Raw data accessions (fastq files) range from SRX31009153 to SRX31009184 and can be found under the associated BioProject accession ID PRJNA1357890. Proteomics data generated in this study have been deposited in the ProteomeXchange Consortium via the PRIDE partner repository under accession code PXD062998. Source data are provided with this paper.

## Code availability

The code necessary to reproduce the findings is available at https://github.com/fstrueb/spatial_nigra.
