# Supplementary material for: Spatial multi-omics identifies early synaptic pruning and context-specific dopaminergic vulnerability in synucleinopathies
Source: Nat Commun. 2026 Jul 21;17:6976. doi: 10.1038/s41467-026-74961-6 (PMC13392254; doi:10.1038/s41467-026-74961-6)
Supplement: Supplementary file 1 — Supplementary Information [file 41467_2026_74961_MOESM1_ESM.docx]

**Supplemental Information:**

Spatial Multi-Omics Identifies Early Synaptic Pruning and Context-Specific Dopaminergic Vulnerability in Synucleinopathies.

**
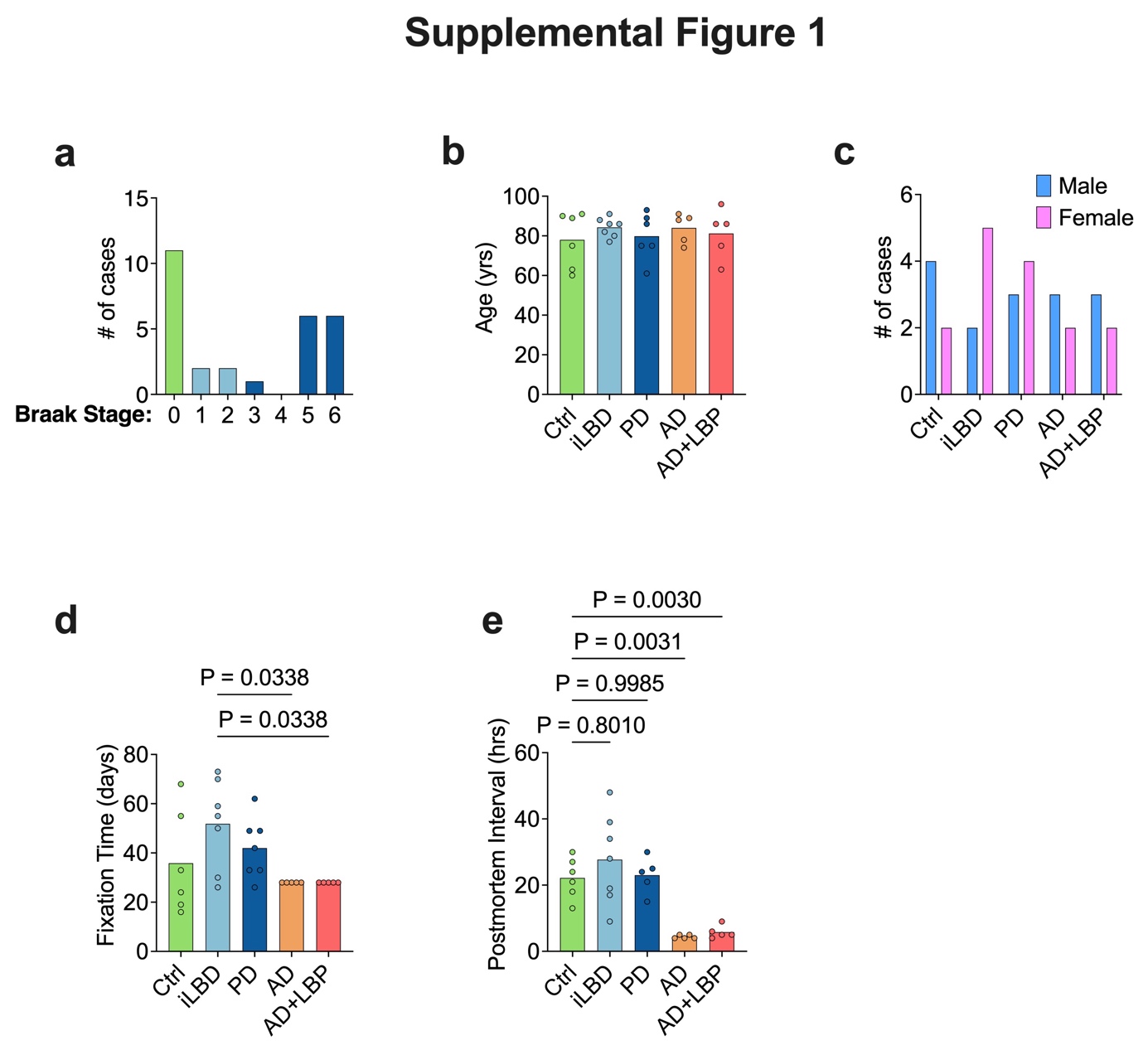
**

**Supplemental Figure 1:** a, Bar graph showing the number of cases analysed per LB Braak stage. b, Bar graph showing donor age (years) across the five cohorts. c, Bar graph representing the distribution of male and female donors in each cohort. d, Bar graph depicting tissue fixation time (days) across cohorts. e, Bar graph illustrating post-mortem interval (hours) across cohorts. Data in b, d, and e are presented as mean, and individual points represent biologically independent donor samples (Ctrl, n = 6; iLBD, n = 7; PD, n = 9; AD, n = 5; AD+LBP, n = 5). Statistical significance in b, d, and e was assessed using Welch’s ANOVA. Sex distribution in c was assessed using Fisher’s exact test. Source data are provided as a Source Data file.


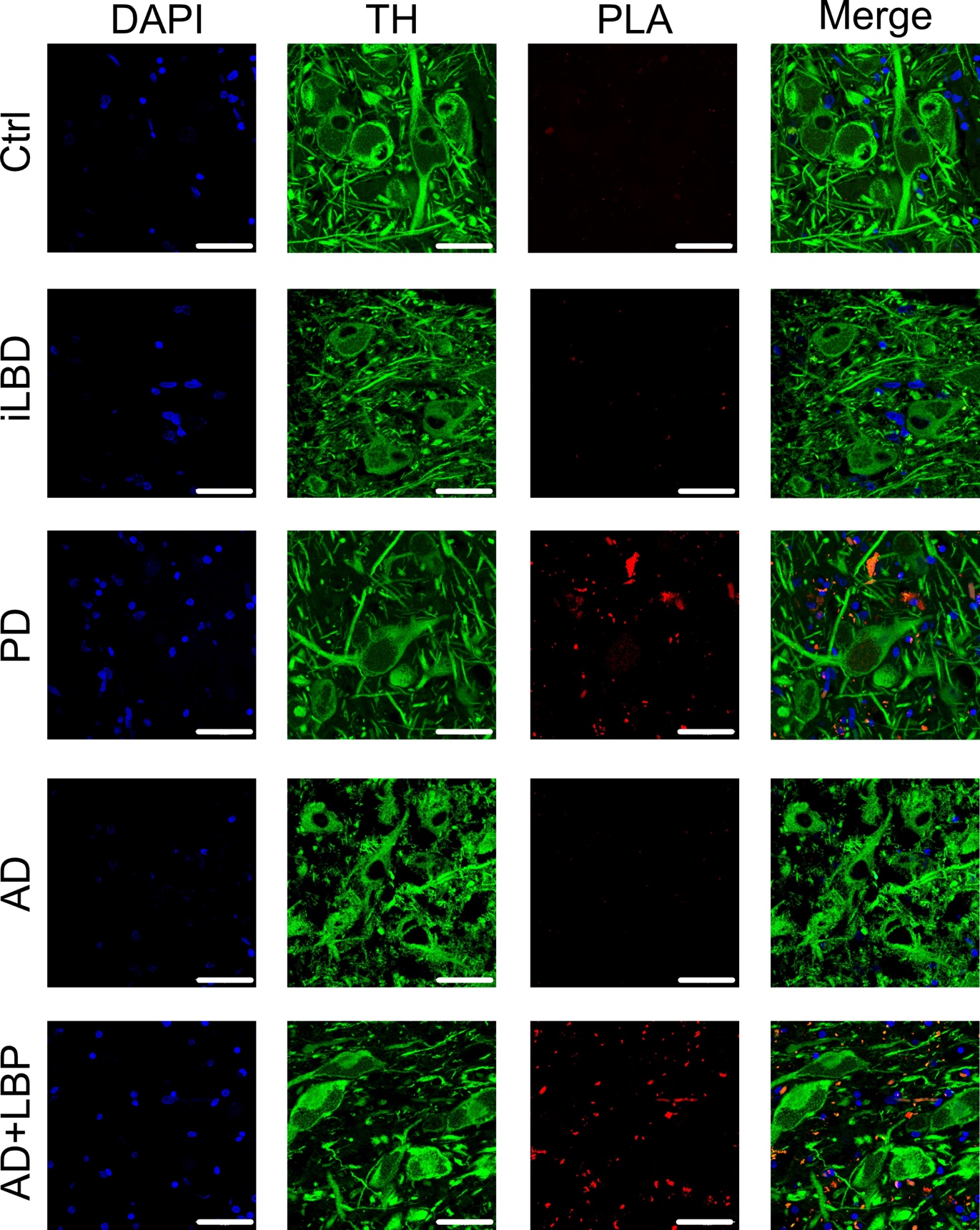


**Supplemental Figure 2:** Representative photomicrographs showing tyrosine hydroxylase (TH; green) and αSyn proximity ligation assay (αSyn-PLA) labeling in tissue sections from all five cohorts. αSyn-PLA signal was prominent in PD and AD+LBP, consistent with the presence of αSyn oligomers > 40 nm. Scale bar = 50 μm.


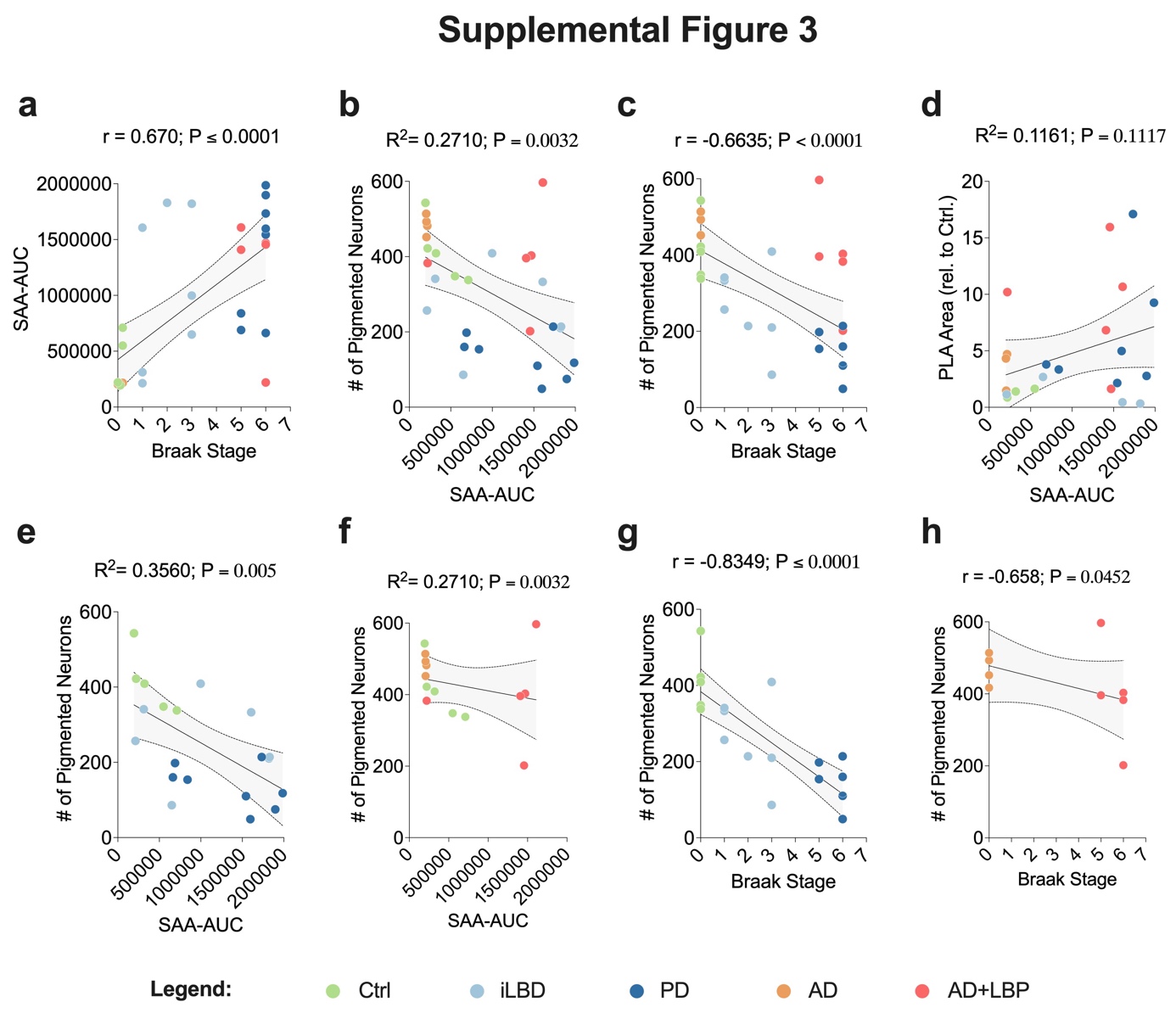


**Supplemental Figure 3:** Correlation between mean seed amplification assay area under the curve (SAA–AUC), Braak stage, and the number of pigmented neurons per tissue section. Data points represent biologically independent donor samples and are color-coded by cohort: green, Ctrl; light blue, iLBD; dark blue, PD; orange, AD; red, AD+LBP. Panels a–d show correlations for the entire dataset. Panels e and g show correlations between pigmented neurons per section and either SAA–AUC (e) or Braak stage (g) for the Ctrl, iLBD, and PD groups. Panels f and h show the same analyses for the AD and AD+LBP groups. Pearson correlation analysis and coefficients of determination (R²) were calculated for panels b, d, e, and f. Spearman rank correlation analysis and correlation coefficients (r) were calculated for panels a, c, g, and h, and for all analyses involving non-parametric data (Braak stage). Source data are provided as a Source Data file.


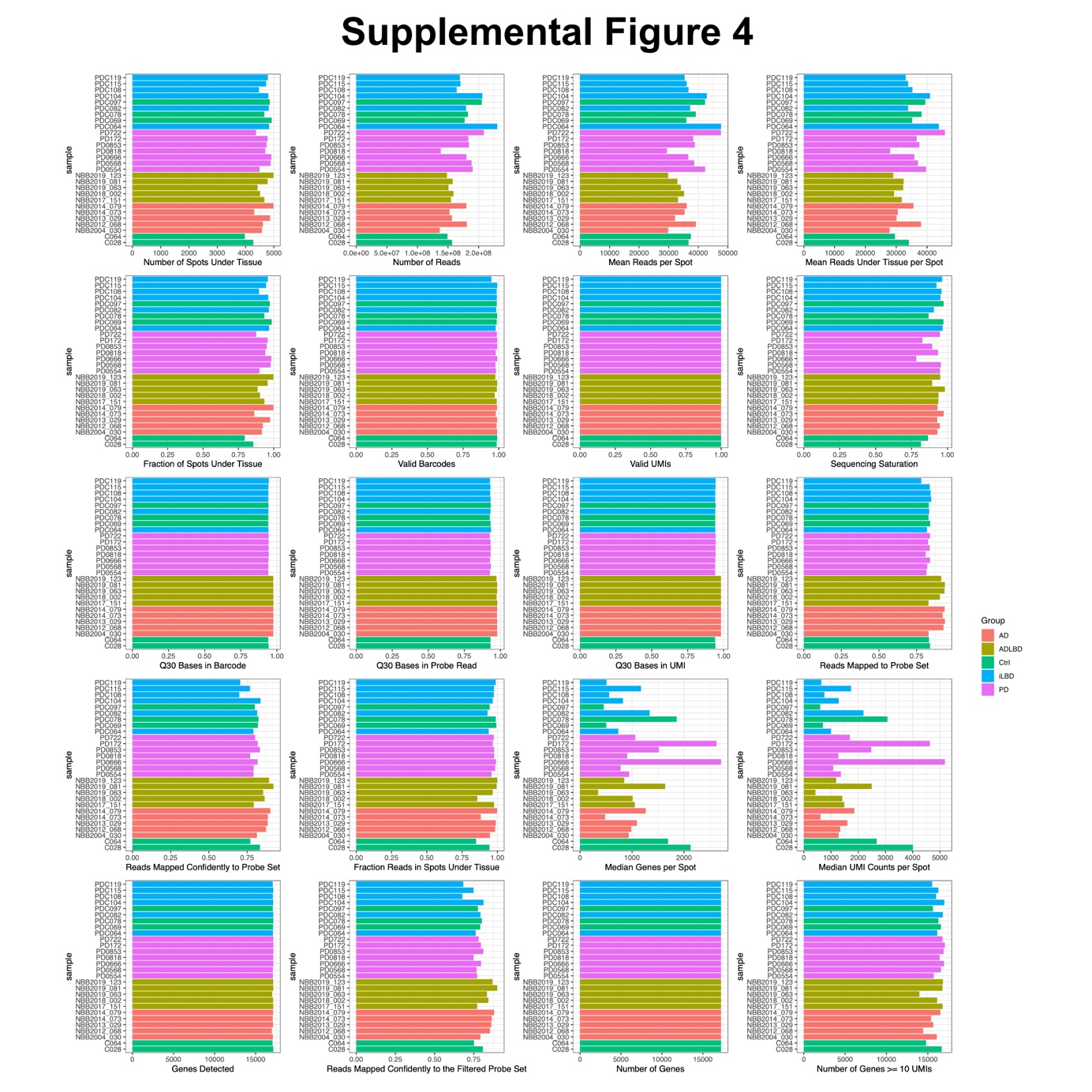


**Supplemental Figure 4:** Summary quality control metrics for all spatial transcriptomic samples, including number of spots under tissue, total reads, mean reads per spot, mean reads under tissue per spot, fraction of spots under tissue, number of valid barcodes, valid UMIs, sequencing saturation, Q30 bases in barcode, probe read and UMI, reads mapped to the probe set, reads mapped confidently to the probe set, fraction of reads in spots under tissue, median genes per spot, median UMI counts per spot, total genes detected, reads mapped confidently to the filtered probe set, number of genes, and number of genes detected with >10 UMIs. Individual points represent biologically independent donor samples. Disease groups are indicated in the figure legend.


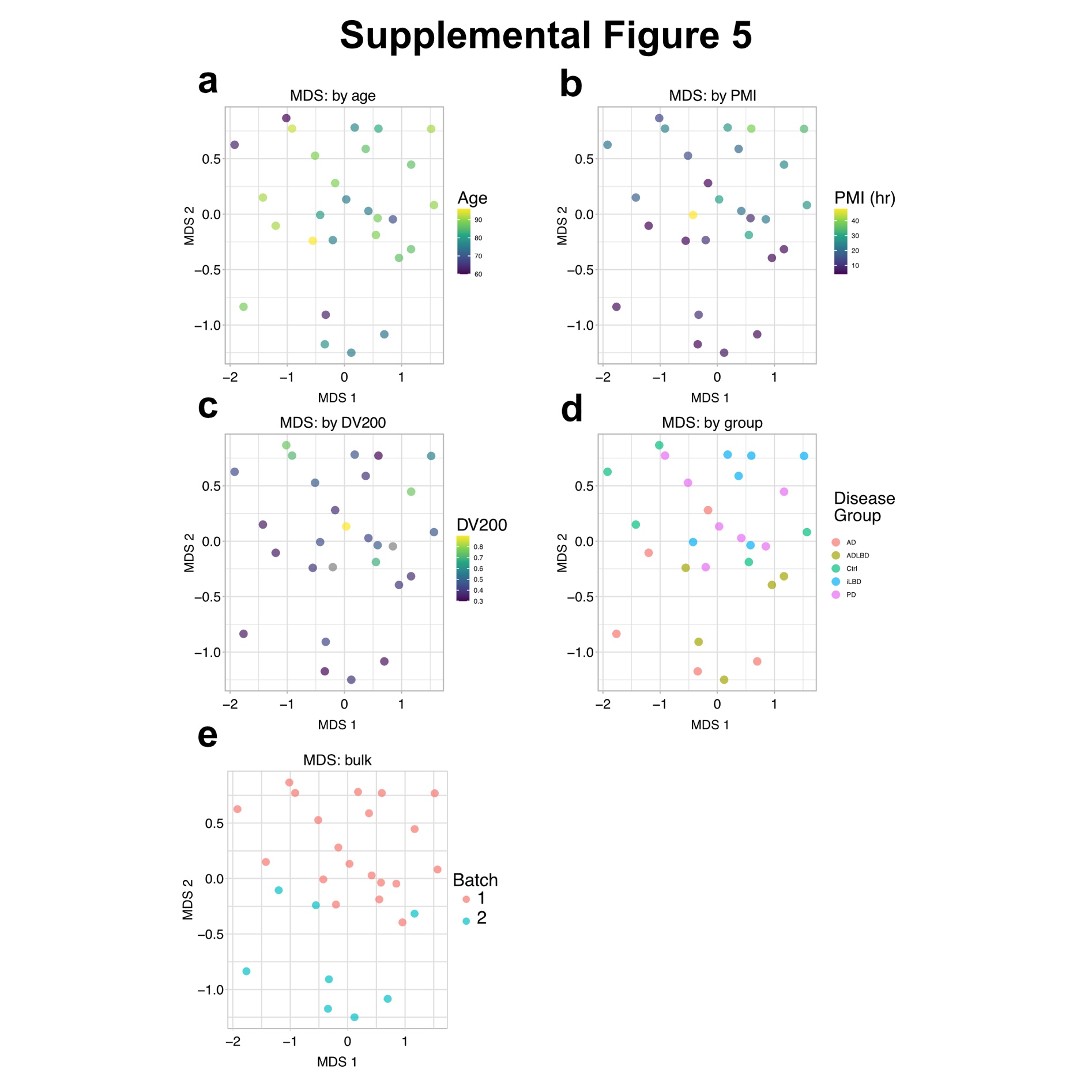


**Supplemental Figure 5:** a–e, Multidimensional scaling (MDS) analyses of spatial transcriptomic data colored by age, post-mortem interval (PMI), RNA quality (DV200), disease group, and Visium kit batch. Individual points represent biologically independent donor samples. A visible gradient along MDS2 was associated with PMI and Visium kit batch, consistent with partial confounding between these variables. No discrete clustering by disease group was observed.


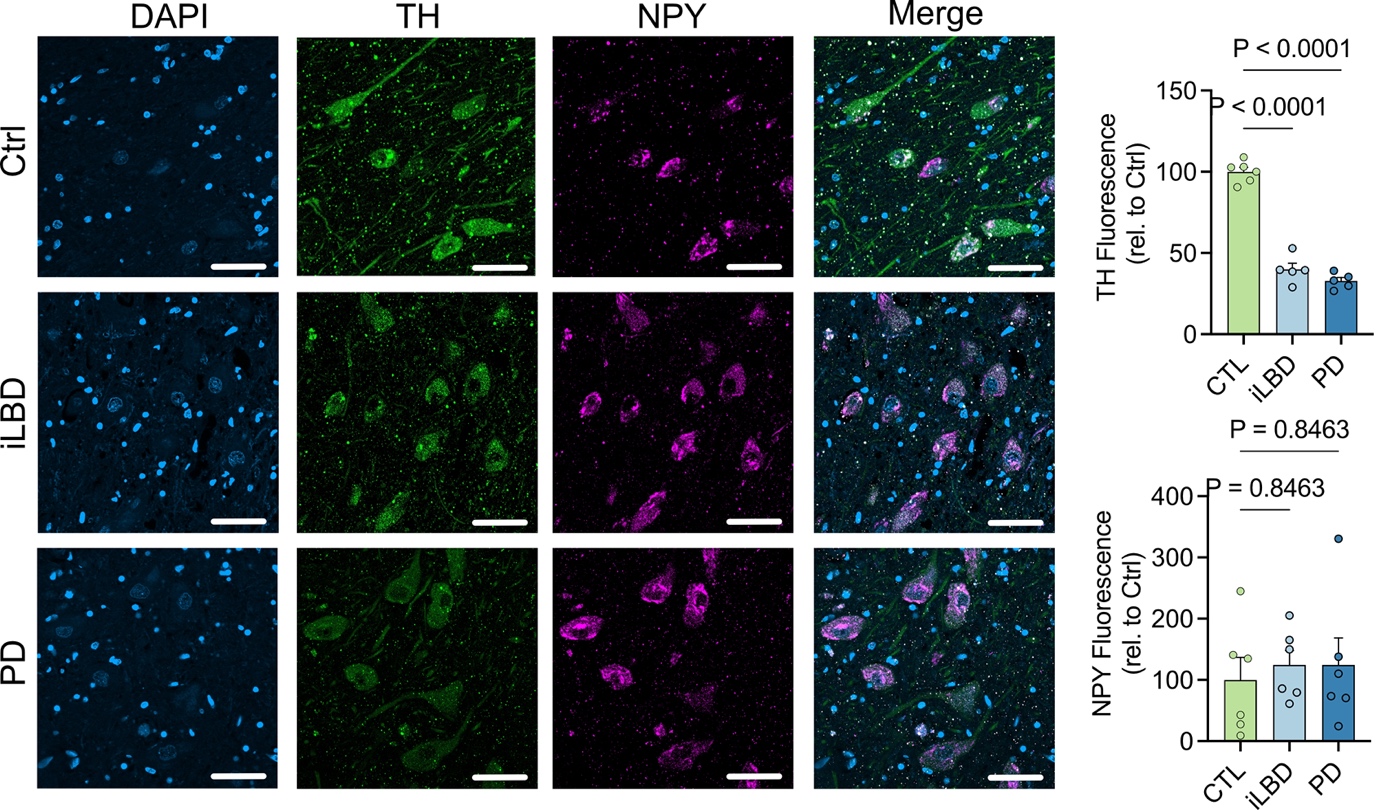


**Supplemental Figure 6:** Representative photomicrographs and corresponding bar graphs illustrating the abundance of TH and NPY in Ctrl, iLBD, and PD cases. Data are presented as mean ± SEM, and individual points represent biologically independent donor samples (n = 5 per group). Statistical significance was assessed using ordinary one-way ANOVA followed by Dunnett’s multiple-comparison test. Scale bar, 50 µm. Source data are provided as a Source Data file.


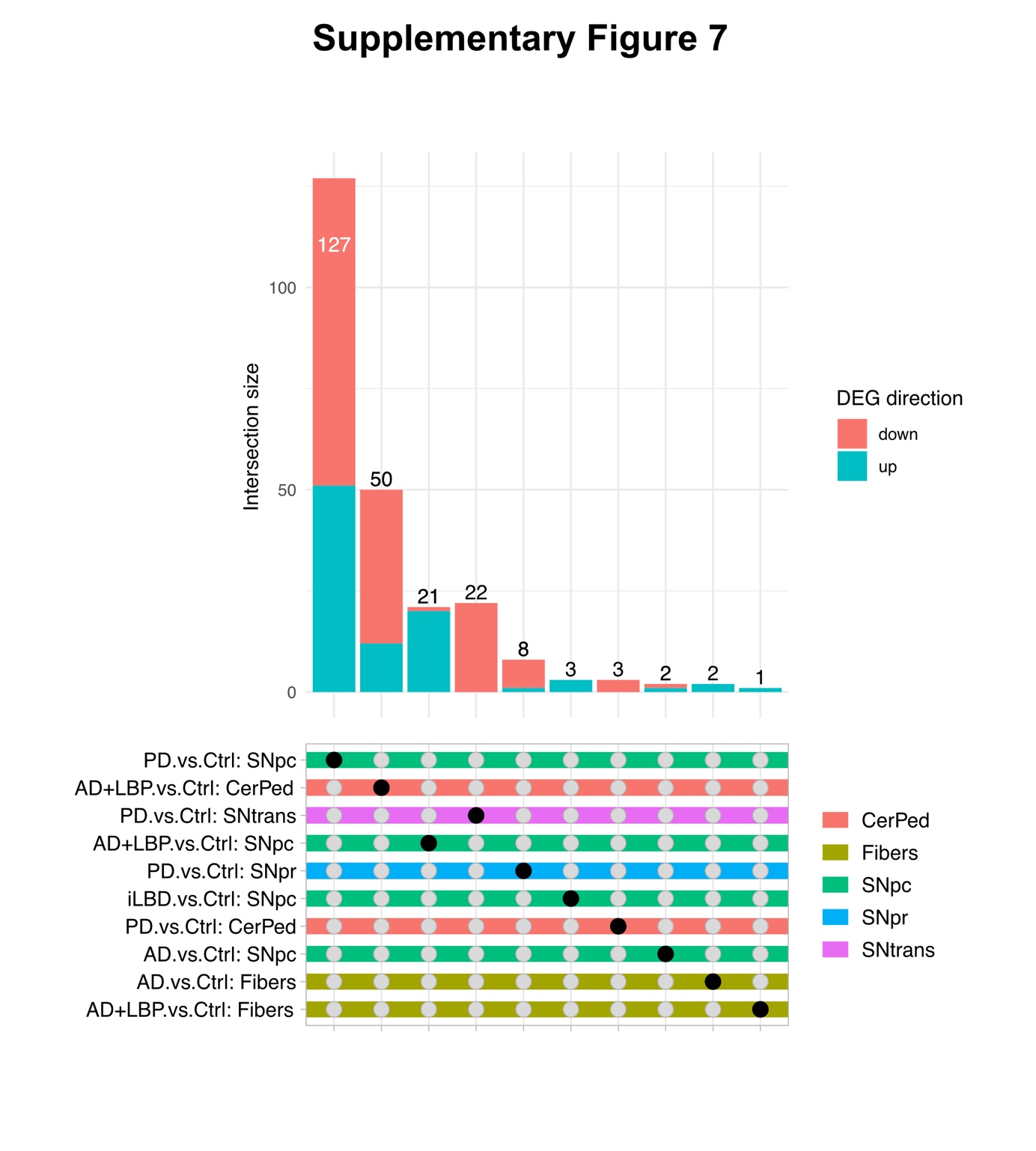


**Supplemental Figure 7:** Bar graph and accompanying table showing the number of differentially expressed genes (DEGs) between the indicated groups across the respective spatial gene clusters listed in the lower table (left). CerPed, cerebral peduncle; Fibers, fiber tracts; SNpc, substantia nigra pars compacta; SNpr, substantia nigra pars reticulata; SNtrans, substantia nigra transition zone. Red bars indicate upregulated genes, and green bars indicate downregulated genes. Differential expression analysis was performed as described in the Methods section.


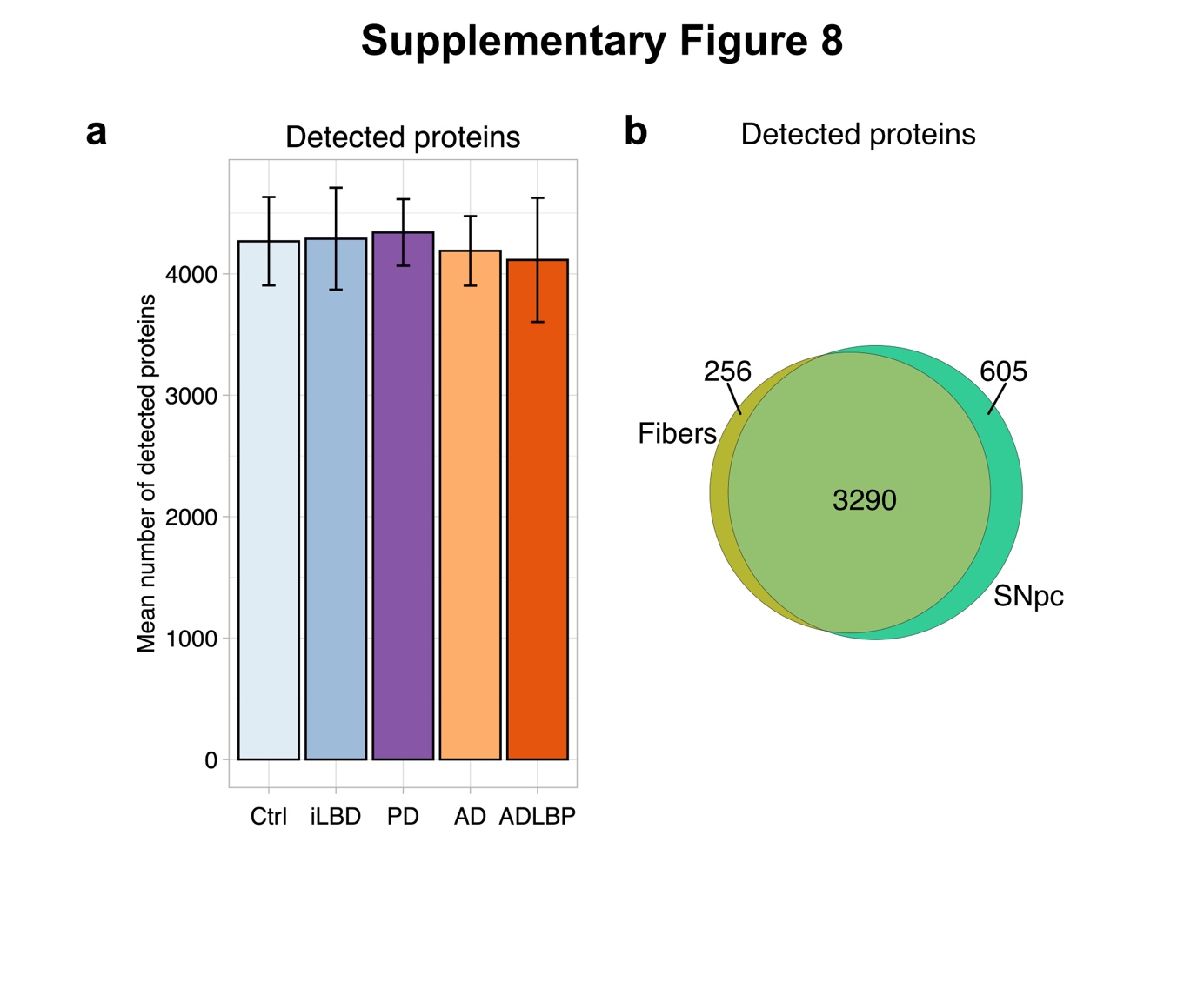


**Supplemental Figure 8:** a, Bar graph showing the number of proteins detected by mass spectrometry (MS) in each cohort. b, Venn diagram showing the overlap of detected proteins between clusters and the number of proteins unique to each cluster. Source data are provided as a Source Data file.

**
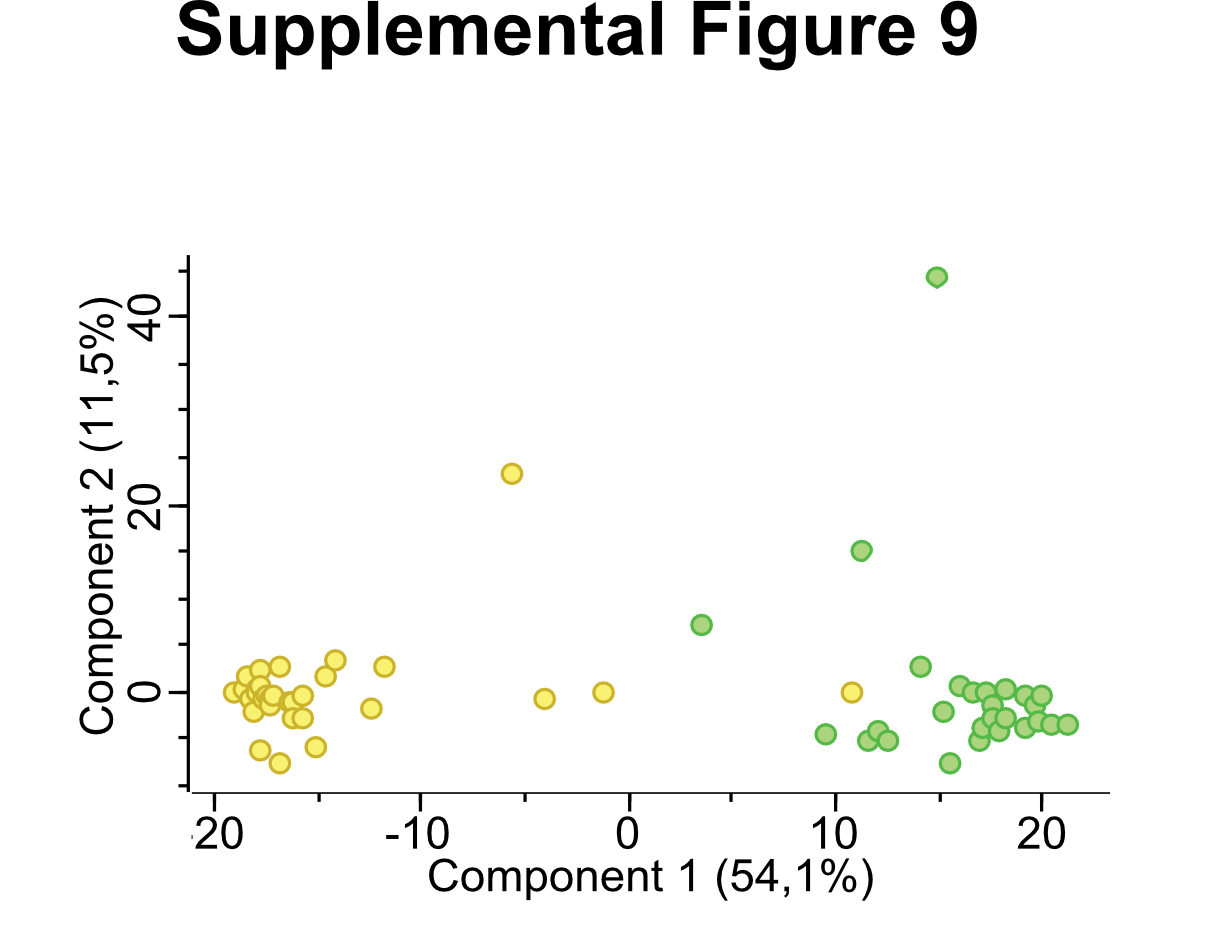
**

**Supplemental Figure 9:** Principal component analysis (PCA) of spatial proteomic data demonstrating segregation of samples by anatomical compartment, with substantia nigra pars compacta (yellow) separated from fiber-rich regions (green). Individual points represent biologically independent donor samples. No segregation by brain bank or diagnostic group was observed. Source data are provided as a Source Data file.

**
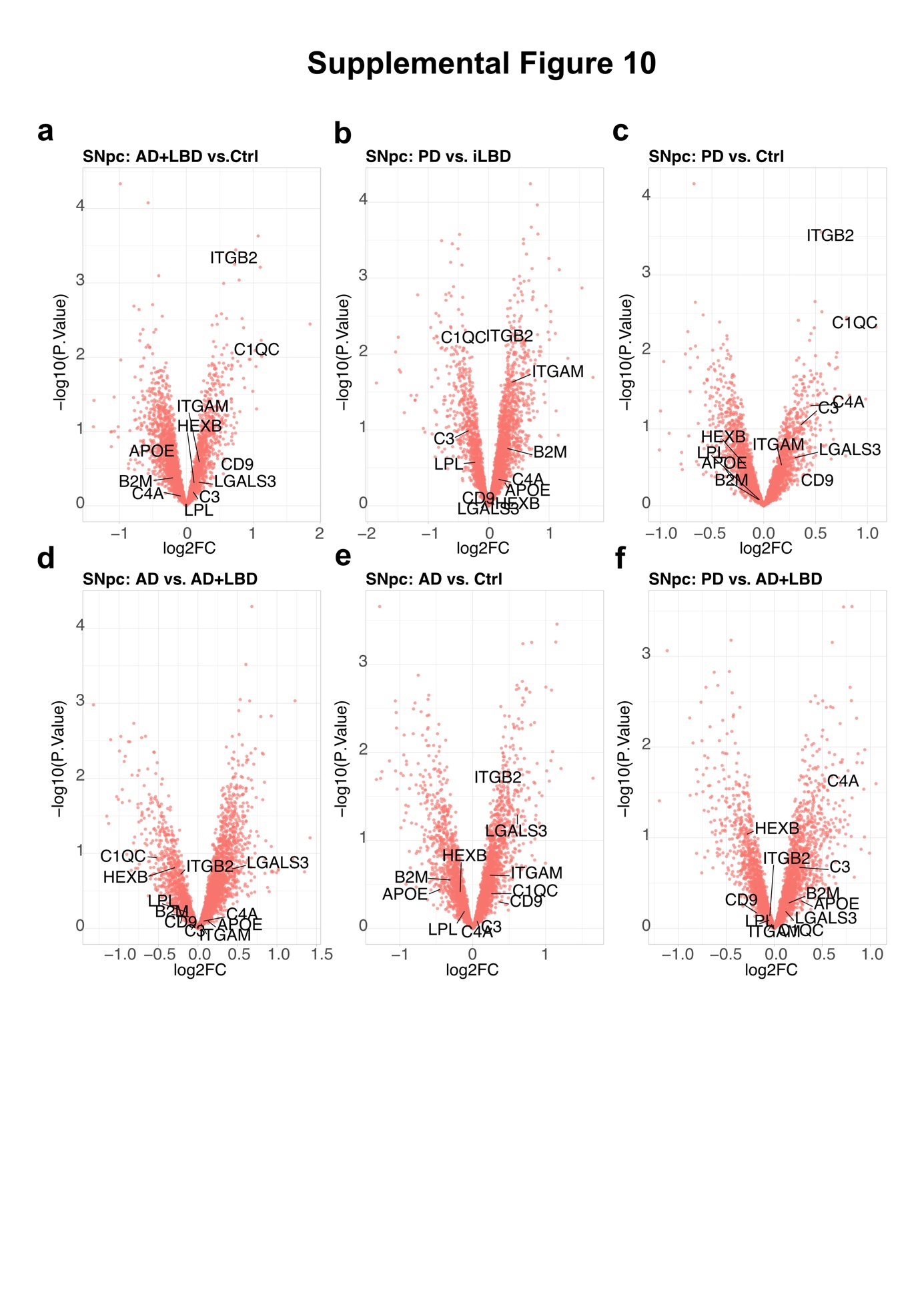
**

**Supplemental Figure 10:** a–f, Volcano plots showing the absence of significantly differentially expressed proteins (DEPs) in the SNpc cluster across the indicated comparisons. Differential expression analysis was performed as described in the Methods section. Source data are provided as a Source Data file.

**
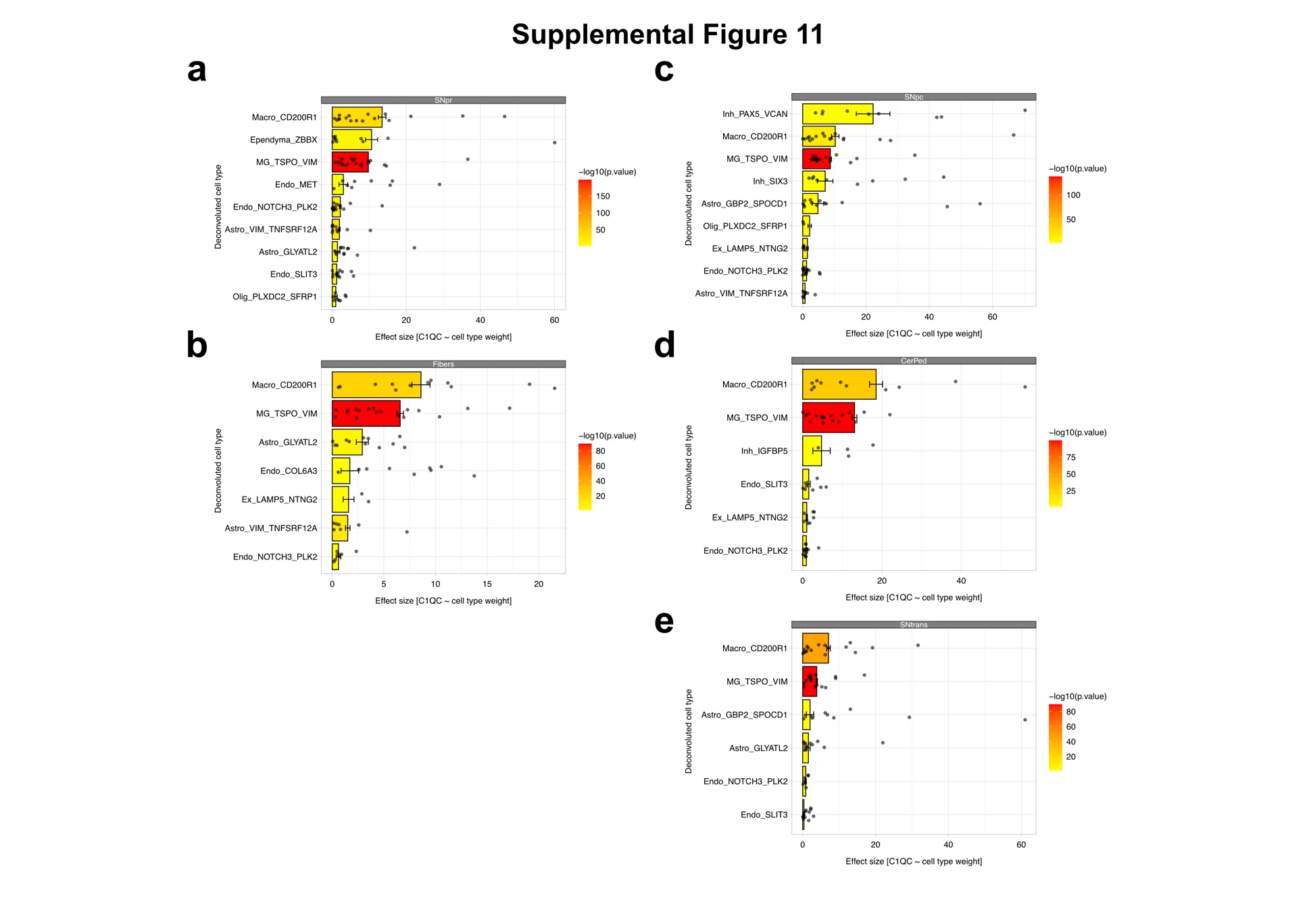
**

**Supplemental Figure 11**: Effect sizes for the relationship between C1QC expression and surrounding deconvoluted cell types across spatially defined clusters (a, Substantia nigra pars reticulata; b, Substantia nigra pars compacta; c, Substantia nigra transition zone; d, Fibers; e, Cerebellar peduncles). The x-axis indicates the effect size derived from linear modeling. The strongest and most significant enrichment of C1QC expression was observed for microglia (MG_TSPO_VIM) across all clusters, whereas enrichment for inhibitory neurons (Inh_PAX5_VCAN) was specific to the SNpc cluster and showed the largest effect size within this region.

**
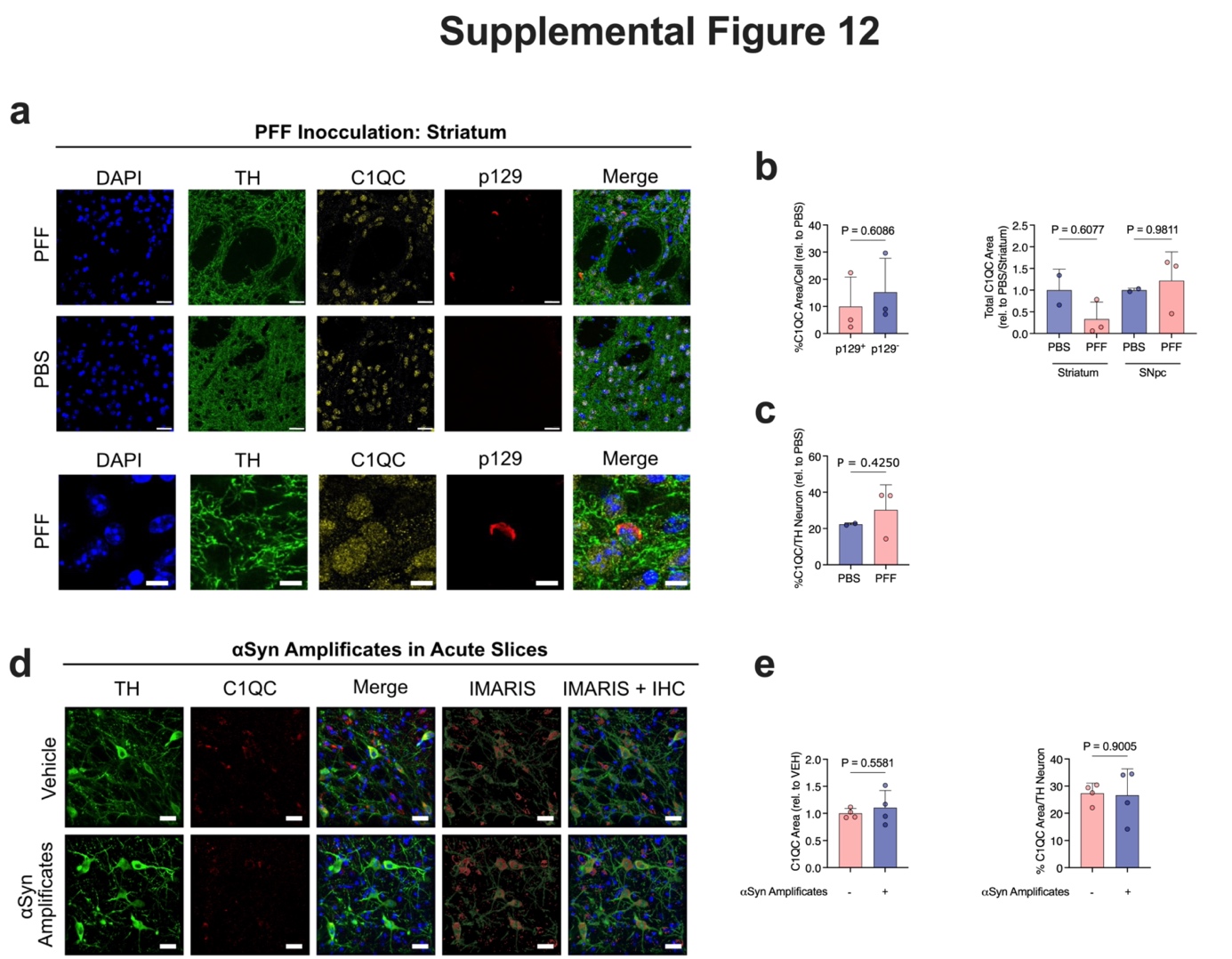
**

**Supplemental Figure 12:** a, Representative photomicrographs showing tyrosine hydroxylase (TH), C1QC, and phosphorylated Ser129–α-synuclein (pS129–αSyn) in animals injected with either preformed fibrils (PFFs) or PBS into the striatum, 3 months post-injection. Scale bar, 20 μm. Third row: higher-magnification images. Scale bar, 2 μm. b, Bar graphs showing the percentage of C1QC per cell (left) and the total C1QC area (right) in the striatum and substantia nigra pars compacta (SNpc). c, Bar graph showing the percentage of C1QC per TH-positive neuron. d, Representative photomicrographs showing TH, C1QC, and pS129–αSyn in acute brain slices treated for 6 h with human αSyn amplificates derived from seed amplification assays (SAA) of clinical Lewy body pathology (LBP) cases. Scale bar, 20 μm e, Bar graphs showing the C1QC area (left) and the percentage of C1QC per TH-positive neuron (right). Data in b, c, and e are presented as mean ± SEM, and individual points represent biologically independent animals or tissue samples. Statistical significance was assessed using unpaired two-sided t-tests. Source data are provided as a Source Data file.


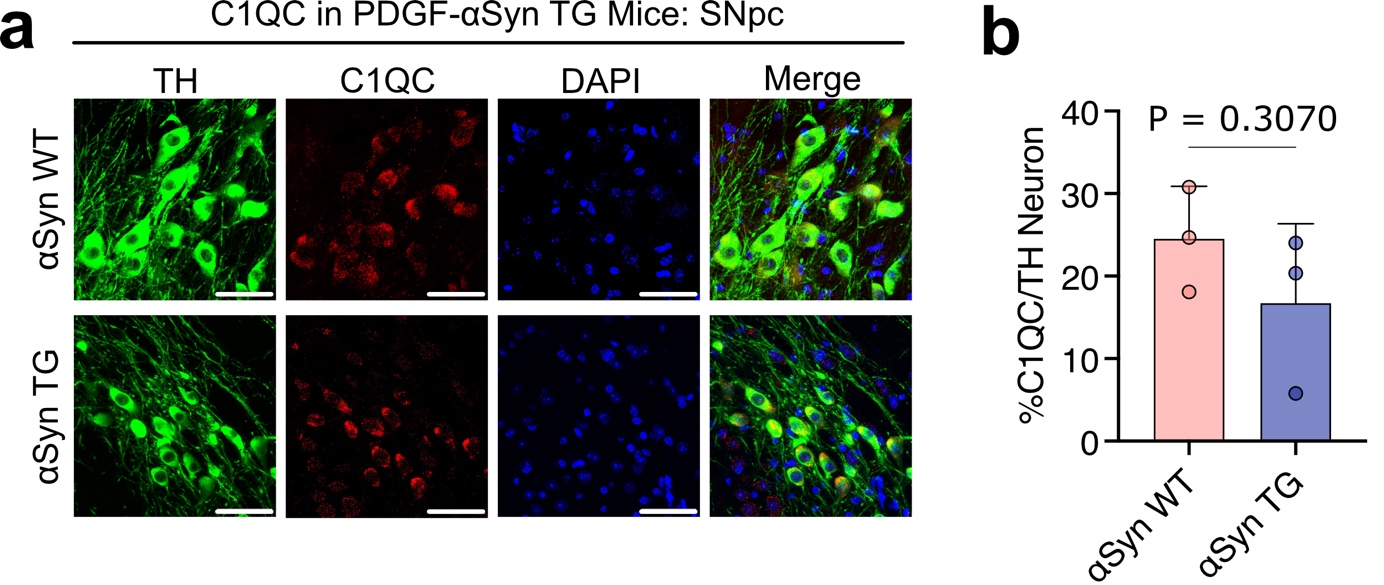


**Supplemental Figure 13** a,b, Representative photomicrographs and corresponding bar graphs depicting TH and C1QC expression in SNpc tissue sections from wild-type (WT) and αSyn transgenic (Tg) mice. No significant difference in C1QC abundance was observed between the two groups. Data are presented as mean ± SEM, and individual points represent biologically independent animals. Statistical significance in panel b was assessed using unpaired two-sided t-tests. Scale bar, 50 μm. Source data are provided as a Source Data file.


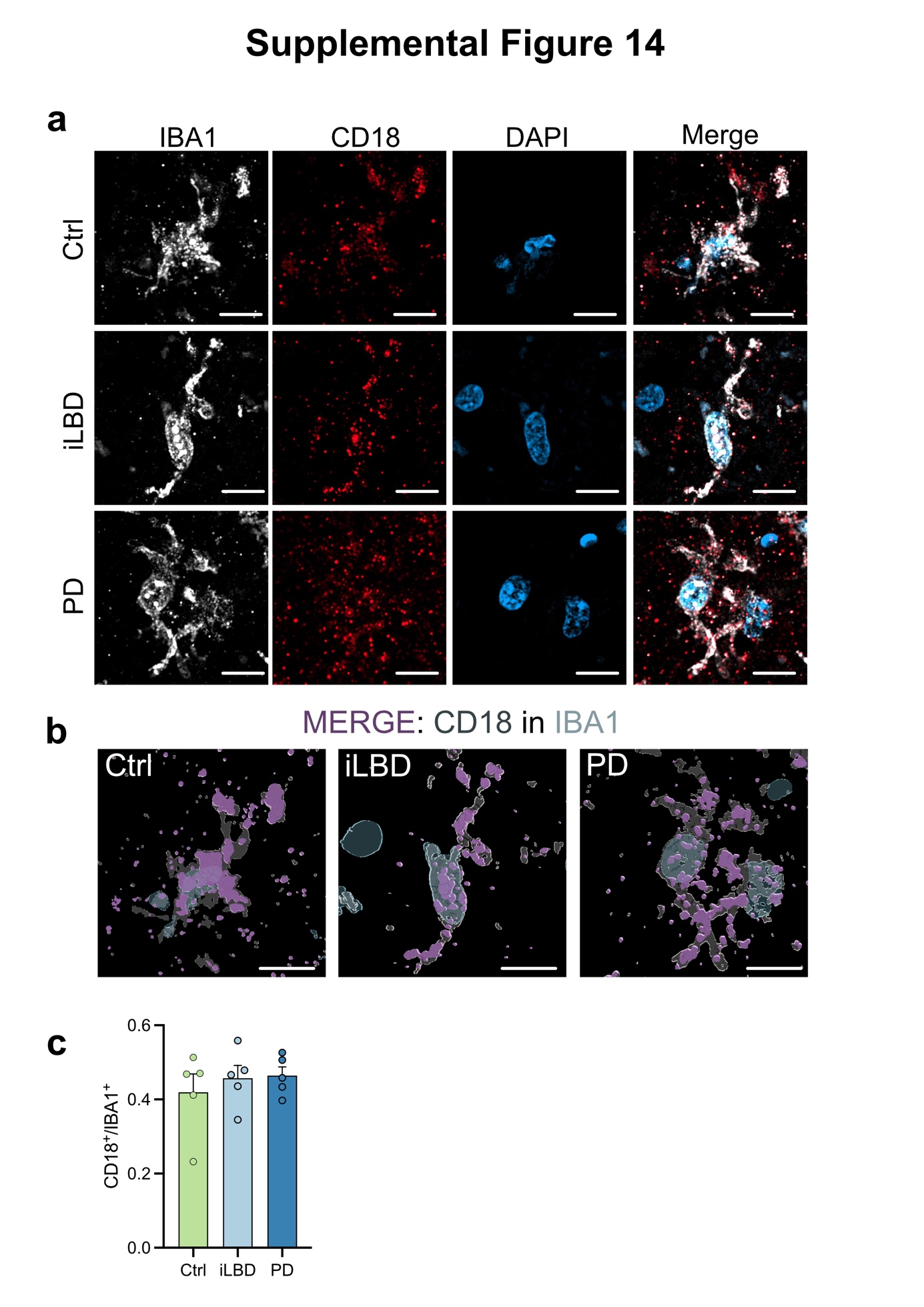


**Supplemental Figure 14:** a, Representative photomicrographs of substantia nigra (SN) tissue sections from Ctrl, iLBD, and PD cases showing IBA1, CD18, and DAPI staining. Scale bar, 10 μm. b, IMARIS reconstruction of SNpc tissue sections illustrating CD18/IBA1 double-positive signals (purple). Scale bar, 10 μm c, Quantification of CD18/IBA1 double-positive fluorescence across groups. Data are presented as mean ± SEM, and individual points represent biologically independent donor samples (n = 5 per group). Statistical significance was assessed using ordinary one-way ANOVA followed by Dunnett’s multiple-comparison test. Source data are provided as a Source Data file.


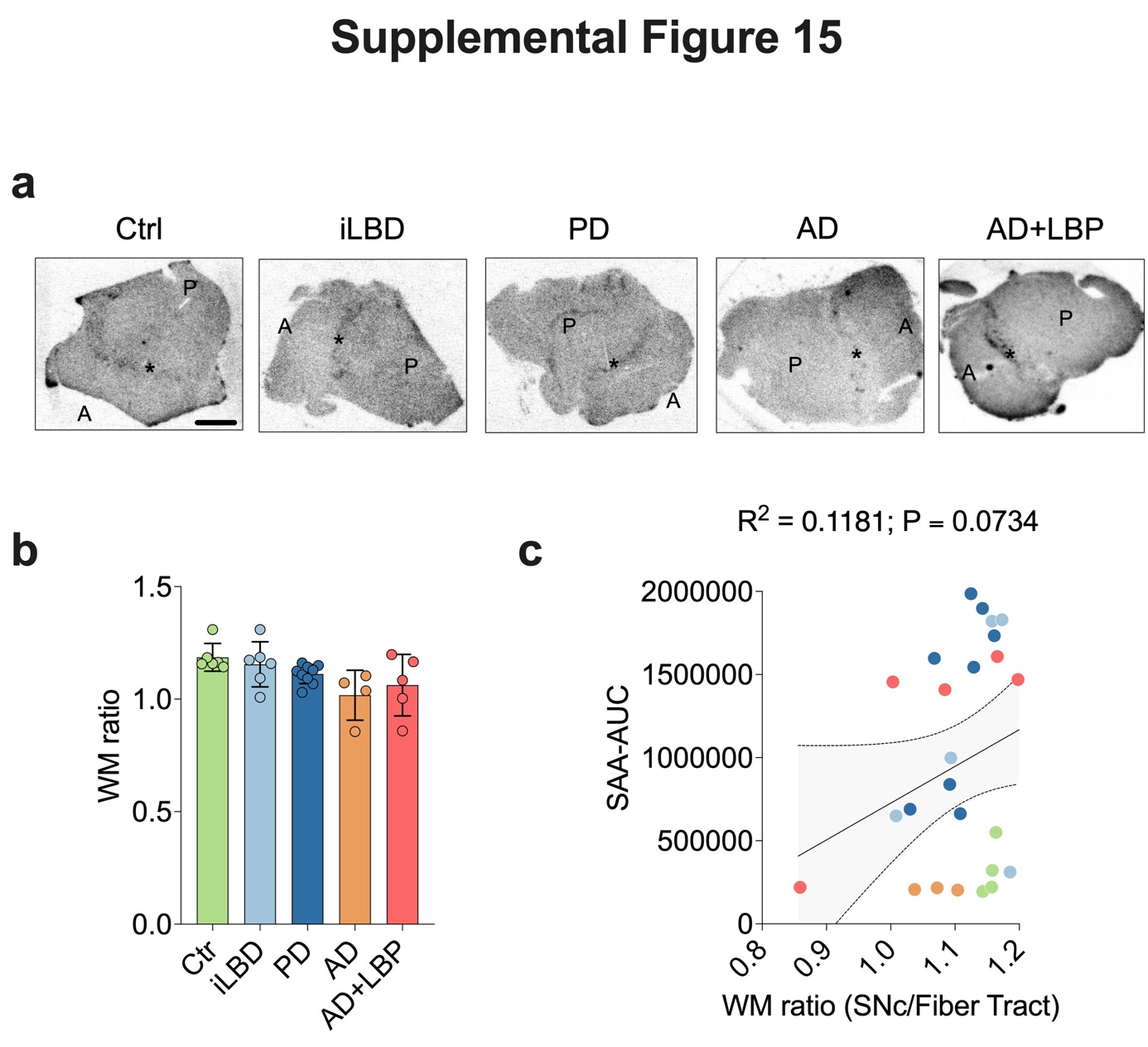


**Supplemental Figure 15:** a,b, Representative photomicrographs and corresponding bar graphs from all five cohorts showing comparable binding of [18F]DPA-714 across cohorts. Scale bar, 4 mm. Data are presented as mean ± SEM, and individual points represent biologically independent donor samples. Statistical significance was assessed using ordinary one-way ANOVA followed by Dunnett’s multiple-comparison test. c, Graph showing no significant correlation between mean seed amplification assay area under the curve (SAA–AUC) and translocator protein (TSPO) signal. Coefficient of determination (R²) were used to assess correlation. Source data are provided as a Source Data file.
