# Supplementary material for: Spatial multi-omics identifies early synaptic pruning and context-specific dopaminergic vulnerability in synucleinopathies
Source: Nat Commun. 2026 Jul 21;17:6976. doi: 10.1038/s41467-026-74961-6 (PMC13392254; doi:10.1038/s41467-026-74961-6)
Supplement: Supplementary file 2 — Reporting Summary [file 41467_2026_74961_MOESM2_ESM.pdf]

nature portfolio

Corresponding author(s): Thomas Koeglsperger

Last updated by author(s): Jun 12, 2026

Reporting Summary

Nature Portfolio wishes to improve the reproducibility of the work that we publish. This form provides structure for consistency and transparency in reporting. For further information on Nature Portfolio policies, see our Editorial Policies and the Editorial Policy Checklist.

Statistics

For all statistical analyses, confirm that the following items are present in the figure legend, table legend, main text, or Methods section.

n/a	Confirmed	
☐	☒	The exact sample size (*n*) for each experimental group/condition, given as a discrete number and unit of measurement
☐	☒	A statement on whether measurements were taken from distinct samples or whether the same sample was measured repeatedly
☐	☒	The statistical test(s) used AND whether they are one- or two-sided<br>*Only common tests should be described solely by name; describe more complex techniques in the Methods section.*
☐	☒	A description of all covariates tested
☐	☒	A description of any assumptions or corrections, such as tests of normality and adjustment for multiple comparisons
☐	☒	A full description of the statistical parameters including central tendency (e.g. means) or other basic estimates (e.g. regression coefficient) AND variation (e.g. standard deviation) or associated estimates of uncertainty (e.g. confidence intervals)
☐	☒	For null hypothesis testing, the test statistic (e.g. *F*, *t*, *r*) with confidence intervals, effect sizes, degrees of freedom and *P* value noted<br>*Give P values as exact values whenever suitable.*
☒	☐	For Bayesian analysis, information on the choice of priors and Markov chain Monte Carlo settings
☐	☒	For hierarchical and complex designs, identification of the appropriate level for tests and full reporting of outcomes
☐	☒	Estimates of effect sizes (e.g. Cohen's *d*, Pearson's *r*), indicating how they were calculated

Our web collection on statistics for biologists contains articles on many of the points above.

Software and code

Policy information about availability of computer code

Data collection	Sample sizes were based on post-mortem tissue availability. No predetermined calculations were performed, but cohort sizes (n=5–9) align with comparable studies. All statistical analyses were conducted in R (v4.4.1).
Data analysis	Statistical tests included Welch's t-test, one-way ANOVA, and Pearson's correlation. Significance thresholds were set at p < 0.05 unless otherwise indicated. All statistical analyses were conducted in R (v4.4.1) or GraphPad Prism. For bioinformatic data analysis, after correction for multiple testing using Benjamini-Hochberg's method, an FDR < 0.1 with an absolute log2 fold-change of >0.5 was accepted as statistically significant.<br>Code needed to reproduce the spacial transcriptomic/proteomic analysis is available at: https://github.com/fstrueb/spatial_nigra

For manuscripts utilizing custom algorithms or software that are central to the research but not yet described in published literature, software must be made available to editors and reviewers. We strongly encourage code deposition in a community repository (e.g. GitHub). See the Nature Portfolio guidelines for submitting code & software for further information.

Data

Policy information about availability of data

All manuscripts must include a data availability statement. This statement should provide the following information, where applicable:

- Accession codes, unique identifiers, or web links for publicly available datasets
- A description of any restrictions on data availability
- For clinical datasets or third party data, please ensure that the statement adheres to our policy

Spatial transcriptomics data generated in this study have been deposited in the NCBI Sequence Read Archive (SRA) under accession code PRJNA1357890. Proteomics data generated in this study have been deposited in the ProteomeXchange Consortium via the PRIDE partner repository under accession code PXD062998 (http://proteomecentral.proteomexchange.org/cgi/GetDataset?ID=PXD062998). Source data are provided with this paper.

Code availability:
The code necessary to reproduce the findings is available at https://github.com/fstrueb/spatial_nigra.

Research involving human participants, their data, or biological material

Policy information about studies with human participants or human data. See also policy information about sex, gender (identity/presentation), and sexual orientation and race, ethnicity and racism.

Reporting on sex and gender	Both male and female donors were included across all diagnostic groups. Sex distribution did not differ significantly between cohorts (Fisher's exact test, p = 0.741). No sex-based analyses were specifically performed, as the study focused on disease-stage molecular changes rather than sex-specific differences.
Reporting on race, ethnicity, or other socially relevant groupings	Race or ethnicity data were not provided by the brain banks and were not relevant to the aims of this molecular neuropathological study. All samples were anonymized prior to receipt by the investigators.
Population characteristics	Human post-mortem midbrain samples were obtained from the Netherlands Brain Bank and the Parkinson's Disease and Multiple Sclerosis Brain Bank (Imperial College London). Cohorts included:<br><br>Controls (LBP Braak stage 0, n=6)<br><br>iLBD (Braak stage 1–2, n=7)<br><br>PD (Braak stage 3–6, n=9)<br><br>AD (Tau Braak stage 6, n=5)<br><br>AD+LBP (Tau Braak 6, LBP Braak 6, n=5)<br>Mean ages ranged from 78–84 years.
Recruitment	Samples were collected by brain banks following informed consent from donors or next of kin for use in scientific research. Researchers did not participate in donor recruitment.
Ethics oversight	All procedures were approved by the Ethics Committee of Ludwig-Maximilians-Universität (LMU) Munich and complied with the Declaration of Helsinki. Brain banks provided material under established ethical frameworks and local regulations governing post-mortem human tissue research.

Note that full information on the approval of the study protocol must also be provided in the manuscript.

Field-specific reporting

Please select the one below that is the best fit for your research. If you are not sure, read the appropriate sections before making your selection.

☒ Life sciences ☐ Behavioural & social sciences ☐ Ecological, evolutionary & environmental sciences

For a reference copy of the document with all sections, see nature.com/documents/nr-reporting-summary-flat.pdf

Life sciences study design

All studies must disclose on these points even when the disclosure is negative.

Sample size	Sample sizes were determined by the availability and quality of post-mortem human midbrain tissue obtained from established brain banks. Cohorts consisted of Controls (n = 6), iLBD (n = 7), PD (n = 9), AD (n = 5), and AD+LBP (n = 5). These numbers are consistent with prior neuropathological and spatial transcriptomic studies of similar scope and statistical power. No formal power calculation was performed, but group sizes provided sufficient statistical discrimination in differential expression analyses.

Data exclusions	No data were excluded from analyses unless samples failed pre-defined quality control criteria (e.g., RNA DV200 < 30% or low transcript capture efficiency in spatial transcriptomics). Four samples were excluded for these reasons (1 control, 1 iLBD, 2 PD). All exclusion criteria were pre-established and applied uniformly.
Replication	Experimental findings were independently validated across multiple modalities, including transcriptomic, proteomic, and imaging-based analyses. RT-QuIC, immunohistochemistry, and proximity ligation assays were performed in biological replicates. All attempts at replication were successful and yielded consistent results. Replication is indicated together with the respective experiments in the manuscript. At least n = 3 biological repeats were inlcuded.
Randomization	As the study used archival post-mortem human tissue, randomization was not applicable. Group assignment was based solely on neuropathological diagnosis verified by brain bank pathologists.
Blinding	Investigators were blinded to diagnostic group during imaging, α-synuclein RT-QuIC, and quantitative analyses. Group codes were revealed only after completion of data processing and statistical evaluation.

Reporting for specific materials, systems and methods

We require information from authors about some types of materials, experimental systems and methods used in many studies. Here, indicate whether each material, system or method listed is relevant to your study. If you are not sure if a list item applies to your research, read the appropriate section before selecting a response.

Materials & experimental systems

n/a	Involved in the study	
☐	☒	Antibodies
☒	☐	Eukaryotic cell lines
☒	☐	Palaeontology and archaeology
☐	☒	Animals and other organisms
☐	☒	Clinical data
☒	☐	Dual use research of concern
☒	☐	Plants

Methods

n/a	Involved in the study	
☒	☐	ChIP-seq
☒	☐	Flow cytometry
☒	☐	MRI-based neuroimaging

Antibodies

Antibodies used	The following primary antibodies were used in this study: TH (sheep, 1:500, Invitrogen, Cat. No. PA1-4679), C1QC (rabbit, 1:100, Life Technologies, Cat. No. PA5-106648), gephyrin (mouse, 1:200, Synaptic Systems, Cat. No. 147011), GAD67 (mouse, 1:100, Sigma, Cat. No. MAB5406B), IBA1 (guinea pig, 1:500, Synaptic Systems, Cat. No. 234308), CD68 (rat, 1:500, BioRad, Cat. No. MCA1957), CD18 (rabbit, 1:100, Abcam, Cat. No. AB131044), and NPY (rabbit, 1:100, Invitrogen, Cat. No. PA5-85762).<br><br>Secondary Antibodies:<br>The following secondary antibodies were used in this study: donkey anti-rabbit Alexa Fluor 647 (1:500, Invitrogen, Cat. No. A31573), donkey anti-rabbit Alexa Fluor 488 (1:500, Invitrogen, Cat. No. A21206), donkey anti-sheep Alexa Fluor 555 (1:500, Invitrogen, Cat. No. A21436), donkey anti-sheep Alexa Fluor 488 (1:500, Invitrogen, Cat. No. A11015), donkey anti-rat Alexa Fluor 647 (1:500, Invitrogen, Cat. No. A48272), donkey anti-mouse Alexa Fluor 594 (1:500, Invitrogen, Cat. No. A21203), goat anti-guinea pig Alexa Fluor 488 (1:500, Invitrogen, Cat. No. A11073), and streptavidin Alexa Fluor 647 (1:500, Invitrogen, Cat. No. S32357).
Validation	Antibody performance verified by supplier validation and expected staining patterns.

Animals and other research organisms

Policy information about studies involving animals; ARRIVE guidelines recommended for reporting animal research, and Sex and Gender in Research

Laboratory animals	All animal experiments were performed in accordance with the guidelines of the animal committee at LMU Munich and were approved by the local authorities (Regierung von Oberbayern) under protocol number ROB-55.2-2532.Vet_02-20-30. Wild-type C57BL/6J mice were obtained from Jackson Labs, and αSyn transgenic mice overexpressing human αSyn under the PDGFB promoter were maintained on a C57BL/6J background. Male and female mice aged 2-3 months were used for experiments. Animals were housed under specific pathogen-free conditions with ad libitum access to food and water under a 12 h light/12 h dark cycle. Mice expressing αSyn under the human Platelet-Derived Growth Factor (PDGF-αSynTg) were purchased from Jackson Laboratory (B6;D2-Tg(PDGFB-SNCA)4Ema/RoriJ; Strain No.: #038775).
Wild animals	N/A
Reporting on sex	N/A
Field-collected samples	N/A

Ethics oversight	All procedures complied with EU and national animal research guidelines.

Note that full information on the approval of the study protocol must also be provided in the manuscript.

Clinical data

Policy information about clinical studies

All manuscripts should comply with the ICMJE guidelines for publication of clinical research and a completed CONSORT checklist must be included with all submissions.

Clinical trial registration	N/A
Study protocol	N/A
Data collection	N/A
Outcomes	N/A

Plants

Seed stocks	N/A
Novel plant genotypes	N/A
Authentication	N/A
