# Supplementary material for: Spatial multi-omics identifies early synaptic pruning and context-specific dopaminergic vulnerability in synucleinopathies
Source: Nat Commun. 2026 Jul 21;17:6976. doi: 10.1038/s41467-026-74961-6 (PMC13392254; doi:10.1038/s41467-026-74961-6)
Supplement: Supplementary file 3 — Transparent Peer Review file [file 41467_2026_74961_MOESM3_ESM.pdf]

nature portfolio

Peer Review File

Spatial Multi-Omics Identifies Early Synaptic Pruning and Context-Specific Dopaminergic Vulnerability in Synucleinopathies

Corresponding Author: Dr Thomas Koeglsperger

This file contains all reviewer reports in order by version, followed by all author rebuttals in order by version.

Version 0:

Reviewer comments:

Reviewer #1

(Remarks to the Author)
The authors applied a multi-omic spatial profiling approach, integrating transcriptomics, proteomics, and α-synuclein seed amplification assays (SAA), to post-mortem midbrain tissue from neurologically unaffected individuals, iLBD cases, patients with PD, AD, or AD+LBP. This is a very interesting and comprehensive study, which could be a good resource for the field. Following are my comments:

1. The correlations between pigmented neuron counts and both SAA AUC and Braak stage were attenuated or lost in AD and AD+LBP cases. It will be interesting to evaluate the extend of tau and Abeta pathology in all the cases examined, particularly the AD cases. Could the loss of correlation be attribute to the existence of AD pathology?

2. 20 DEGs were found in the AD+LBP vs. Ctrl comparison. 2 DEGs in AD vs controls comparation. This data suggests that LBP in AD further affect the gene expression compared with AD only. However, no DEGs were found in the comparison of AD cases with and without LBP. This is somehow confusing. Could this be due to the very limited number of cases used in this study?

3. The authors need to verify at least some of the genes identified by spatial transcriptomics using immunofluorescent staining or in situ hybridization.

Reviewer #2

(Remarks to the Author)
Summary: Rumpf and colleagues present a study of 10x Visium spatial transcriptomics and proteomics in the substantia nigra of control, incidental Lewy body disease, Parkinson’s disease, Alzheimer’s disease, and Alzheimer’s disease with Lewy body pathology. This type of profiling study holds great promise to identify novel pathways perturbed in Parkinson’s disease. A particular strength of the study is the inclusion of several comparator groups for PD, beyond simply including matched controls. Several complementary assays, such as seed amplification, proximity ligation, and immunofluorescence assays are employed to aid interpretation. Despite these strengths, there are several major limitations of the study which diminish the ability to interpret results, and several similar published studies detract from the likely impact. Major and minor points are listed below.

Major Points:
1. There are some overall issues with cohort design that make subsequent interpretation difficult. 1) The number of cases overall is low (32 cases total). 2) Tissues for AD cases were obtained from a different brain bank with much different processing. 3) High post-mortem intervals (~24 hours for main comparators) and fixation times (28 days+) could contribute to poor tissue quality. Were these assessed by RIN score or other QC metrics?
2. Spatial transcriptomics is a relatively recent technology that has enabled new insights into neurodegenerative disease. However, several groups have already published on transcriptomic shifts in Parkinson’s disease. In particular, two studies have used the same technology (10x Visium) in the substantia nigra of Parkinson’s disease and control tissues: PMID:

40759663, bioRxiv: https://doi.org/10.1101/2024.07.28.605055. Neither of these studies are cited in the current manuscript.
3. Major findings from the transcriptomics data seem to be largely related to the difference in cell type abundance, in particular the loss of dopaminergic neurons in the substantia nigra. The utility of differential gene expression across brain regions with known disparate cell type abundances is unclear.
4. Proteomics is a less novel technology, and many groups have published proteomic studies of the Parkinson's disease brain. In particular, these three studies are in the substantia nigra, and one of them even employs a similar laser capture strategy: PMID: 36423813, 32132230, 18275612. Again, these studies are not cited in the current manuscript.
5. The purification of α-synuclein is not well-described. It is insufficient to refer to a previous paper. What is the construct being purified? It is noted that it is "wild-type α-synuclein," however, the purification is through a Ni-NTA column, which suggests the protein is His-tagged. Further details are required.
6. Lines 222-224: "Of the 54,580 probes included in the FFPE Visium v2 assay, 8,652 were removed as recommended by the 10X QC department (personal communication)." Please provide additional information about why these probes were removed.
7. Fig. 1f: Several control samples show some positivity in the SAA, but this is not discussed in the results section.
8. Lines 442-444: "This notion was further corroborated by comparing the group-wise log fold-changes of DEGs with the Pearson's correlation coefficient of gene counts to the number of pigmented cells." Does this suggest that most of the differential gene expression is due to a loss of pigmented neurons?
9. Supplementary Fig. 7: This is noted in the text as a pathway analysis, but it appears to be a cell type deconvolution.
10. It is surprising that SNCA, encoding α-synuclein did not show a significant difference in the PD vs. CTRL proteomic comparison. Do the authors think this is due to the extraction method which may have left insoluble proteins behind? Or a detection limit of the method? Or due to the difference in cell abundance in this region?
11. C1QC staining in both human tissue and mouse models appears to be largely in neuronal cell bodies. Is this the expected pattern? Have the authors identified a method to validate the specificity of this staining?
12. It is postulated by the authors that "microglial phagocytosis of GABAergic postsynapses is enhanced, leading to a loss of inhibitory input onto SNpc dopaminergic neurons and, as a potential consequence, disinhibition of these cells." However, to support this hypothesis, there would need to be evidence of an overall decrease of GABAergic terminals/dopaminergic neuron.

Minor Points:
1. References 16 and 17 are the same paper.
2. Supplementary Fig. 3: No legend is provided for the colors of the dots.
3. Supplementary Fig. 3: There are inconsistent nomenclatures and fonts. Some correlations are reported with an R^2, while some are reported as r. A comma is used in panel c.
4. The directionality for the volcano plots is not always clear. For example, in Fig. 4e, the comparison is noted as iLBD vs. Control, but apparently proteins enriched in iLBD are on the right.
5. Please note the log scale for volcano plots. They are noted as "logFC." Presumably, this is Log2 fold change, but this is not noted.

Reviewer #3

(Remarks to the Author)
In this manuscript, Rumpf, Strubing et al. examine the cellular and molecular changes that occur in the substantia nigra in different synucleinopathies. They utilize multiple techniques to extract spatial histopathological, transcriptomic, proteomic information from consecutive FFPE sections of patients with either incidental Lewy body disease (iLBD), Parkinson's disease (PD), Alzheimer's (AD), AD+LBD, and controls. From this rich collective dataset, they report specific signatures associated with each disease type, perhaps the most significant being the upregulation and enrichment of complement C1q in the SNpc of iLBD subjects. Surprisingly, this was not observed in the SNpc of subjects with AD+LBP despite these samples scoring having similar Braak LB staging and seed amplification assay (SAA) status. In follow up studies, they show increased C1q localization to ventral tier dopamine neurons of iLBD (but not AD+LBD), and C1q interaction with the GABAergic postsynaptic scaffold protein Gephyrin within CD68+ puncta, suggesting that they are engulfed in microglial lysosomes. The authors conclude that this neuroinflammatory response represents a unique and novel feature that distinguishes iLBD from AD+LBP, and the intriguing possibility that this occurs in the absence of frank Lewy pathology as this is not observed in AD+LBD. These findings therefore address the central question of whether AD+LBP and iLBD/PD follow distinct pathogenetic pathways and introduce a powerful methodological roadmap for examining this. On the other hand, there are a number of gaps in the data and interpretation in this current manuscript that need to be addressed in order to more fully substantiate the main claims made.

Main points:

- The observation that C1q is upregulated in SNpc of iLBD vs AD+LBP is an important one with potentially profound mechanistic and clinical implications. However, a detailed comparison with C1q status in the PD samples is crucial to establish whether this remains elevated or is transient. Either outcome would be significant but would provide important insights into whether iLBD/PD represents a distinct disease axis compared to AD+LBD or that C1q is a specific marker of only iLBD. This is also The data in question should already be contained in Supp Fig 6, but none of the targets are labeled in this figure.

- Since the complement pathway is being invoked as being specific to iLBD (and perhaps PD), levels of other complement components should also be highlighted as they have been widely described in the context of AD. For example, was staining

or PLA with C3 performed? Similarly, was C1q elevated in the neocortex or amygdala of AD+LBD? Given the very significant differences in PMI and fixation time between AD and non-AD samples, these could also serve as an internal control for validating the integrity of the targets across samples from the two sites.

- LB dementia (DLB) also features dopamine neuron loss but discussion about it is notably absent from the study. Since one of the major conclusions is that SNpc neuron loss/C1q activation is not correlated with synucleinopathy, please provide a rationale that the DLB samples were not examined.

- Line 517: "Region-Specific Upregulation of C1QC in iLBD Suggests Early, αSyn-Independent Inflammatory Signaling." This is a bold and exciting hypothesis and based largely on the observation here that LBP, when present in AD, does not present with this signature nor dopamine cell loss. However, an important caveat is that LBP is present in iLBD - thus, an equally valid interpretation is that LBP is not sufficient (but possibly still necessary) for this phenomenon.

- Fig 6: The observation that Gephyrin colocalizes to microglial CD68+ lysosomes is interesting. However, the Gephyrin+ objects appear to be much larger than the CD68 puncta, which makes it hard to reconcile as inside the lysosomal lumen.

Other points:
- The use of multiple spatial-omics (transcriptomics, proteomics), histological (IHC, PLA), and biochemical (SAA) approaches on consecutive tissue samples is a core strength of the manuscript. This should be emphasized by showing data from consecutive (or nearby) sections in Fig 1b-d so that readers can appreciate the correlation between these sources of data. The images currently shown do not appear to be taken from the same sample/subject.

- Line 382: "In contrast, these associations were attenuated or lost in AD and AD+LBP cases (Supp. Fig. 3f,h)." The lack of associations could also be due to the small sample size for AD and AD+LBP cases. Also, for equal comparison, please include control samples in Supp Fig. 3f.

- Fig. 3i: The correlations for the PD comparisons seem to be driven primary by a cluster with minimal pigmented neuron loss. This should be mentioned in text

- Line 601: "This correlation further supports the potential of SAA as a reliable biomarker for pathology progression and staging in clinical and neuropathological settings." The authors should clarify that the described correlations are for postmortem tissue SAA, which have important neuropathological applications, but that CSF SAA is most often used in clinical settings and tend to be more sensitive when neocortical LBP is present.

Version 1:

Reviewer comments:

Reviewer #1

(Remarks to the Author)
All my concerns are addressed. I have no more questions.

Reviewer #2

(Remarks to the Author)
Summary: The revised manuscript addresses many of the concerns raised in the initial round of review and now incorporates relevant comparator studies that were previously omitted. The central conclusions and overall level of supporting evidence remain largely unchanged. As noted previously, the study has several strengths, particularly the comparison of non-PD neuropathological changes and the use of complementary assays in parallel tissue samples. However, several key issues would benefit from further clarification or revision.

Major Points:
1. While the other proteomic and transcriptomic studies from PD substantia nigra are now cited, it would be useful to include those in the introduction, to review what previous literature has shown, so novel findings of the current study can be highlighted.
2. The authors use Supplementary Fig. 5 to argue that there is no clustering by post-mortem interval (PMI), age, or DV200. However, there appears to be visible clustering, particularly by PMI across MDS2. Based on this analysis, the effect of PMI seems comparable to disease group, both of which are confounded by each other since different diseases/PMIs were obtained from different brain banks.
3. The additional C1QC staining provided in the response raises further concerns regarding antibody specificity. The near-complete overlap between C1QC and TH signal suggests the possibility of fluorescence bleed-through or channel cross-talk. This makes it difficult to interpret the specificity of the C1QC signal.

Version 2:

Reviewer comments:

Reviewer #2

(Remarks to the Author)
Reviewer concerns have been adequately addressed.

Open Access This Peer Review File is licensed under a Creative Commons Attribution 4.0 International License, which permits use, sharing, adaptation, distribution and reproduction in any medium or format, as long as you give appropriate credit to the original author(s) and the source, provide a link to the Creative Commons license, and indicate if changes were made.
In cases where reviewers are anonymous, credit should be given to 'Anonymous Referee' and the source.
The images or other third party material in this Peer Review File are included in the article's Creative Commons license, unless indicated otherwise in a credit line to the material. If material is not included in the article's Creative Commons license and your intended use is not permitted by statutory regulation or exceeds the permitted use, you will need to obtain permission directly from the copyright holder.
To view a copy of this license, visit https://creativecommons.org/licenses/by/4.0/

Reviewer #1:

We thank the reviewers for their thoughtful comments, which we believe have substantially strengthened the manuscript.

The authors applied a multi-omic spatial profiling approach, integrating transcriptomics, proteomics, and α-synuclein seed amplification assays (SAA), to post-mortem midbrain tissue from neurologically unaffected individuals, iLBD cases, patients with PD, AD, or AD+LBP. This is a very interesting and comprehensive study, which could be a good resource for the field. Following are my comments:

1. The correlations between pigmented neuron counts and both SAA AUC and Braak stage were attenuated or lost in AD and AD+LBP cases. It will be interesting to evaluate the extend of tau and Abeta pathology in all the cases examined, particularly the AD cases. Could the loss of correlation be attributed to the existence of AD pathology?

Response: In our material, dopaminergic neuron counts in the substantia nigra were not reduced in AD or AD+LBP compared with controls, indicating a limited dynamic range for neuron-loss–based correlations in these groups. Importantly, AD and AD+LBP cases uniformly exhibited advanced AD neuropathology, with high tau Braak stages (predominantly stages 5–6) and advanced amyloid pathology (**Fig. 1a, Table 1**), resulting in minimal within-group variability of tau/Aβ burden. This limits the ability of tau or amyloid pathology per se to explain the observed loss of correlation.

To formally address the significance of tau/Aβ, we leveraged the available case-level tau and Aβ staging data and performed multivariable linear regression analyses with pigmented neuron counts in the substantia nigra pars compacta as the outcome variable (l. 393 ff). In the primary model (n = 27 cases), predictors included SAA median AUC, Braak tau stage, Aβ stage, age, and PMI. In this model, SAA median AUC remained a strong independent predictor of pigmented neuron counts ($\beta = -1.0 \times 10^{-4}$, $p = 0.005$), whereas Braak tau stage was not significantly associated with neuron counts ($p = 0.88$). Aβ stage showed a small but significant independent association with reduced pigmented neuron counts (p values ranging from 0.007 to 0.03 across Aβ stages). Age and PMI were not significant covariates. The overall model demonstrated good fit (adjusted $R^2 = 0.73$; F-test $p = 6.6 \times 10^{-5}$). To test whether tau pathology modified the relationship between αSyn seeding activity and nigral neuron loss, we additionally fit an interaction model including an SAA AUC × Braak tau term. While the interaction term did not reach statistical significance ($p = 0.18$), its directionality was consistent with attenuation of the SAA–neuron relationship at higher tau stages. Given the limited sample size and restricted dynamic range of nigral neuron loss in AD-spectrum cases, this analysis was interpreted cautiously. Together, these analyses indicate that while αSyn seeding activity remains strongly associated with nigral neurodegeneration, Aβ pathology contributes independently to neuronal loss and likely underlies, at least in part, the reduced correlation observed in AD and AD+LBP cases. These findings support a disease-context–dependent decoupling of αSyn pathology and nigral neurodegeneration in the presence of substantial AD co-pathology.

2. 20 DEGs were found in the AD+LBP vs. Ctrl comparison. 2 DEGs in AD vs

controls comparation. This data suggests that LBP in AD further affect the gene expression compared with AD only. However, no DEGs were found in the comparison of AD cases with and without LBP. This is somehow confusing. Could this be due to the very limited number of cases used in this study?

Response: We agree that this pattern requires clarification. When considered together, the differential expression results indicate that disease context, rather than the mere presence of LBP, is the primary driver of transcriptional changes in the substantia nigra. This is supported by the strong transcriptional signal observed in PD vs. control (127 DEGs), compared with a more modest signal in AD+LBP vs. control (20 DEGs) and minimal changes in AD vs. control (2 DEGs). These findings suggest that while LBP can contribute to gene expression changes, its molecular impact is highly context dependent. In a PD disease context, LBP is associated with substantial transcriptional remodeling, whereas in AD, LBP alone appears insufficient to induce a comparable molecular response and cell death in the substantia nigra. The absence of DEGs in the comparison between AD cases with and without LBP further supports this interpretation, indicating that the addition of LBP does not markedly alter the transcriptional landscape beyond changes already present in AD. We acknowledge that the AD with vs. without LBP comparison is underpowered due to limited sample size, which may reduce sensitivity to detect subtle effects. However, the overall pattern across contrasts suggests that AD pathology may attenuate or constrain LBP-associated transcriptional changes in this region rather than amplify them. While the AD vs AD+LBP comparison may be underpowered to detect subtle transcriptional differences, the overall pattern across contrasts indicates that LBP-associated molecular remodeling is substantially stronger in the PD disease context. We have clarified this interpretation in the revised manuscript to emphasize that the molecular consequences of LBP are strongly modulated by disease context, with PD representing a fundamentally distinct biological environment from AD (l. 650 ff).

3. The authors need to verify at least some of the genes identified by spatial transcriptomics using immunofluorescent staining or in situ hybridization.

Response: To validate spatial transcriptomic findings using orthogonal approaches, we performed IHC analyses for two representative genes identified in the spatial transcriptomics dataset. Consistent with the ST results, tyrosine hydroxylase (TH) showed a clear reduction in the substantia nigra, confirming the expected loss of dopaminergic neurons. In addition, we examined neuropeptide Y (NPY), which showed an increased signal in the ST data. While NPY immunoreactivity exhibited a similar trend by immunohistochemistry, this difference did not reach statistical significance (**Supp. Fig. 6**). Differences in sensitivity and dynamic range between ST and protein detection IHC are expected (**Fig. 4a**), as these approaches capture distinct molecular readouts (mRNA abundance versus protein levels) and differ in spatial resolution, quantification, and signal normalization. Moreover, post-transcriptional regulation and protein turnover can decouple mRNA and protein abundance.

Reviewer #2:

Summary: Rumpf and colleagues present a study of 10x Visium spatial transcriptomics and proteomics in the substantia nigra of control, incidental Lewy body disease, Parkinson's disease, Alzheimer's disease, and Alzheimer's disease with Lewy body pathology. This type of profiling study holds great

promise to identify novel pathways perturbed in Parkinson's disease. A particular strength of the study is the inclusion of several comparator groups for PD, beyond simply including matched controls. Several complementary assays, such as seed amplification, proximity ligation, and immunofluorescence assays are employed to aid interpretation. Despite these strengths, there are several major limitations of the study which diminish the ability to interpret results, and several similar published studies detract from the likely impact. Major and minor points are listed below.

Major Points:

1. There are some overall issues with cohort design that make subsequent interpretation difficult. 1) The number of cases overall is low (32 cases total).

Response: We agree with the reviewer that the overall cohort size is limited, which is an inherent constraint of post-mortem human midbrain studies combining spatial transcriptomics, spatial proteomics, and multiple orthogonal validation assays. Access to well-characterized substantia nigra tissue with short post-mortem intervals, high RNA integrity, and detailed neuropathological staging is extremely limited, particularly for prodromal conditions such as iLBD and for AD cases with defined midbrain LB co-pathology. Importantly, the primary aim of this study was not to provide population-level estimates or exhaustive catalogues of disease-associated changes, but to perform a discovery-driven, spatially resolved analysis across carefully selected and neuropathologically defined comparator groups, including PD, iLBD, AD, and AD+LBP. The inclusion of multiple disease contexts, rather than reliance on a single control group, was intended to increase interpretability and specificity of observed molecular changes, despite the limited absolute sample size. To mitigate sample size constraints, we employed several strategies: (i) strict case selection and matching for age, APOE genotype (in the AD context) and sex, (ii) pseudo bulk DEG analyses at the spatial cluster level to reduce noise, (iii) conservative statistical thresholds with multiple-testing correction, and (iv) validation of key findings using independent modalities. Furthermore, we explicitly avoid overinterpretation of negative or underpowered contrasts and frame conclusions accordingly. We now more clearly acknowledge cohort size as a limitation in the Discussion (l. 783 ff) and emphasize that the findings should be interpreted as hypothesis-generating and mechanistically informative rather than definitive.

2) Tissues for AD cases were obtained from a different brain bank with much different processing.

Response: To address this concern, we performed additional analyses. For ST, multidimensional scaling analyses demonstrated that samples did not cluster according to brain bank, age, RNA quality (DV200), or post-mortem interval, indicating that these variables did not drive the observed transcriptional patterns (**Supp. Fig. 5**). Similarly, for proteomics, despite differences in fixation times and post-mortem intervals, samples yielded highly comparable proteomic coverage across cohorts (~4.000 proteins identified per sample, **Supp. Fig. 8**), arguing against systematic protein degradation or site-specific loss of signal. The number of quantified proteins was similar across conditions, and proteomic profiles did not segregate by brain bank or diagnosis but rather by anatomical compartment (SNpc vs Fibers) (**Supp. Fig. 9c**). Together, these analyses indicate that potential differences between brain banks did

not introduce detectable confounding effects in either the spatial transcriptomic or proteomic datasets. We have clarified this point and added the relevant quality control and clustering analyses to the revised manuscript (l. 409 ff & 474 ff)

3) High post-mortem intervals (~24 hours for main comparators) and fixation times (28 days+) could contribute to poor tissue quality. Were these assessed by RIN score or other QC metrics?

Response: RNA quality was carefully assessed and controlled for in this study. Because all analyses were performed on formalin-fixed paraffin-embedded (FFPE) tissue, conventional RNA integrity metrics such as RIN are not applicable. Instead, RNA quality was evaluated using the DV200 metric, which quantifies the percentage of RNA fragments longer than 200 nucleotides and is the recommended quality control measure for FFPE-based transcriptomic analyses, including the 10x Visium FFPE platform. In accordance with the manufacturer's recommendations, only samples exceeding the recommended DV200 cutoff of 30% were included in the ST analyses. All cases used in the study met this criterion. In addition, sequencing quality metrics (e.g., median genes per spot and read depth) were monitored and did not show systematic differences between diagnostic groups, indicating comparable RNA quality across cohorts despite differences in post-mortem interval and fixation time (**Supp. Fig. 4**) (l. 409 ff).

2. Spatial transcriptomics is a relatively recent technology that has enabled new insights into neurodegenerative disease. However, several groups have already published on transcriptomic shifts in Parkinson's disease. In particular, two studies have used the same technology (10x Visium) in the substantia nigra of Parkinson's disease and control tissues: PMID: 40759663, bioRxiv: https://doi.org/10.1101/2024.07.28.605055. Neither of these studies are cited in the current manuscript.

Response: We thank the reviewer for pointing out these important recent studies and apologize for not citing them in the original submission. We have now cited both the published study[1] and the recent bioRxiv preprint[2] in the revised manuscript. While these studies also employ 10x Visium ST in the substantia nigra of PD and Ctrl tissue, our work differs in several key aspects. First, we include multiple neuropathologically defined comparator groups beyond PD and controls, including iLBD, AD, and AD+LBP, enabling a disease-context–specific interpretation of αSyn–associated changes. Second, we integrate spatial transcriptomics with spatial proteomics, αSyn SAA, proximity ligation assays, and immunofluorescence, providing orthogonal validation and mechanistic insight that extend beyond transcript-level observations alone. Third, our study explicitly addresses early and prodromal disease stages and disentangles Lewy body–associated changes from αSyn–independent processes, which were not the primary focus of the prior studies. We have added these citations and clarified the relationship of our findings to the existing literature in the Discussion (l. 683 ff).

3. Major findings from the transcriptomics data seem to be largely related to the difference in cell type abundance, particularly the loss of dopaminergic neurons in the substantia nigra. The utility of differential gene expression across brain regions with known disparate cell type abundances is unclear.

Response: We agree that cell type composition, particularly dopaminergic neuron loss in the substantia nigra must be considered when interpreting transcriptomic data. We therefore distinguish between a global and a domain-specific transcriptomic perspective. We first present a cluster-agnostic, global analysis of dopaminergic marker expression (*TH, SLC6A3, SLC18A2*; **Fig. 2a**), which captures the expected progressive loss of dopaminergic neurons along the PD trajectory and independently validates our histological neuron counts. Importantly, downstream differential expression analyses were performed in a domain-specific manner using spatial transcriptomics (10x Visium) combined with spatially informed clustering (Bayesian Analytics for Spatial Segmentation; BASS),[3] which preserves anatomical context and restricts comparisons to spatially comparable tissue domains rather than entire homogenized regions. BASS was specifically created to jointly model spatial clusters across individuals from different groups, an important modality that is currently not covered by most spatial clustering algorithms. As such, spatial dependence is explicitly modeled by group, stabilizing sparse signals from one individual (by incorporating Bayesian priors). This leads to greatly improved robustness to heterogeneity across samples. Consistent with this approach, canonical dopaminergic markers did not emerge as differentially expressed in well-defined substantia nigra clusters when comparing PD and control cases (**Fig. 3c**), indicating that the identified DEGs are not simply driven by dopaminergic neuron depletion:

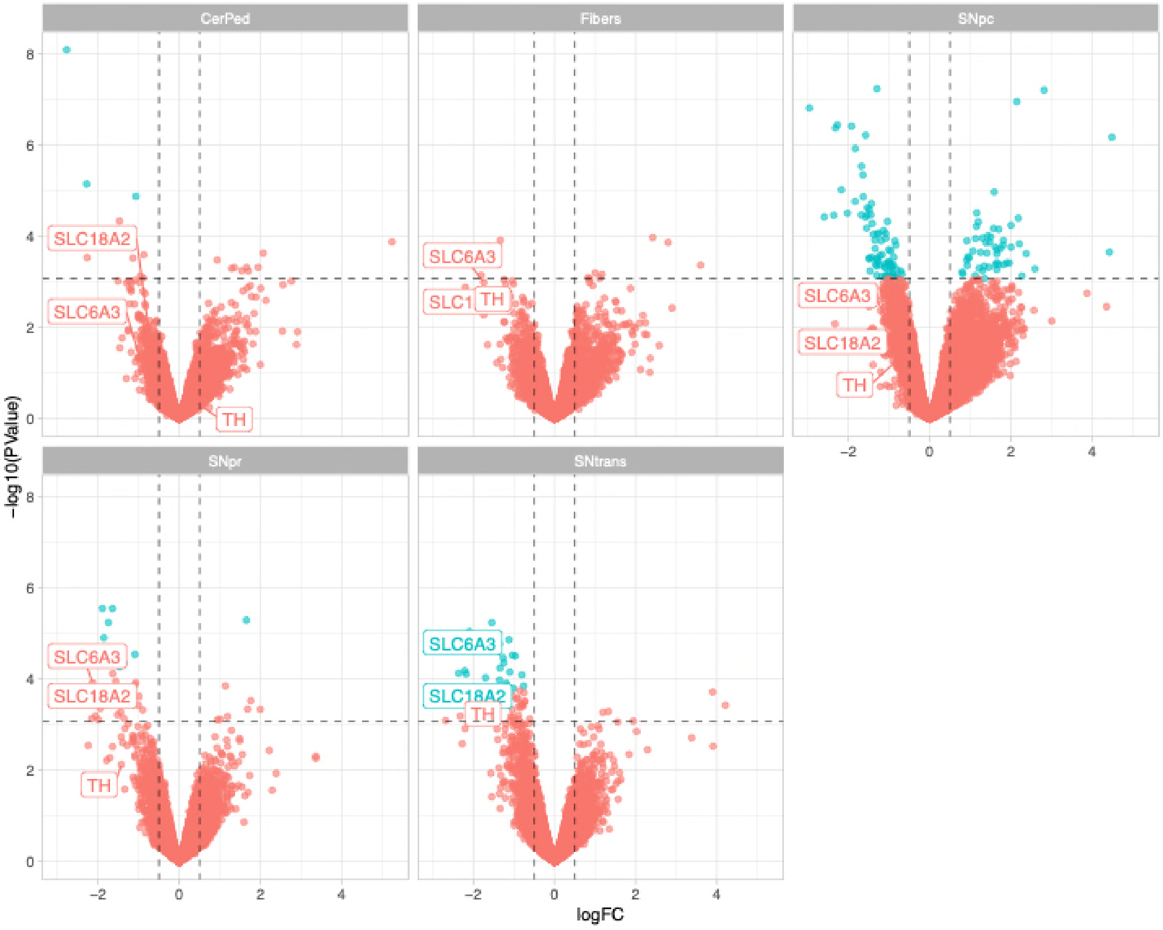

Volcano plots illustrating no significant domain specific change in the abundance of typical DA marker genes.

Only in the transitional SN zone (cluster "SNtrans") did we observe modest changes in dopaminergic markers, likely reflecting boundary effects in cluster definition. Overall, the use of spatial transcriptomics, spatially informed clustering, and conservative

statistical thresholds supports the interpretability of our analyses while accounting for cellular heterogeneity in the substantia nigra.

4. Proteomics is a less novel technology, and many groups have published proteomic studies of the Parkinson's disease brain. These three studies are in the substantia nigra, and one of them even employs a similar laser capture strategy: PMID: 36423813, 32132230, 18275612. Again, these studies are not cited in the current manuscript.

Response: We thank the reviewer for highlighting these important prior proteomic studies of the PD substantia nigra. We have now cited the indicated studies[4–6] in the revised manuscript. Proteomic analyses of the PD brain, including studies employing laser capture microdissection of the substantia nigra, have provided valuable insights into disease-associated molecular changes. Our work is intended to extend rather than duplicate these efforts. In contrast to prior studies focusing primarily on PD–control comparisons, our proteomic analysis is explicitly integrated with ST and αSyn SAAs across multiple neuropathologically defined comparator groups, including iLBD and AD with or without LBP. This design enables disease-context–specific interpretation of proteomic changes and facilitates direct linkage between protein-level alterations, spatial gene expression patterns, and αSyn pathology. We have added the relevant citations and clarified the relationship between our findings and previous proteomic studies in the Discussion (l. 692 ff).

5. The purification of α-synuclein is not well-described. It is insufficient to refer to a previous paper. What is the construct being purified? It is noted that it is "wild-type α-synuclein," however, the purification is through a Ni-NTA column, which suggests the protein is His-tagged. Further details are required.

Response: We have now expanded the Methods section to provide additional details on the αSyn construct and purification procedure. Specifically, we clarify the expression vector used, including the presence of an N-terminal His-tag enabling purification by Ni-NTA affinity chromatography, and provide the appropriate reference where the construct and purification protocol are described in full (l. 157 ff).

6. Lines 222-224: "Of the 54,580 probes included in the FFPE Visium v2 assay, 8,652 were removed as recommended by the 10X QC department (personal communication)." Please provide additional information about why these probes were removed.

Response: The probes were removed due to a manufacturing-related batch effect identified in the 10x Visium FFPE v2 assay. In our initial analyses including all probes, we observed a biologically implausible batch effect associated with two different Visium v2 manufacturing lots, which exceeded group-specific differences in differential expression. Because samples from both brain banks were distributed across the two lots, this effect could be unambiguously attributed to the assay rather than biological variables. We reported this issue to the 10x Genomics quality control team, who performed an internal investigation and confirmed that a subset of probes was affected by manufacturing inconsistencies. Based on their recommendation and a list of probes to exclude, we excluded 8.652 affected probes from downstream analyses. After probe

removal, batch effects were no longer detectable and differential expression results were biologically plausible and consistent across cohorts.

7. Fig. 1f: Several control samples show some positivity in the SAA, but this is not discussed in the results section.

Response: As described in the Methods, SAA positivity was not determined by signal amplitude or area under the curve (AUC) alone, but by a stringent replicate-based criterion consistent with standard RT-QuIC practice.[7–9] In this assay, occasional low-level or sporadic signal increases can arise from self-aggregation of the recombinant protein, and therefore interpretation relies on concordance across multiple technical replicates. In diagnostic settings, samples are considered positive only when the majority of replicates exceed the predefined threshold, whereas isolated or inconsistent replicate positivity is classified as negative or inconclusive. We have clarified this point in the Discussion (l 631 ff) to explain the apparent low-level signal in some control samples and to emphasize the importance of replicate-based interpretation rather than reliance on AUC alone.

8. Lines 442-444: "This notion was further corroborated by comparing the group-wise log fold-changes of DEGs with the Pearson's correlation coefficient of gene counts to the number of pigmented cells." Does this suggest that most of the differential gene expression is due to a loss of pigmented neurons?

Response: We clarify that the analysis shown in Fig. 3i does not imply that most differential gene expression is driven by loss of pigmented (dopaminergic) neurons. This analysis relates group-wise log fold-changes of DEGs to the spatial correlation between gene expression and pigmented neuron counts, which reflects co-variation but does not establish causality. DEGs span a broad range of correlation coefficients, including genes with weak or near-zero association with pigmented neuron abundance, indicating that differential expression is not uniformly linked to neuron loss. Moreover, genes with similar correlations can show divergent fold-changes across disease contrasts, and both positive and negative correlations are observed, consistent with contributions from multiple cell types and disease-related regulatory processes rather than a simple depletion effect. The spatially informed clustering strategy further limits the influence of bulk cell loss on downstream analyses. We therefore interpret this analysis as indicating that dopaminergic neuron vulnerability contributes to, but does not fully explain, the observed transcriptional changes in PD and iLBD, with a substantially weaker contribution in AD with LBP and have clarified this more adequately in the updated manuscript.

9. Supplementary Fig. 7: This is noted in the text as a pathway analysis, but it appears to be a cell type deconvolution.

Response: Corrected – it is not a pathway analysis but indeed a cell type deconvolution analysis.

10. It is surprising that SNCA, encoding α-synuclein did not show a significant difference in the PD vs. CTRL proteomic comparison. Do the authors think this is due to the extraction method which may have left insoluble proteins behind? Or a detection limit of the method? Or due to the difference in cell abundance in this region?

Response: We thank the reviewer for this important question. Although αSyn aggregation is a pathological hallmark of PD, changes in total αSyn abundance detectable by unbiased proteomics are not consistently observed. Several studies have reported that soluble αSyn levels in post-mortem brain tissue are similar between PD cases and controls despite clear LBP, suggesting that pathological changes are driven primarily by shifts in aggregation state and solubility rather than overall protein abundance.[10] Furthermore, pathological αSyn is typically sequestered into insoluble aggregates and LBs, which are poorly extracted by standard proteomic workflows and may therefore be under-represented in bulk protein measurements.[11] We emphasize this in our revised manuscript (l. 636 ff).

Importantly, in our dataset, αSyn was robustly and reproducibly quantified at the protein level, with good peptide coverage and signal intensities well within the measurable dynamic range of the assay. αSyn protein abundance did not differ significantly between PD and control samples, but showed enrichment in substantia nigra subclusters, indicating spatial heterogeneity rather than global changes in abundance:

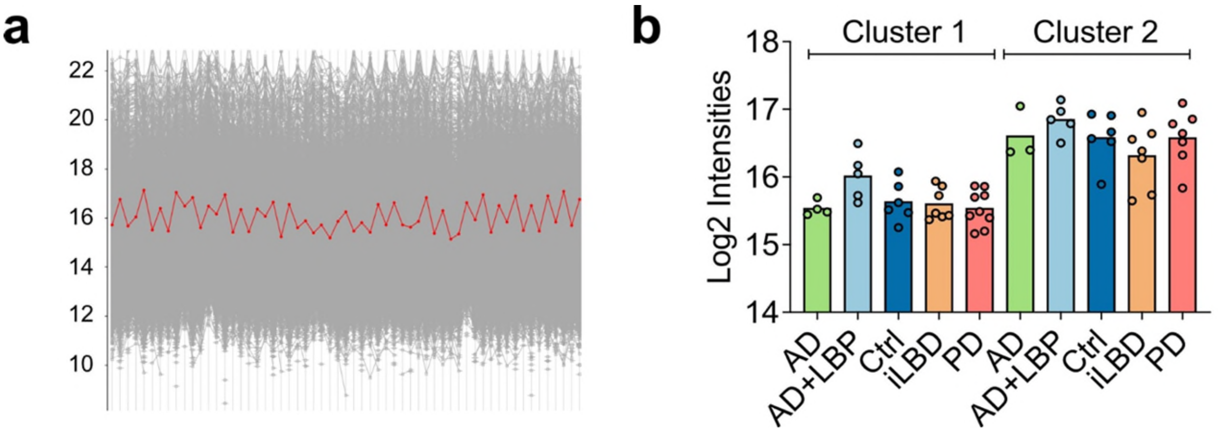

a, Graph illustrating the embedding of aSyn protein measurements (red line) relative to all measured proteins (grey background lines) indicating that the abundance of aSyn was well in the range of the MS detection limits. **b,** Depicted are Log2-transformed LFQ Intensities. Graphs showing that aSyn abundance is similar across all groups but is enriched in Cluster 2 (SNc) compared with Cluster 1 (fibres).

While we did not specifically employ extraction protocols optimized for insoluble αSyn species (e.g., high-molar chaotropic buffers), and therefore cannot exclude selective loss of aggregated proteoforms, the reproducible detection of αSyn across samples supports the conclusion that the absence of a PD–control difference reflects biology rather than a technical detection limit. Taken together, these observations suggest that αSyn pathology in PD is better captured by assays sensitive to aggregation state and seeding activity than by measurements of total protein abundance, consistent with our proteomic findings.

11. C1QC staining in both human tissue and mouse models appears to be largely in neuronal cell bodies. Is this the expected pattern?

Response: We thank the reviewer for this important observation. While C1q is predominantly produced by microglia in the central nervous system, its immunoreactivity is frequently observed in close association with neuronal structures. C1q is known to bind neuronal membranes and synapses and to accumulate on

stressed or degenerating neurons, particularly in disease contexts involving synaptic remodeling or neurodegeneration. Thus, neuronal-associated staining likely reflects complement deposition on neuronal membranes rather than neuronal synthesis. In addition, C1q-positive material may be detected within or adjacent to neuronal somata due to complement deposition, microglial–neuronal apposition, or ongoing phagocytic processes. These patterns have been described in multiple neurodegenerative and inflammatory contexts and do not necessarily imply neuronal synthesis of C1q. We have clarified this point in the manuscript (l. 760 ff) emphasizing the signal as C1QC immunoreactivity associated with neuronal cell bodies rather than neuronal expression per se.

Have the authors identified a method to validate the specificity of this staining?

Response: Yes. To validate staining specificity, we included negative control sections in which the primary antibody was omitted; under these conditions, no signal was detected. Additionally, we have employed a different C1QC antibody, which showed a similar staining pattern to the antibody employed in our manuscript:

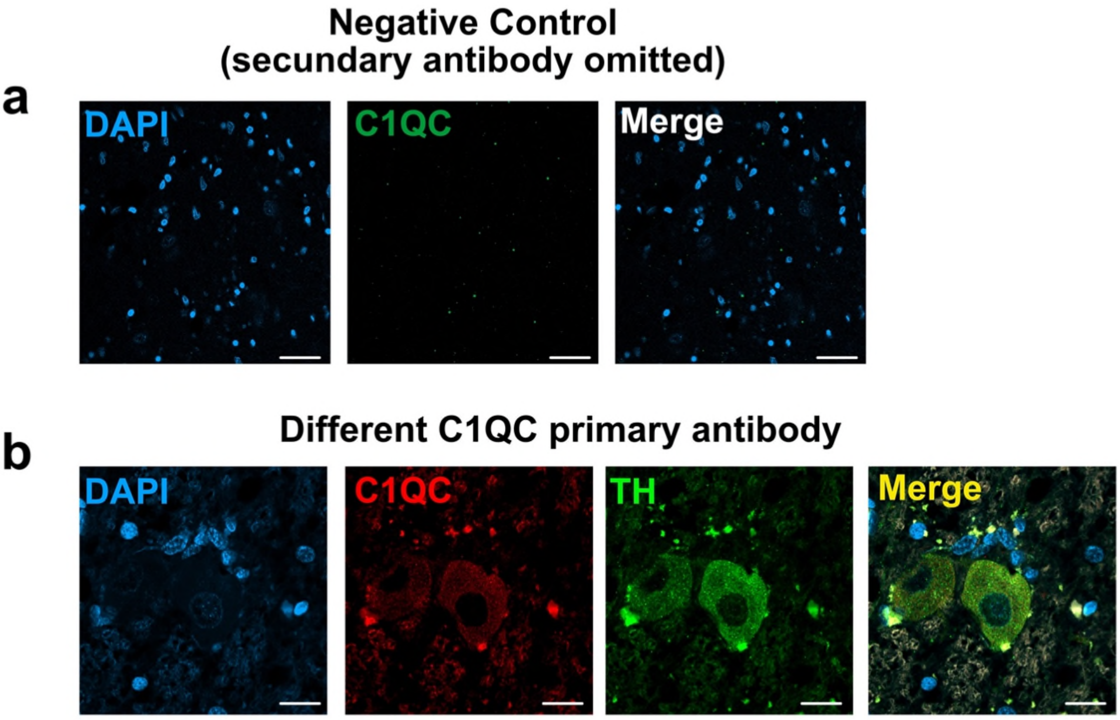

a, Representative photomicrographs of substantia nigra sections processed for immunohistochemistry without incubation with the primary C1QC antibody (Thermo Fisher Scientific; PA5-106648), demonstrating absence of detectable signal and confirming staining specificity. **b**, Representative photomicrographs of substantia nigra sections processed for immunohistochemistry with the primary C1QC antibody (Thermo Fisher Scientific; PA5-51583).

12. It is postulated by the authors that "microglial phagocytosis of GABAergic postsynapses is enhanced, leading to a loss of inhibitory input onto SNpc dopaminergic neurons and, as a potential consequence, disinhibition of these cells." However, to support this hypothesis, there would need to be evidence of an overall decrease of GABAergic terminals/dopaminergic neuron.

Response: To directly assess this consequence, we stained midbrain sections from Ctrl, iLBD, and PD cases for the GABAergic terminal marker GAD67 and TH to label DAergic neurons and their afferents. Consistent with a functional consequence of C1QC-mediated synapse stripping, we observed a reduction in GAD67-positive fibers in iLBD and PD compared with Ctrl cases (**Fig. 6f,h**). IMARIS-based 3D reconstruction further suggested a decrease in TH-/GAD67 double-positive structures (**Fig. 6g**). We have clarified this information in our revised manuscript (l. 588 ff & 768 ff)

Minor Points:

1. References 16 and 17 are the same paper.

Response: Corrected

2. Supplementary Fig. 3: No legend is provided for the colors of the dots.

Response: A legend explaining the color coding has now been added to **Supp. Fig. 3** in the revised manuscript.

3. Supplementary Fig. 3: There are inconsistent nomenclatures and fonts. Some correlations are reported with an R^2, while some are reported as r. A comma is used in panel c.

Response: The use of r versus R^2 reflects the different statistical approaches applied depending on the data type and distribution. Non-parametric correlations (Spearman's r) were used for variables that are ordinal or discrete, such as Braak stage and pigmented neuron counts, whereas parametric linear regression was applied where appropriate, and model fit is summarized using R^2 (as mentioned in the figure legend). We corrected typographical errors, including the comma in panel c.

4. The directionality for the volcano plots is not always clear. For example, in Fig. 4e, the comparison is noted as iLBD vs. Control, but apparently proteins enriched in iLBD are on the right.

Response: Across all volcano plots, fold-change directionality is defined relative to the first group listed in the comparison title. Thus, in **Fig. 4e** (iLBD vs Ctrl), proteins enriched in iLBD are shown on the right (positive log fold-change), whereas proteins reduced in iLBD relative to controls appear on the left. For example, C1QC is increased in iLBD, while GPRIN1 is decreased in iLBD. To avoid confusion, we have clarified this convention in the figure legends.

5. Please note the log scale for volcano plots. They are noted as "logFC." Presumably, this is Log2 fold change, but this is not noted.

Response: We thank the reviewer for pointing this out. As correctly noted, all fold-change values shown in the volcano plots represent $\log_2$ fold changes. We have corrected the labeling throughout the manuscript and figure legends to explicitly state "$\log_2$FC" to avoid any ambiguity.

Reviewer #3:

In this manuscript, Rumpf, Strubing et al. examine the cellular and molecular changes that occur in the substantia nigra in different synucleinopathies. They utilize multiple techniques to extract spatial histopathological, transcriptomic, proteomic information from consecutive FFPE sections of patients with either incidental Lewy body disease (iLBD), Parkinson's disease (PD), Alzheimer's (AD), AD+LBD, and controls. From this rich collective dataset, they report specific signatures associated with each disease type, perhaps the most significant being the upregulation and enrichment of complement C1q in the SNpc of iLBD subjects. Surprisingly, this was not observed in the SNpc of subjects with AD+LBP despite these samples scoring having similar Braak LB staging and seed amplification assay (SAA) status. In follow up studies, they show increased C1q localization to ventral tier dopamine neurons of iLBD (but not AD+LBD), and C1q interaction with the GABAergic postsynaptic scaffold protein Gephyrin within CD68+ puncta, suggesting that they are engulfed in microglial lysosomes. The authors conclude that this neuroinflammatory response represents a unique and novel feature that distinguishes iLBD from AD+LBP, and the intriguing possibility that this occurs in the absence of frank Lewy pathology as this is not observed in AD+LBD. These findings therefore address the central question of whether AD+LBP and iLBD/PD follow distinct pathogenetic pathways and introduce a powerful methodological roadmap for examining this. On the other hand, there are a number of gaps in the data and interpretation in this current manuscript that need to be addressed in order to more fully substantiate the main claims made.

Main points:

- The observation that C1q is upregulated in SNpc of iLBD vs AD+LBP is an important one with potentially profound mechanistic and clinical implications. However, a detailed comparison with C1q status in the PD samples is crucial to establish whether this remains elevated or is transient. Either outcome would be significant but would provide important insights into whether iLBD/PD represents a distinct disease axis compared to AD+LBD or that C1q is a specific marker of only iLBD.

Response: We have directly addressed this question by staining additional sections from PD cases (n = 4). Our results showed no significant increase in C1QC in these cases (**Fig. 5g,h**), suggesting that the elevation in C1QC may be associated with early pathological changes and may be less prominent at later disease stages. We have discussed the implications of this finding—particularly the possibility of a therapeutic window of opportunity—in the revised manuscript (l. 744 ff).

The data in question should already be contained in Supp Fig 6, but none of the targets are labeled in this figure. Since the complement pathway is being invoked as being specific to iLBD (and perhaps PD), levels of other complement components should also be highlighted as they have been widely described in the context of AD. For example, was staining or PLA with C3 performed?

Response: We have updated the revised figure to explicitly highlight complement-associated targets, indicating both significant and non-significant findings across all

examined contrasts (**Supp. Fig. 10**). In addition to core microglial markers, we annotated a broad range of complement pathway components that have been widely implicated in neurodegenerative disease, including AD. These targets include TYROBP; C1Q complex members (C1QA, C1QB, C1QC); upstream and downstream complement factors (C1R, C1S, C2, C3, C4A, C4B, C5, C5AR1); complement regulators (CD55); and complement-related immune receptors and signaling molecules (ITGAM, ITGB2, FCGR2B, AXL). With respect to C3 specifically, we additionally performed immunohistochemical analyses to quantify C3 abundance in iLBD versus PD or Ctrl cases. These analyses did not reveal a statistically significant difference, although a trend was observed (**Supp. Fig. 14**).

Similarly, was C1q elevated in the neocortex or amygdala of AD+LBD?

Response: We thank the reviewer for this question. We did not assess C1q levels in the neocortex or amygdala, as cortical tissue was not available for the present study. Accordingly, our conclusions regarding C1QC are restricted to the substantia nigra, and we now explicitly note this limitation in the manuscript (l. 791 ff).

Given the very significant differences in PMI and fixation time between AD and non-AD samples, these could also serve as an internal control for validating the integrity of the targets across samples from the two sites.

Response: Differences in post-mortem interval and fixation time were formally addressed in the multivariable regression analyses described above, in which post-mortem interval and age were included as covariates when modeling key molecular and histological outcomes (including pigmented neuron counts and SAA). In these models, neither PMI nor age significantly contributed to the observed group differences, and inclusion of these variables did not alter the main results. Consistent with this, RNA quality metrics (DV200) and molecular readouts were preserved in AD cases despite longer PMI and fixation times.

- LB dementia (DLB) also features dopamine neuron loss but discussion about it is notably absent from the study. Since one of the major conclusions is that SNpc neuron loss/C1q activation is not correlated with synucleinopathy, please provide a rationale that the DLB samples were not examined.

Response: The absence of a dedicated DLB cohort in the present study reflects cohort availability and study scope rather than a conceptual exclusion of DLB from the biological framework. Our study was specifically designed to compare PD with iLBD and with AD cases with and without Lewy body co-pathology, thereby enabling us to disentangle α-synuclein–associated changes from AD-related processes within a well-defined anatomical context. Inclusion of DLB would require a sufficiently powered and neuropathologically homogeneous cohort with detailed staging of both cortical and brainstem pathology, which was not available within the constraints of the present study. Moreover, DLB is characterized by substantial cortical Lewy pathology and clinical dementia, raising additional variables beyond the midbrain-focused questions addressed here. Importantly, our conclusions are restricted to the disease contexts examined and are not intended to generalize to all synucleinopathies, including DLB. We now explicitly acknowledge this limitation in the Discussion and note that extending the present spatial multi-omics approach to DLB represents an important direction for future work (l. 796 ff).

- Line 517: "Region-Specific Upregulation of C1QC in iLBD Suggests Early, αSyn-Independent Inflammatory Signaling." This is a bold and exciting hypothesis and based largely on the observation here that LBP, when present in AD, does not present with this signature nor dopamine cell loss. However, an important caveat is that LBP is present in iLBD - thus, an equally valid interpretation is that LBP is not sufficient (but possibly still necessary) for this phenomenon.

Response: We clarify our operational definition of iLBD: In line with prior work,[12] iLBD cases in this study were defined as Lewy body Braak stage ≤ 2, a stage at which LBP is restricted to lower brainstem regions and is absent from the substantia nigra pars compacta. Thus, the region-specific upregulation of C1QC observed in the substantia nigra in iLBD occurs in the absence of local Lewy body pathology and precedes overt dopaminergic neuron loss which is also shown in **Fig. 1c,d.** In contrast, AD cases with LB co-pathology exhibit higher Braak stages with established LBP yet do not show this C1QC signature. Together, these observations support the interpretation that early C1QC upregulation in the substantia nigra reflects inflammatory signaling that is not primarily driven by visible αSyn pathology per se. We have revised the text to clarify this distinction and to avoid overstatement of αSyn independence (l. 747 ff).

- Fig 6: The observation that Gephyrin colocalizes to microglial CD68+ lysosomes is interesting. However, the Gephyrin+ objects appear to be much larger than the CD68 puncta, which makes it hard to reconcile as inside the lysosomal lumen.

Response: We have replaced the representative images in **Figure 6** with higher-quality examples that more clearly illustrate the spatial relationship between gephyrin and CD68-positive microglial lysosomal structures. The revised images better demonstrate the association of gephyrin signal with CD68-positive compartments, addressing the previous concern regarding the apparent size discrepancy between gephyrin-positive objects and CD68 puncta.

Other points:

- The use of multiple spatial-omics (transcriptomics, proteomics), histological (IHC, PLA), and biochemical (SAA) approaches on consecutive tissue samples is a core strength of the manuscript. This should be emphasized by showing data from consecutive (or nearby) sections in Fig 1b-d so that readers can appreciate the correlation between these sources of data. The images currently shown do not appear to be taken from the same sample/subject.

Response: To better illustrate this workflow, we have adapted **Figure 1** to include a schematic outlining the use of consecutive tissue sections for the spatial transcriptomic, spatial proteomic, and biochemical assays. Conventional IHC and PLA experiments, however, were not performed on directly consecutive sections but instead were conducted on representative sections derived from the same cohort. As a result, these assays cannot be shown as directly consecutive sections in Figure 1. We have clarified this point in the figure legend to ensure transparency while still highlighting the complementary nature of these datasets.

- Line 382: "In contrast, these associations were attenuated or lost in AD and

AD+LBP cases (Supp. Fig. 3f,h)." The lack of associations could also be due to the small sample size for AD and AD+LBP cases. Also, for equal comparison, please include control samples in Supp Fig. 3f.

Response: We agree with the reviewer that the absence of significant associations in the AD and AD+LBP groups may, in part, reflect limited statistical power due to smaller sample sizes. We have clarified this limitation in the revised text and now explicitly frame these analyses as exploratory in the AD-spectrum cohorts. In addition, as suggested, we have included control samples in **Supp. Fig. 3f** to enable a more balanced comparison across disease groups.

- Fig. 3i: The correlations for the PD comparisons seem to be driven primary by a cluster with minimal pigmented neuron loss. This should be mentioned in text

Response: We thank the reviewer for this observation and would like to clarify that the apparent “cluster” in Fig. 3i does not represent a subset of spatial regions or tissue areas with minimal dopaminergic neuron loss. This analysis is restricted to a single spatial domain (SNpc) defined by BASS clustering, and each point represents an individual differentially expressed gene rather than a cellular or anatomical cluster. The x-axis shows the correlation between gene expression and pigmented neuron counts across spatial spots within this domain, while the y-axis reflects the group-wise $\log_2$ fold-change for each gene. The aggregation of points therefore reflects a group of genes with similar relationships to dopaminergic neuron abundance, not a subset of spots with preserved neurons. Importantly, the absence of such correlations in the AD+LBP comparison indicates that differential expression in this condition is largely independent of dopaminergic neuron loss.

- Line 601: "This correlation further supports the potential of SAA as a reliable biomarker for pathology progression and staging in clinical and neuropathological settings." The authors should clarify that the described correlations are for postmortem tissue SAA, which have important neuropathological applications, but that CSF SAA is most often used in clinical settings and tend to be more sensitive when neocortical LBP is present.

Response: We thank the reviewer for this important clarification. We agree that the correlations described in this study are based on α-synuclein seed amplification assays performed on postmortem tissue and are therefore most directly relevant to neuropathological staging and postmortem assessment of disease progression. We have revised the text to explicitly distinguish these applications from clinical CSF-based SAA, which is more commonly used in clinical settings and typically shows higher sensitivity in the presence of neocortical Lewy body pathology. This distinction is now clearly stated in the revised manuscript (l. 625 ff)

Additional References:

1. Ma, M. *et al.* The spatial landscape of glial pathology and T cell response in Parkinson’s disease substantia nigra. *Nat. Commun.* **16**, 7146 (2025).

2. Yoo, S. *et al.* Single-nucleus and spatial transcriptomic analysis identified molecular features of neuronal heterogeneity and distinct glial responses in Parkinson's disease. *bioRxiv* 2024.07.28.605055 (2024) doi:10.1101/2024.07.28.605055.

3. Li, Z. & Zhou, X. BASS: multi-scale and multi-sample analysis enables accurate cell type clustering and spatial domain detection in spatial transcriptomic studies. *Genome Biol.* **23**, 168 (2022).

4. Jang, Y. *et al.* Mass Spectrometry–Based Proteomics Analysis of Human Substantia Nigra From Parkinson's Disease Patients Identifies Multiple Pathways Potentially Involved in the Disease. *Mol. Cell. Proteom.* **22**, 100452 (2023).

5. Griesser, E. *et al.* Quantitative Profiling of the Human Substantia Nigra Proteome from Laser-capture Microdissected FFPE Tissue*. *Mol. Cell. Proteom.* **19**, 839–851 (2020).

6. Werner, C. J., Haussen, R. H., Mall, G. & Wolf, S. Proteome analysis of human substantia nigra in Parkinson's disease. *Proteome Sci.* **6**, 8 (2008).

7. Brockmann, K. *et al.* Quantification of cerebrospinal fluid α-synuclein seeds by endpoint dilution seed amplification assay in Parkinson's disease. *npj Park.'s Dis.* **12**, 5 (2025).

8. Mastenbroek, S. E. *et al.* Biological classification of memory clinic patients. *Brain* awaf411 (2025) doi:10.1093/brain/awaf411.

9. Rossi, M. *et al.* Comparison of Two α-Synuclein Seed Amplification Assays for Discrimination of Parkinson Disease and Atypical Parkinsonism. *Mov. Disord.* **40**, 2504–2509 (2025).

10. Quinn, J. G. *et al.* α-Synuclein mRNA and soluble α-synuclein protein levels in post-mortem brain from patients with Parkinson's disease, dementia with Lewy bodies, and Alzheimer's disease. *Brain Res.* **1459**, 71–80 (2012).

11. Srinivasan, E. *et al.* Alpha-Synuclein Aggregation in Parkinson's Disease. *Front. Med.* **8**, 736978 (2021).

12. Koeglsperger, T. *et al.* Neuropathology of incidental Lewy body & prodromal Parkinson's disease. *Mol. Neurodegener.* **18**, 32 (2023).

Reviewer #2 (Remarks to the Author):

Summary: The revised manuscript addresses many of the concerns raised in the initial round of review and now incorporates relevant comparator studies that were previously omitted. The central conclusions and overall level of supporting evidence remain largely unchanged. As noted previously, the study has several strengths, particularly the comparison of non-PD neuropathological changes and the use of complementary assays in parallel tissue samples. However, several key issues would benefit from further clarification or revision.

Major Points:

1. While the other proteomic and transcriptomic studies from PD substantia nigra are now cited, it would be useful to include those in the introduction, to review what previous literature has shown, so novel findings of the current study can be highlighted.

Response: We thank the reviewer for this helpful suggestion. We have added a paragraph to the Introduction (l. 84 ff) briefly summarizing prior transcriptomic and proteomic studies of the SN in PD. Specifically, we now reference recent single-cell and spatial transcriptomic studies that have identified cell type–specific and regionally resolved molecular alterations, as well as proteomic analyses that have characterized disease-associated protein signatures. This addition provides context for our study and highlights how our integrated spatial multiomic approach extends prior work by incorporating multiple neuropathologically defined disease states, including prodromal iLBD and AD-associated Lewy pathology.

2. The authors use Supplementary Fig. 5 to argue that there is no clustering by post-mortem interval (PMI), age, or DV200. However, there appears to be visible clustering, particularly by PMI across MDS2. Based on this analysis, the effect of PMI seems comparable to disease group, both of which are confounded by each other since different diseases/PMIs were obtained from different brain banks.

Response: We agree that a gradient along MDS2 is visible when samples are colored by PMI. However, as correctly noted by the reviewer, PMI is strongly correlated with Visium kit batch ($p = 1 \times 10^{-4}$), reflecting the use of two different Visium v2 reagent lots in this study. The original analysis already corrected for kit batch, but not PMI. We decided against including both variables simultaneously because variance inflation diagnostics indicated substantial shared explanatory variance between them (GVIF^(1/(2×df)): kit batch = 1.89; PMI = 1.66), consistent with moderate collinearity rather than independent effects. In this context, inclusion of both covariates introduces redundancy without improving model stability or interpretability. Importantly, inclusion of PMI did not materially

alter the results. Effect size estimates remained highly concordant between models with and without PMI adjustment (median Pearson r ≈ 0.93; see added comparison plot below), and the set of significant genes was largely unchanged. These findings indicate that much of the variance associated with PMI is already captured by kit batch. Therefore, adding PMI in addition to kit batch would largely account for overlapping sources of variation. We therefore retained kit batch as the primary covariate because it directly represents the technical source of variation, while avoiding overparameterization from inclusion of two correlated variables. To further address the reviewer's concern, we now include an MDS plot colored by kit batch in **Supplementary Fig. 5** and expanded the Methods section to explain the rationale for not including PMI in the spatial transcriptomics model (l. 252 ff). For multivariable analyses of key outcomes outside the spatial omics assays (l. 405 ff), PMI has now been included as an additional covariate. This adjustment did not materially affect the primary associations. We have also revised the Discussion to explicitly acknowledge PMI as a contributing source of variance and a study limitation, while emphasizing that the principal biological conclusions remain robust to this effect (l. 422 and l. 800 ff).

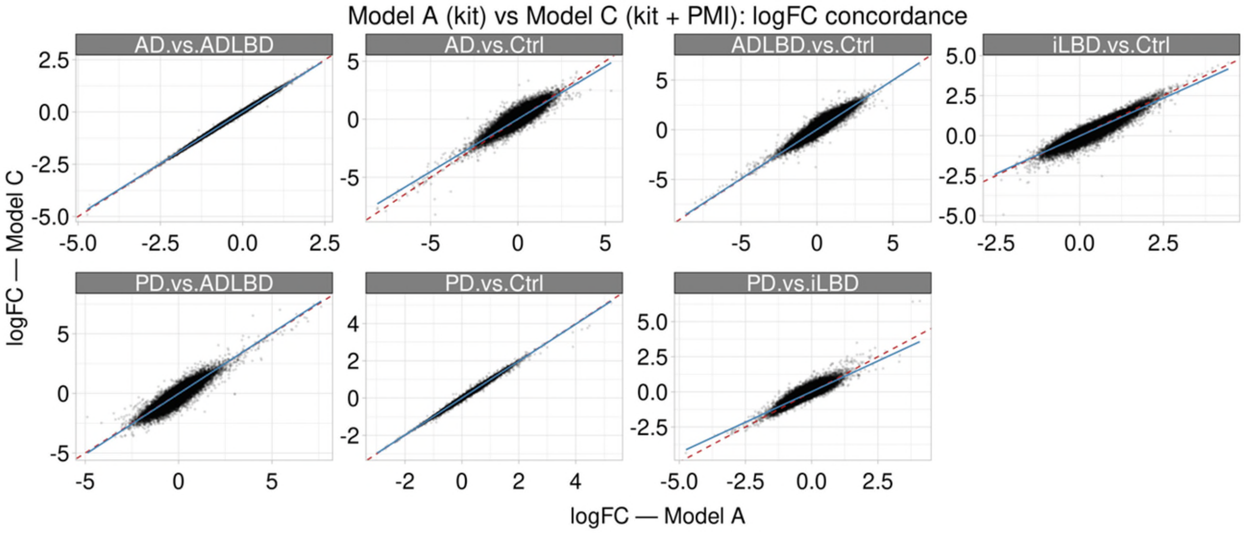

3. The additional C1QC staining provided in the response raises further concerns regarding antibody specificity. The near-complete overlap between C1QC and TH signal suggests the possibility of fluorescence bleed-through or channel crosstalk. This makes it difficult to interpret the specificity of the C1QC signal.

Response: We thank the reviewer for raising this important concern regarding potential fluorescence bleed-through or channel crosstalk between the TH and C1QC channels. To directly address this issue, we performed additional control experiments in which tissue sections were incubated with only a single primary antibody (either anti-C1QC or anti-

TH), while still applying both secondary antibodies simultaneously under identical imaging settings used for the experimental samples. As shown in the figure below, sections stained with either of the two anti-C1QC antibodies produced signal exclusively in the Alexa Fluor 647 channel and showed no detectable fluorescence in the Alexa Fluor 488 channel. Conversely, sections stained only for TH displayed signal exclusively in the Alexa Fluor 488 channel without detectable signal in the Alexa Fluor 647 channel. These controls demonstrate that the observed overlap between TH and C1QC in the experimental samples is not caused by fluorescence bleed-through, channel crosstalk, or secondary antibody cross-reactivity, but instead reflects genuine co-localization of the respective signals. The figure is also uploaded as a separate file ("**Antibody_Control.jpeg**") for optimized visibility.

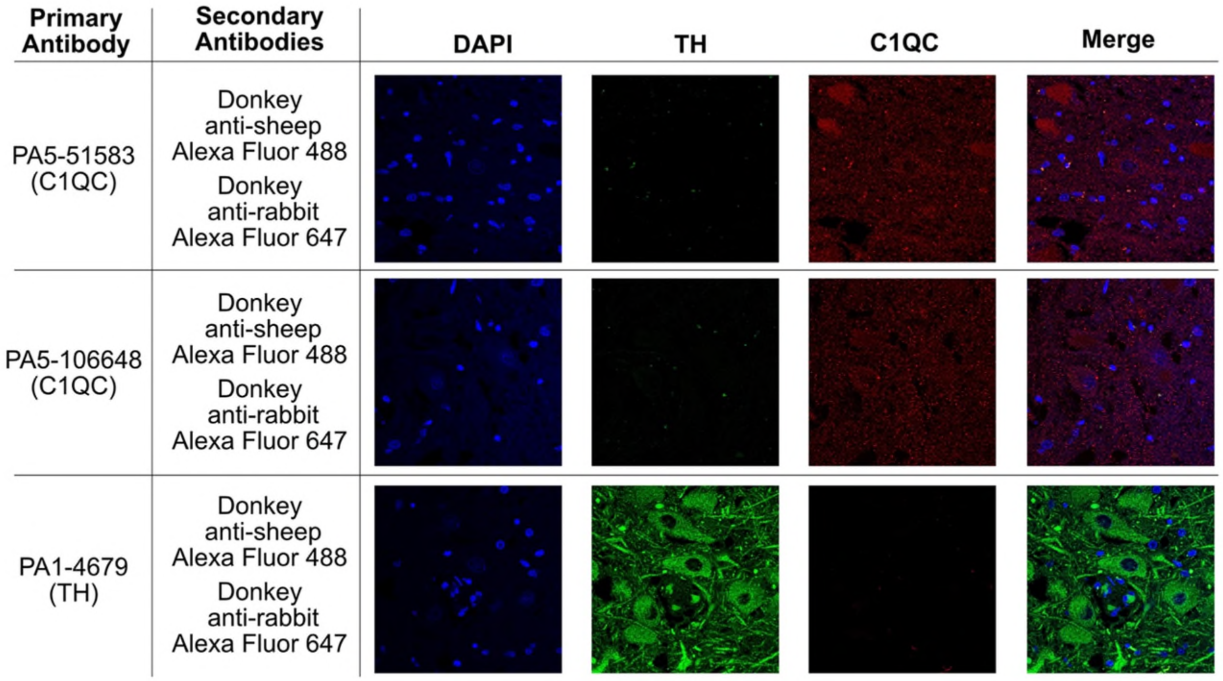

Control experiments excluding fluorescence bleed-through between TH and C1QC channels: To assess potential fluorescence bleed-through or channel crosstalk between TH and C1QC signals, control stainings were performed using sections incubated with only a single primary antibody together with both secondary antibodies. Sections stained with either anti-C1QC antibodies (PA5-51583 or PA5-106648) showed signal exclusively in the Alexa Fluor 647 channel, with no detectable fluorescence in the Alexa Fluor 488 channel. Conversely, sections stained only with anti-TH antibody (PA1-4679) displayed signal exclusively in the Alexa Fluor 488 channel, with no detectable fluorescence in the Alexa Fluor 647 channel. DAPI was used for nuclear counterstaining. Merged images

confirm the absence of detectable channel bleed-through or secondary antibody cross-reactivity under the imaging conditions used.

Reviewer #3:

In this manuscript, Rumpf, Strubing et al. examine the cellular and molecular changes that occur in the substantia nigra in different synucleinopathies. They utilize multiple techniques to extract spatial histopathological, transcriptomic, proteomic information from consecutive FFPE sections of patients with either incidental Lewy body disease (iLBD), Parkinson's disease (PD), Alzheimer's (AD), AD+LBD, and controls. From this rich collective dataset, they report specific signatures associated with each disease type, perhaps the most significant being the upregulation and enrichment of complement C1q in the SNpc of iLBD subjects. Surprisingly, this was not observed in the SNpc of subjects with AD+LBP despite these samples scoring having similar Braak LB staging and seed amplification assay (SAA) status. In follow up studies, they show increased C1q localization to ventral tier dopamine neurons of iLBD (but not AD+LBD), and C1q interaction with the GABAergic postsynaptic scaffold protein Gephyrin within CD68+ puncta, suggesting that they are engulfed in microglial lysosomes. The authors conclude that this neuroinflammatory response represents a unique and novel feature that distinguishes iLBD from AD+LBP, and the intriguing possibility that this occurs in the absence of frank Lewy pathology as this is not observed in AD+LBD. These findings therefore address the central question of whether AD+LBP and iLBD/PD follow distinct pathogenetic pathways and introduce a powerful methodological roadmap for examining this. On the other hand, there are a number of gaps in the data and interpretation in this current manuscript that need to be addressed in order to more fully substantiate the main claims made.

Main points:

- The observation that C1q is upregulated in SNpc of iLBD vs AD+LBP is an important one with potentially profound mechanistic and clinical implications. However, a detailed comparison with C1q status in the PD samples is crucial to establish whether this remains elevated or is transient. Either outcome would be significant but would provide important insights into whether iLBD/PD represents a distinct disease axis compared to AD+LBD or that C1q is a specific marker of only iLBD.

Response: We have directly addressed this question by staining additional sections from PD cases (n = 4). Our results showed no significant increase in C1QC in these cases (Fig. 5g,h), suggesting that the elevation in C1QC may be associated with early pathological changes and may be less prominent at later disease stages. We have

discussed the implications of this finding—particularly the possibility of a therapeutic window of opportunity—in the revised manuscript (l. 744 ff).

This has been adequately addressed.

The data in question should already be contained in Supp Fig 6, but none of the targets are labeled in this figure. Since the complement pathway is being invoked as being specific to iLBD (and perhaps PD), levels of other complement components should also be highlighted as they have been widely described in the context of AD. For example, was staining or PLA with C3 performed?

Response: We have updated the revised figure to explicitly highlight complement-associated targets, indicating both significant and non-significant findings across all examined contrasts (Supp. Fig. 10). In addition to core microglial markers, we annotated a broad range of complement pathway components that have been widely implicated in neurodegenerative disease, including AD. These targets include TYROBP; C1Q complex members (C1QA, C1QB, C1QC); upstream and downstream complement factors (C1R, C1S, C2, C3, C4A, C4B, C5, C5AR1); complement regulators (CD55); and complement-related immune receptors and signaling molecules (ITGAM, ITGB2, FCGR2B, AXL). With respect to C3 specifically, we additionally performed immunohistochemical analyses to quantify C3 abundance in iLBD versus PD or Ctrl cases. These analyses did not reveal a statistically significant difference, although a trend was observed (Supp. Fig. 14).

Supplementary Figure 14 shows staining of CD18, not C3.

Response: We thank the reviewer for noting this discrepancy. We confirm that **Supp. Figure 14** shows staining for CD18 (ITGB2), not C3. The reference to C3 in the response letter was an error. As noted in the manuscript (l. 592 ff) we selected CD18 as a readout of complement-associated microglial activity because it is a cell-associated receptor component that is stably expressed and directly mediates recognition of complement-opsonized targets. In contrast, C3 is a soluble, dynamic protein that is secreted, cleaved, and diffusely deposited, making its immunohistochemical detection more variable and harder to interpret, especially in post-mortem tissue. Focusing on CD18 (as part of CR3) therefore provides a more robust and spatially interpretable measure of complement-mediated microglial phagocytic activity.

Similarly, was C1q elevated in the neocortex or amygdala of AD+LBD?

Response: We thank the reviewer for this question. We did not assess C1q levels in the neocortex or amygdala, as cortical tissue was not available for the present study. Accordingly, our conclusions regarding C1QC are restricted to the substantia nigra, and we now explicitly note this limitation in the manuscript (l. 791 ff).

Adequately addressed.

Given the very significant differences in PMI and fixation time between AD and non-AD samples, these could also serve as an internal control for validating the integrity of the targets across samples from the two sites.

Response: Differences in post-mortem interval and fixation time were formally addressed in the multivariable regression analyses described above, in which post-mortem interval and age were included as covariates when modeling key molecular and histological outcomes (including pigmented neuron counts and SAA). In these models, neither PMI nor age significantly contributed to the observed group differences, and inclusion of these variables did not alter the main results. Consistent with this, RNA quality metrics (DV200) and molecular readouts were preserved in AD cases despite longer PMI and fixation times.

This does not appear to be accurate based on Supp. Fig. 5, where there are clear differences based on PMI.

Response: We agree that **Supp. Fig. 5** shows a visible PMI-associated gradient in the spatial transcriptomic data, and we should have framed our original response more carefully. In line with the reviewer's suggestion, we evaluated whether PMI-related variation could serve as an internal control for sample integrity. However, because PMI is strongly confounded with both sample provenance and Visium v2 kit batch, its effects cannot be interpreted independently. Instead, we assessed the robustness of our findings in two complementary ways: (i) by including PMI in multivariable models and (ii) by directly comparing results obtained with and without PMI adjustment (see also our response to Reviewer 2, point 2). These analyses demonstrated highly concordant effect sizes and minimal changes in the set of significant findings, indicating that the observed molecular and histological signals are preserved across the PMI range and are unlikely to be driven primarily by degradation-related artifacts. We have revised the manuscript to clarify that PMI contributes to overall variance in the dataset, while emphasizing that the principal biological conclusions remain robust to PMI adjustment.

- LB dementia (DLB) also features dopamine neuron loss but discussion about it is notably absent from the study. Since one of the major conclusions is that SNpc neuron loss/C1q activation is not correlated with synucleinopathy, please provide a rationale that the DLB samples were not examined.

Response: The absence of a dedicated DLB cohort in the present study reflects cohort availability and study scope rather than a conceptual exclusion of DLB from the biological framework. Our study was specifically designed to compare PD with iLBD and with AD cases with and without Lewy body co-pathology, thereby enabling us to disentangle α-synuclein–associated changes from AD-related processes within a well-defined anatomical context. Inclusion of DLB would require a sufficiently powered and neuropathologically homogeneous cohort with detailed staging of both cortical and

brainstem pathology, which was not available within the constraints of the present study. Moreover, DLB is characterized by substantial cortical Lewy pathology and clinical dementia, raising additional variables beyond the midbrain-focused questions addressed here. Importantly, our conclusions are restricted to the disease contexts examined and are not intended to generalize to all synucleinopathies, including DLB. We now explicitly acknowledge this limitation in the Discussion and note that extending the present spatial multi-omics approach to DLB represents an important direction for future work (l. 796 ff).

Appropriately addressed.

- Line 517: "Region-Specific Upregulation of C1QC in iLBD Suggests Early, αSyn-Independent Inflammatory Signaling." This is a bold and exciting hypothesis and based largely on the observation here that LBP, when present in AD, does not present with this signature nor dopamine cell loss. However, an important caveat is that LBP is present in iLBD - thus, an equally valid interpretation is that LBP is not sufficient (but possibly still necessary) for this phenomenon.

Response: We clarify our operational definition of iLBD: In line with prior work,12 iLBD cases in this study were defined as Lewy body Braak stage ≤ 2, a stage at which LBP is restricted to lower brainstem regions and is absent from the substantia nigra pars compacta. Thus, the region-specific upregulation of C1QC observed in the substantia nigra in iLBD occurs in the absence of local Lewy body pathology and precedes overt dopaminergic neuron loss which is also shown in Fig. 1c,d. In contrast, AD cases with LB co-pathology exhibit higher Braak stages with established LBP yet do not show this C1QC signature. Together, these observations support the interpretation that early C1QC upregulation in the substantia nigra reflects inflammatory signaling that is not primarily driven by visible αSyn pathology per se. We have revised the text to clarify this distinction and to avoid overstatement of αSyn independence (l. 747 ff).

The revised text could not be found at line 747, and the question is not fully addressed. While iLBD is mostly negative by PLA, several of these cases are positive by SAA, suggesting that the lack of α-synuclein pathology in iLBD substantia nigra may be a result of detection sensitivity, not independent of C1QC and α-synuclein pathology.

Response: We thank the reviewer for this important and nuanced point. We agree that our previous wording may have overstated the independence of C1QC upregulation from αSyn pathology. While iLBD cases in our study lack detectable Lewy body pathology in the substantia nigra by conventional immunohistochemistry and PLA (**Fig. 1c,d**), several cases show seeding activity by SAA, indicating the presence of low-level or early αSyn pathology below the detection threshold of histological methods and reinforcing iLBD as a subtle prodromal stage of manifest PD. We have slightly revised the text to clarify that our data do not exclude a contribution of early or sub-threshold αSyn species to C1QC

upregulation, although we found no correlation between C1QC and the SAA result (**Fig. 5h,i**) Rather, our findings indicate that C1QC upregulation occurs prior to overt Lewy body formation and in the absence of detectable aggregated αSyn by standard histopathological methods, and is not observed in AD+LBP cases despite substantial Lewy pathology. Accordingly, we now frame our conclusion a bit more cautiously, emphasizing that αSyn pathology alone is not sufficient to drive this signature, while a contributory role of early or diffusible αSyn species cannot be excluded (l. 574 & 748). Importantly, this strongly supports the validity of iLBD as a PD prodromal stage.

- Fig 6: The observation that Gephyrin colocalizes to microglial CD68+ lysosomes is interesting. However, the Gephyrin+ objects appear to be much larger than the CD68 puncta, which makes it hard to reconcile as inside the lysosomal lumen.

Response: We have replaced the representative images in Figure 6 with higher-quality examples that more clearly illustrate the spatial relationship between gephyrin and CD68-positive microglial lysosomal structures. The revised images better demonstrate the association of gephyrin signal with CD68-positive compartments, addressing the previous concern regarding the apparent size discrepancy between gephyrin-positive objects and CD68 puncta.

Addressed adequately.

Other points:

- The use of multiple spatial-omics (transcriptomics, proteomics), histological (IHC, PLA), and biochemical (SAA) approaches on consecutive tissue samples is a core strength of the manuscript. This should be emphasized by showing data from consecutive (or nearby) sections in Fig 1b-d so that readers can appreciate the correlation between these sources of data. The images currently shown do not appear to be taken from the same sample/subject.

Response: To better illustrate this workflow, we have adapted Figure 1 to include a schematic outlining the use of consecutive tissue sections for the spatial transcriptomic, spatial proteomic, and biochemical assays. Conventional IHC and PLA experiments, however, were not performed on directly consecutive sections but instead were conducted on representative sections derived from the same cohort. As a result, these assays cannot be shown as directly consecutive sections in Figure 1. We have clarified this point in the figure legend to ensure transparency while still highlighting the complementary nature of these datasets.

Adequately addressed.

- Line 382: "In contrast, these associations were attenuated or lost in AD and AD+LBP cases (Supp. Fig. 3f,h)." The lack of associations could also be due to the

small sample size for AD and AD+LBP cases. Also, for equal comparison, please include control samples in Supp Fig. 3f.

Response: We agree with the reviewer that the absence of significant associations in the AD and AD+LBP groups may, in part, reflect limited statistical power due to smaller sample sizes. We have clarified this limitation in the revised text and now explicitly frame these analyses as exploratory in the AD-spectrum cohorts. In addition, as suggested, we have included control samples in Supp. Fig. 3f to enable a more balanced comparison across disease groups.

Adequately addressed.

- Fig. 3i: The correlations for the PD comparisons seem to be driven primary by a cluster with minimal pigmented neuron loss. This should be mentioned in text

Response: We thank the reviewer for this observation and would like to clarify that the apparent "cluster" in Fig. 3i does not represent a subset of spatial regions or tissue areas with minimal dopaminergic neuron loss. This analysis is restricted to a single spatial domain (SNpc) defined by BASS clustering, and each point represents an individual differentially expressed gene rather than a cellular or anatomical cluster. The x-axis shows the correlation between gene expression and pigmented neuron counts across spatial spots within this domain, while the y-axis reflects the group-wise $\log_2$ fold-change for each gene. The aggregation of points therefore reflects a group of genes with similar relationships to dopaminergic neuron abundance, not a subset of spots with preserved neurons. Importantly, the absence of such correlations in the AD+LBP comparison indicates that differential expression in this condition is largely independent of dopaminergic neuron loss.

The explanation is noted, but does not support that differential expression is independent of dopaminergic neuron loss. Rather, there are two populations of genes. Those genes that show decrease in PD and those that show increase in PD. Those that show decrease largely have a higher association of genes and neuron loss, whereas genes that are increased show no or negative association with pigmented neuron loss. In AD, there are mostly only increased expression of genes, so they cluster near the top of the plot. Correlating these different measures does not seem useful and the conclusion it is used for seems unsupported.

Response: We thank the reviewer for this thoughtful interpretation, which we find well justified. We agree that the pattern observed in **Fig. 3i** reflects two groups of genes with opposing directions of differential expression: genes downregulated in PD tend to show a stronger association with dopaminergic neuron loss, whereas upregulated genes show weaker, absent, or even negative associations. Our intention with this analysis was not to demonstrate that differential expression is independent of neuronal loss, but rather to

provide a descriptive comparison of how gene expression changes relate to dopaminergic neuron abundance across disease contexts. However, we agree with the reviewer that this analysis does not substantially strengthen the conclusions and may instead complicate interpretation. We have therefore removed **Fig. 3i** and the corresponding text and interpretation from the revised manuscript.

- Line 601: "This correlation further supports the potential of SAA as a reliable biomarker for pathology progression and staging in clinical and neuropathological settings." The authors should clarify that the described correlations are for postmortem tissue SAA, which have important neuropathological applications, but that CSF SAA is most often used in clinical settings and tend to be more sensitive when neocortical LBP is present.

Response: We thank the reviewer for this important clarification. We agree that the correlations described in this study are based on α-synuclein seed amplification assays performed on postmortem tissue and are therefore most directly relevant to neuropathological staging and postmortem assessment of disease progression. We have revised the text to explicitly distinguish these applications from clinical CSF-based SAA, which is more commonly used in clinical settings and typically shows higher sensitivity in the presence of neocortical Lewy body pathology. This distinction is now clearly stated in the revised manuscript (l. 625 ff)

This passage would benefit from SAA citations.

Response: We thank the reviewer for this suggestion. We have added appropriate references to the revised paragraph (l. 638 ff) supporting the use of αSyn SAA in both postmortem tissue and CSF, including studies demonstrating their diagnostic performance, neuropathological relevance, and the application of quantitative SAA approaches. These additions clarify the distinction between tissue-based and clinically applied CSF-based assays.
